# Striking transformations of the hydroborylene ligand in a HB:→Ni^II^ complex with isocyanides and CO†
†Electronic supplementary information (ESI) available: Experimental procedures and characterisation data for all new compounds, full details of computational studies. Crystal data, details of data collections and refinements. CCDC 1579154–1579160. For ESI and crystallographic data in CIF or other electronic format see DOI: 10.1039/c7sc04792d


**DOI:** 10.1039/c7sc04792d

**Published:** 2018-02-05

**Authors:** T. J. Hadlington, T. Szilvási, M. Driess

**Affiliations:** a Department of Chemistry , Metalorganics and Inorganic Materials , Techniche Universität Berlin , Strasse des 17. Juni 135, Sekr. C2 , 10623 Berlin , Germany . Email: matthias.driess@tu-berlin.de; b Department of Chemical & Biological Engineering , University of Wisconsin-Madison , 1415 Engineering Drive , 53706 , Madison , WI , USA

## Abstract

For the first time, the reactivity of the metal- and N-heterocyclic carbene-supported monovalent hydroborylene is reported. Isocyanides react with the hydroborylene Ni^II^ complex [{cat(^TMS^L)Si}(Cl)Ni←:BH(NHC)_2_] **1** (cat = *ortho*-C_6_H_4_O_2_; ^TMS^L = N(SiMe_3_)(Dipp); Dipp = 2,6-Pr^i^_2_C_6_H_3_; NHC = :C[(Pr^i^)NC(Me)]_2_) to form the hydride-bridged hydroborylene-Ni^II^ complexes **2**. The reaction of **1** with isoelectronic CO, however, is reversible and furnishes the related unprecedented hydride- and CO-bridged hydroborylene Ni^II^ complex **2-CO**, which undergoes isomerisation through silyl/NHC exchange at ambient temperature to afford the corresponding hydro(silyl)boryl Ni^II^ complex **3**. Markedly, **2** readily and quantitatively react with one further molar equiv. of isocyanide to give, under borylene liberation and H/Cl ligand exchange, boraketiminium species, which represent cationic B^I^ complexes. These latter compounds are highly reactive in solution, and can undergo quantitative transformation into previously unknown cyanoborenium cations.

## Abstract 2

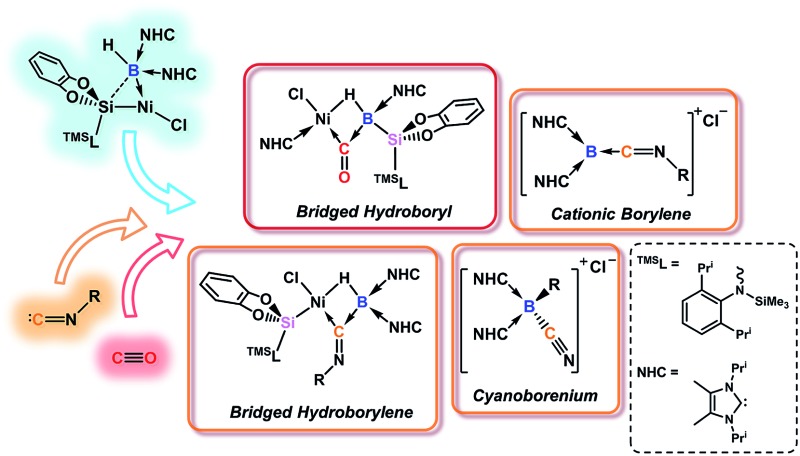
The first reactivity of the hydroborylene ligand is described, giving access to a remarkable range of unprecedented boron-centered species.

## Introduction

**Fig. 1:**
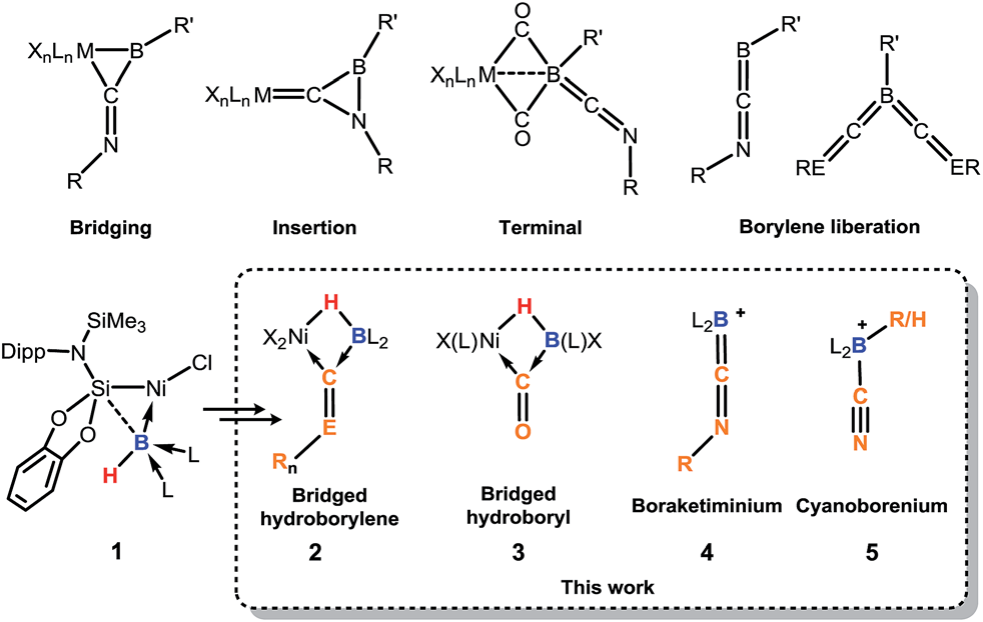
Selected reaction modes of isocyanides or CO towards borylene transition-metal complexes, and related chemistry of **1** to give the novel products **2–5**. R, R′ = organic groups; L = donor ligand; X = anionic ligand; E = N, *n* = 1; E = O, *n* = 0.

Since the landmark work of H. C. Brown in the synthesis and reactivity of hydroboranes,^1^ exceptional progress has been made in the broader field of synthetic boron chemistry. In catalytic applications boron is a *tour de force*, with, for example, asymmetric hydroboration,^2^ C–H borylation,^3^ and C–C coupling reactions (*viz.* the Suzuki reaction)^4^ all now common place in synthetic laboratories, cementing the importance of boron as a versatile chemical building-block. More fundamentally, isolable molecular species of multiply-bonded boron in the 0,^5^ and +1,^6^ oxidation states can now be accessed, giving insights into the nature of the B–B multiple bond,^7^ and giving further access to otherwise unattainable boron-containing molecules.^5^,^6^ Borylene chemistry has also seen substantial attention, initiated in 1995 by Braunschweig and co-workers in the first report of an isolable molecular borylene complex, stabilised in the coordination sphere of a transition-metal (TM),^8^ followed soon after by the first terminal borylene complex.^9^ Considerable work to this end has also been reported by Aldridge *et al.*, particularly in the synthesis of borylene complexes through B–H activation.^10^ Since this time, borylene chemistry has blossomed, and given access to otherwise challenging boron-containing compounds, for example through B–B and B–C bond coupling processes.^11^ Nevertheless, until very recently, the parent borylene (*i.e.*: BH) was unknown, when Bertrand and co-workers reported that the synthesis of compounds of the general formula L_2_BH is possible (L = cAAC or NHC; cAAC = cyclic(alkyl)(amino) carbene; NHC = N-heterocyclic carbene), utilising the powerful σ-donation properties of these ligands to complete the octet at boron(i).^12^ More recently, we reported on the first example of a terminally coordinated :BH ligand, in the hydroborylene Ni^II^ complex [{cat(^TMS^L)Si}(Cl)Ni←:BH (NHC)_2_], **1** (cat = *ortho*-C_6_H_4_O_2_; ^TMS^L = N(SiMe_3_)(Dipp); Dipp = 2,6-Pr^i^_2_C_6_H_3_; NHC = :C[(Pr^i^)NC(Me)]_2_).^13^ Related terminal borylene-TM complexes have been shown to undergo diverse and remarkable reactivity towards isocyanides and carbon monoxide,^11^,^14^,^15^ including the multiple coordination of CO at a B^I^ centre (Fig. 1).^16^ Thus, boron continues to show enormous potential in countless aspects of chemistry, allowed by the development of modern synthetic methodologies and boron-centred building-blocks. In this light, the synthetic utility of hydroborylenes has so far gone unexplored due to their being only recently realised as stable compounds.^12^,^13^ We therefore sought to explore the use of hydroborylene Ni^II^ complex **1** as a boron building-block in synthetic chemistry, and to further explore the effect of the hydride ligand in **1** in coordination chemistry. Here we report the striking reactivity of **1** towards CO and organo-isocyanides (CNR), leading to the Ni-assisted formation of several novel boron-centred functional groups which would otherwise prove extremely difficult to attain *via* traditional synthetic strategies, and are made viable due the presence of the hydride ligand and Ni^II^ centre in **1**.

## Results and discussion

**Scheme 1:**
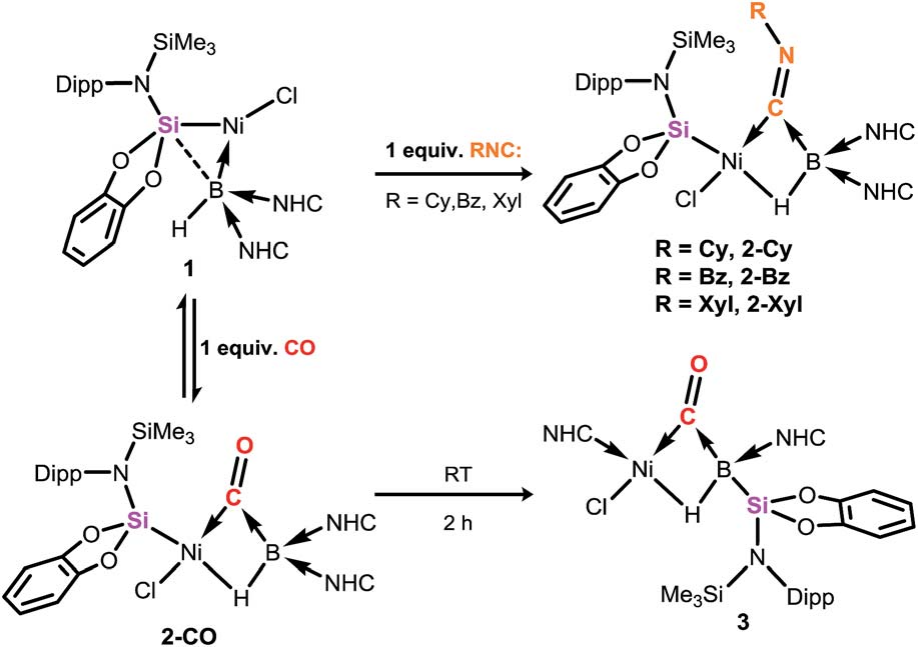
Addition of one molar equiv. of isocyanides and CO to the hydroborylene Ni^II^ complex **1**, furnishing the μ-hydride, μ-CO borylene and silylboryl Ni^II^ complexes **2** and **3**, respectively. Anagostic interactions in **3** are not shown. NHC = C[(Pr^i^)NC(Me)]_2_.

**Fig. 2:**
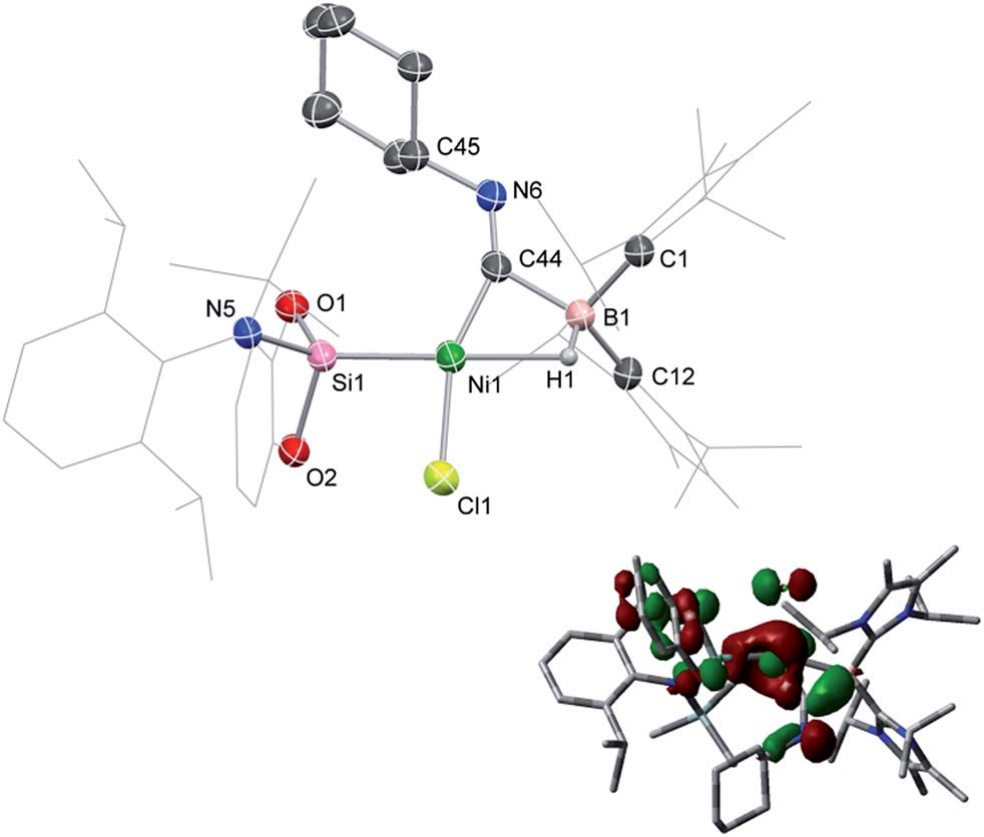
Molecular structure of **2-Cy** with thermal ellipsoids at 30% probability. Selected distances (Å) and angles (°): B1–Ni1 2.235(6); B1–C44 1.63(1); Ni1–C44 1.833(5); Ni1–Si1 2.186(2); C44–N6 1.261(7); Ni1–C44–N6 152.2(5); B1–C44–N6 127.0(5); B1–C44–Ni1 80.0(3); B1–Ni1–Cl1 114.3(2); Si1–Ni1–C44 104.3(2); Si1–Ni1–Cl1 94.52(7); inset: the HOMO of **2-Cy**.

Compound **1** contains a three-coordinate, 16-electron Ni^II^ centre,^13^ which suggests a high reactivity towards donor ligands such as CNR and CO. Accordingly, addition of one molar equiv. of cyclohexyl isocyanide (CNCy) to a deep blue THF solution of **1** at –78 °C resulted in an immediate color change to deep orange. A single-crystal X-ray diffraction analysis of deep orange-yellow crystals grown from this reaction mixture revealed that **2-Cy** (Scheme 1, Fig. 2) is formed, in which, remarkably, both the hydride and isocyanide ligands bridge the Ni and B centres.‡
‡The broad resonance in ^11^B NMR spectrum of **2-Cy** (*δ* = –43.0 ppm) clearly correlates to a signal in its ^1^H NMR spectrum as shown through ^1^H,^11^B HMQC NMR analysis (*δ* = 2.34 ppm; see Fig. S3 in ESI†), corroborating retention of the B–H bond. Substantial Ni–B bond weakening is apparent, due to this marked conformational change at boron (for **2-Cy**: *d*(B1–Ni1) = 2.230(2) Å, MBO_B–Ni_ = 0.48; For **1**: *d*(B1–Ni1) = 2.015(2), MBO_B–Ni_ = 0.76). This results in a tetrahedral B^I^ centre whose lone-pair of electrons is directed towards the π*-orbital of the CNCy ligand (*d*(B1–C44) = 1.637(3) Å, MBO_B–C_ = 0.75), resulting in considerable C=N bond weakening in this fragment. The calculated HOMO for **2-Cy** comprises largely of this bonding interaction, as well as bonding contributions from Ni (Fig. 2, inset). In line with this, the IR spectrum of **2-Cy** displays a drastically lower stretching frequency for this bond (*ν*_C=N_ = 1626 cm^–1^) when compared with related reported species (*viz.* [CpMn(CO)_2_-η^2^(CNR)-B(Bu^*t*^)(NHC′)], *ν*_C=N_ = 1856–1930 cm^–1^; R = Me, Cy; NHC′ = C[(Me)NC(Me)]_2_).14*b*§
§The calculated value, ν_C=N_ = 1655 cm^–1^, in **2-Cy** is in line with that observed experimentally. The low Mayer bond order (MBO) for the B–C(NCy) bond suggests little degree of CyNC:→B donation. Conversely, some back-bonding from the Ni^II^ centre to the CyNC ligand in **2-Cy** is observed (*d*(Ni1–C44) = 1.820(2) Å, MBO_C–Ni_ = 1.12), further evidenced by the relatively linear Ni1–C44–N6 angle (∠_NiCN_ = 153.0(1) °) when compared to the B1–C44–N1 angle of 126.3°. Thus, the bonding model outlined in Scheme 1 best describes this unusual isocyanide borylene complex, and represents a fascinating new isomeric form for TM-borylene complexes, largely owing to the presence of the bridging hydride ligand in **2-Cy**.

Addition of related ligands (*i.e.* CNR with R = 2,6-Xyl, Bz) to THF solutions of **1** resulted in the formation of essentially isostructural μ-hydride, μ-CNR hydroborylene Ni^II^ complexes, **2-Xyl** and **2-Bz**, respectively (Scheme 1; Fig. S5 (R = Bz) and S6 (R = 2,6-Xyl) in ESI†). Both **2-Xyl** and **2-Bz** contain bonding interactions that are similar to those in **2-Cy**. That is, considerable weakening of the B–Ni and isocyanide CN bonds is observed, the latter being corroborated by their stretching vibrational frequencies (**2-Xyl**: *ν*_C=N_ = 1634 cm^–1^; **2-Bz**: *ν*_C=N_ = 1627 cm^–1^). Their ^11^B NMR spectra are also very similar to that for **2-Cy**, each showing a single broad resonance. Complexes **2** are poorly soluble in common organic solvents, and decompose to a complex mixture over the course of one day, but are stable in the solid state for an indefinite period of time. Important geometrical and spectroscopic parameters are summarised in Table 1.¶
¶B–H stretching vibrational bands were not observed for these species, presumably as they were too weak. The presence of the B–H fragments was confirmed with ^1^H,^11^B HMQC NMR analyses (see above).


**Table 1:** Selected distances (Å), angles (°), IR stretching frequencies (cm^–1^), and ^11^B NMR shifts in d_8_-THF (ppm) for **2** and **3**, and in d_2_-DCM for 2-CO

	**2-Cy**	**2-Xyl**	**2-Bz**	**2-CO**	**3**
Ni1–B1	2.235(6)	2.289(3)	2.235(6)	—	2.235(2)
Ni1–C44	1.833(5)	1.805(3)	1.819(9)	—	1.780(1)
B1–C44	1.63(1)	1.629(5)	1.65(1)	—	1.652(2)
Ni1–C44–N6	152.2(5)	144.3(2)	125.2(7)	—	—
Ni1–C44–O3	—	—	—	—	142.6(1)
B1–C44–N6	127.0(5)	127.1(2)	153.0(7)	—	—
B1–C44–O3	—	—		—	136.2(1)
ν_C=N/O_	1626	1634	1623	1694	1720
*δ* ^11^B NMR (^1^*J*_BH_)	–43.1	–41.4	–43.3	–42.6	–57.8 (54 Hz)

**Fig. 3:**
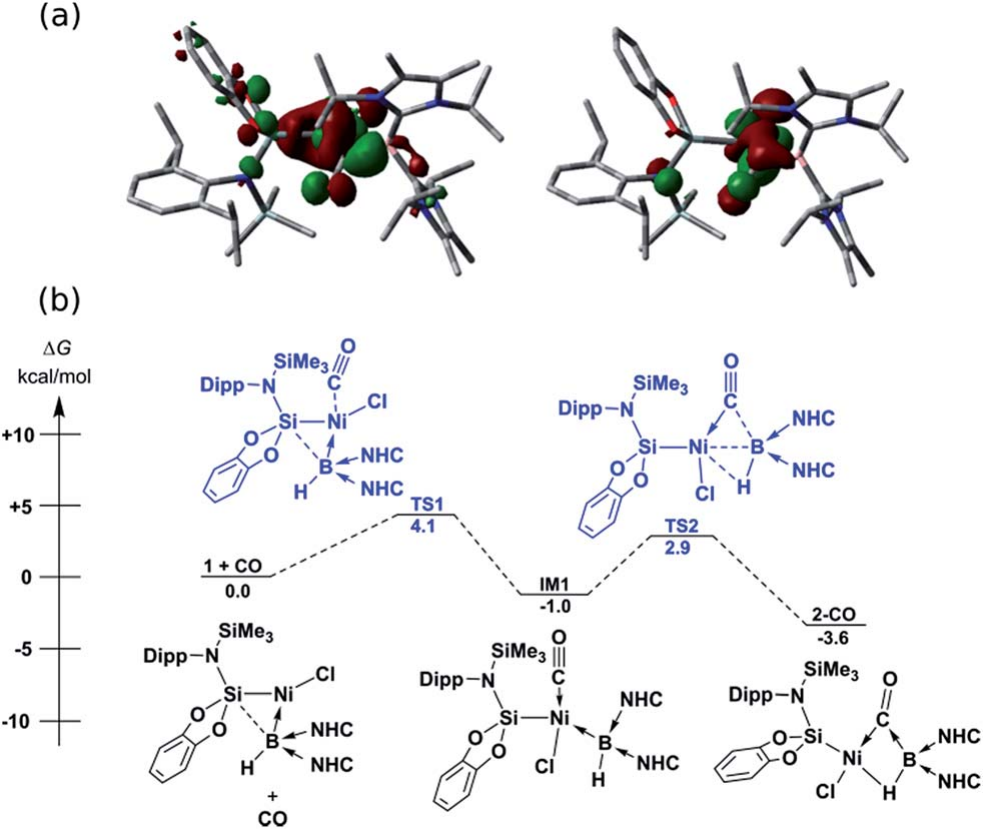
(a) HOMO (left) and HOMO-5 (right) of **2-CO**; (b) DFT derived pathway for the reversible reaction between **1** and CO. NHC = C[(Pr^i^)NC(Me)]_2_).

In contrast to reactions with isocyanides, **1** undergoes a remarkable reversible reaction with CO, generating the μ-hydride, μ-CO adduct **2-CO** (Scheme 1) as an equilibrium mixture with **1**, representing a rare example of the intermolecular activation of CO with a boron compound.^16^,^17^ Addition of an excess of CO to **1** leads to decomposition, however **2-CO** can be crystallised by addition of one molar equiv. of CO to **1** at –78 °C, followed by layering with hexane and storage at –30 °C. Although the quality of these crystals did not allow for collection of publishable data, they did confirm the connectivity of **2-CO**. Crystals of **2-CO** are insoluble in common aprotic organic solvents but dissolve in d_2_-DCM. Its solutions in d_2_-DCM at –60 °C yield a broad resonance in the ^11^B NMR spectrum at *δ* = –42.6 ppm; the latter chemical shift is similar to those for isocyanide derivatives. The bridging nature of the hydride ligand in **2-CO** is borne out by the lower *J*-coupling value for its BH fragment in comparison with that for **1** (^1^*J*_BH_ = 101 Hz). The stretching vibrational frequency for the CO ligand in **2-CO** indicates significant lowering of the CO bond order (*ν*_CO_ = 1694 cm^–1^) relative to free CO. Despite this, the reaction of **1** with CO is reversible, with storage of suspensions of **2-CO** in THF yielding small amounts of crystalline **1** after one day. Observation of the frontier orbitals of **2-CO** reveals that both the HOMO and HOMO-5 involve considerable donation to the π* orbital of the CO ligand (Fig. 3(a)), in a similar manner to the bonding situation discussed for **2-Cy**. A DFT analysis of the reaction mechanism of **1** with CO (Fig. 3(b)) indicates that initial coordination of CO occurs at the Ni^II^ centre of **1***via***TS1** (4.1 kcal mol^–1^), leading to the four-coordinate Ni^II^ complex **IM1** (–1.0 kcal mol^–1^). Subsequent coordination of the B^I^ centre to the CO ligand (**TS2**, 2.9 kcal mol^–1^) leads to weakening of the B–Ni bond and B–C bond formation, generating **2-CO** (–3.6 kcal mol^–1^). The negligible thermodynamic barriers and minimal energy gain upon binding CO, then, explain the reversibility of this reaction. Whilst some degree of B–Ni bonding is present in **2-CO**, calculated bond orders for this interaction (Wiberg Bond Index (WBI)_B–Ni_ = 0.17; MBO_B–Ni_ = 0.30) are considerably lower than those for both the B–C (WBI_B–C_ = 0.81; MBO_B–C_ = 0.67) and Ni–C bonds (WBI_C–Ni_ = 0.95; MBO_C–Ni_ = 1.05). Notably, these calculated MBO values for the Ni–C and B–C bonds in **2-CO** are somewhat lower than those for the closely related bonding interactions in **2-Cy**, again yielding an explanation for the reversibility of the reaction of **1** with CO.

**Fig. 4:**
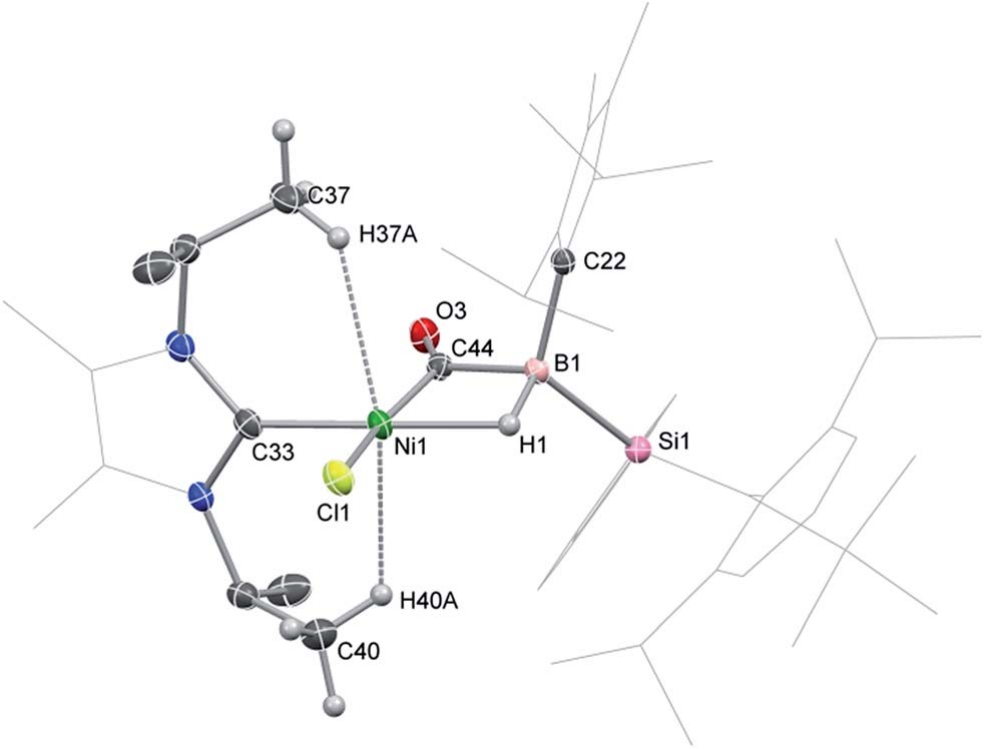
Molecular structure of **3**, with thermal ellipsoids at 30% probability. Selected bond lengths (Å) and angles (°): B1–Ni1 2.235(2); B1–C44 1.652(2); Ni1–C44 1.780(1); O3–C44 1.197(2); Ni1–H37A 2.5585; Ni1–H40A 2.4199; B1–C44–Ni1 81.17(8); B1–C44–O3 136.2(1); Ni1–C44–O3 142.6(1).

**2-CO** is stable in the solid state for weeks. However, upon warming THF solutions of *in situ* generated **2-CO** to ambient temperature, a new compound is formed as a mixture with **1** after 2 h. Repeated re-crystallisations of this reaction mixture led to crystalline samples of the new product contaminated with small amounts of **1** (Fig. S19 and S20, ESI†). Nevertheless, an X-ray diffraction analysis of single-crystals isolated from this mixture revealed that, through a remarkable silyl/NHC exchange, the first-row TM hydroboryl complex **3** is formed (Scheme 1, Fig. 4). Notably, such hydroboryl complexes are extremely rare even for the heavier TMs, and are typically accessed through formal boron reduction.^18^ As in complexes **2**, both the CO and hydride ligands in **3** bridge the nickel and boron centres. The ^11^B NMR spectrum of **3** contains a doublet resonance at *δ* = –57.2 ppm (^1^*J*_BH_ = 56 Hz), considerably more shielded than that for **2-CO** most likely due to a greater charge density residing on the formally anionic boron centre in **3**. The CO stretching vibrational band in the IR spectrum of **3** is higher relative to that in **2-CO** (*ν*_CO_ = 1720 and 1694 cm^–1^, respectively) indicative of reduced back-bonding from boron to CO in transitioning from a borylene to a boryl ligand. The greater stability of **3** over **2-CO** may be owed to the NHC ligand now on Ni^II^, whose Pr^i^ groups allow for an octahedral geometry at nickel through anagostic interactions with two flanking CH_3_ groups.^19^

**Scheme 2:**
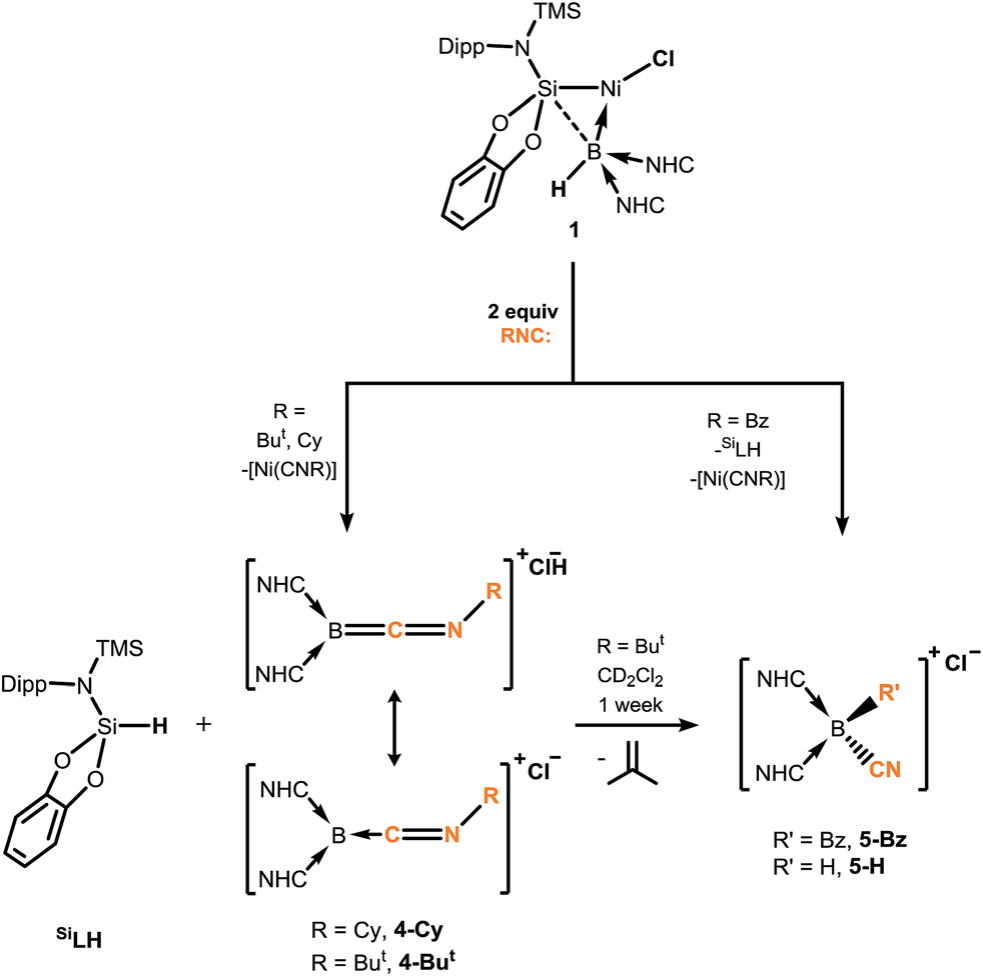
Addition of isocyanides to hydroborylene Ni^II^ complex **1**, forming boraketiminium and cyanoborenium cationic species.

**Fig. 5:**
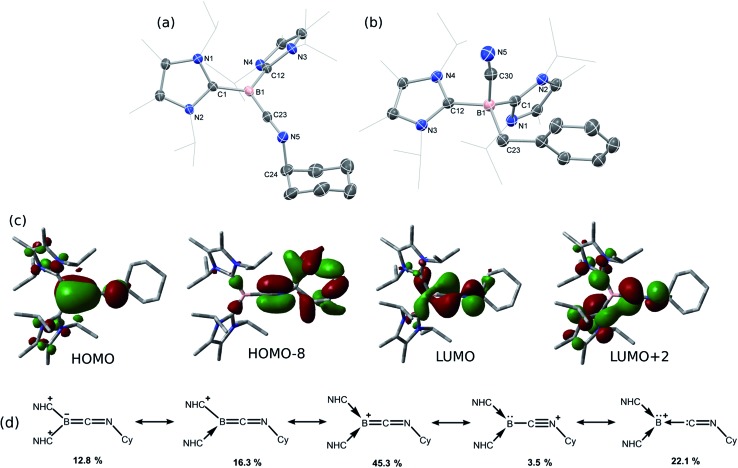
Molecular structures of (a) **4-Cy** and (b) **5-Bz**, with thermal ellipsoids at 30% probability, and chloride counter ions omitted. Selected bond lengths (Å) and angles (°) for the cation in **4-Cy**: B1–C23 1.467(4); C23–N5 1.221(4); B1–C1 1.572(4); B1–C12 1.583(4); B1–C23–N5 176.8(3); C23–N5–C24 121.2(3); for the cation in **5-Bz**: B1–C30 1.600(7); C30–N5 1.142(7); B1–C23 1.656(8); B1–C1 1.654(7); B1–C12 1.666(6); B1–C30–N5 170.6(5); (c) graphical representations of the HOMO (–6.11 eV), HOMO-8 (–9.48 eV), LUMO (–3.57 eV) and LUMO+2 (–2.82 eV) of 4-Cy; (d) results of the natural resonance theory analysis of **4-Cy**. Arrows indicate the no-bond resonance structures.

Braunschweig and co-workers have previously reported that addition of multiple equivalents of CNR or CO to borylene TM complexes can affect complete borylene-TM bond cleavage.14b,^16^ The addition of an excess of CO to **1** leads to the formation of dark precipitates and silent ^11^B NMR spectra. Conversely, addition of two molar equiv. of CNR (R = Cy, Bu^*t*^) to **1** gives rise to complete borylene liberation as well as an unexpected H/Cl ligand exchange, giving facile and quantitative access to extraordinary three-coordinate boraketiminium complexes, [{(NHC)_2_BCNR}^+^Cl^–^] (Scheme 2; R = Cy, **4-Cy**; R = Bu^*t*^, **4-Bu*^t^***), which can also be described as three-coordinate B^I^ cations.^20^ Monitoring the reaction progress by multinuclear NMR spectroscopy revealed that the liberated [^Si^LNiH] complex fragment (Scheme 2) undergoes decomposition to give the 'free' ligand, ^Si^LH (Fig. S24, S25; ESI†). Notably, a singlet resonance is observed in the ^11^B NMR spectrum at *δ* = –15.4 ppm, in keeping with that for recrystallised **4-Cy** (Fig. S26, ESI†). The solid-state structures of these boraketiminium compounds (**4-Cy**: Fig. 5; **4-Bu*^t^***: Fig. S39 in ESI†) encompass a planar λ^3^-B atom and a nearly linear BCNR moiety, indicative of multiple bonding character between these centres. The short B–C (**4-Cy**: *d*(B1–C23) = 1.467(4); **4-Bu*^t^***: *d*(B1–C23) = 1.433(2) Å) and C–N (**4-Cy**: *d*(C23–N5) = 1.221(4); **4-Bu*^t^***: *d*(C23–N5) = 1.220(2) Å) bonds are also in keeping with this, supported by a DFT analysis of the frontier orbitals in **4-Cy**, which are indicative of π-bonding between these centres (Fig. 5(c)). Surprisingly, the prospect of a formally B^III^ centre in reported species related to **4-Cy** and **4-Bu*^t^*** was not discussed in those publications, and instead the CNR or CO fragments were treated as neutral donor ligands.^20^ A computational natural resonance theory (NRT) study of **4-Cy** (Fig. 5(d)) suggests that the remarkable cationic 3-coordinate borylene resonance form of this complex, featuring a donor–acceptor bond between B^I^ and its isocyanide ligand, accounts for 22.1% of its ground state structure, whilst the formal bis(NHC) boraketiminium form is more prominent (45.3%), corroborating that **4-Cy** indeed has a degree of B^I^ character. The striking further reactivity of **4-Bu*^t^***, which is reminiscent of low-valent boron and group 14 chemistry,^21^,^22^ highlights this; over the course of 1 week, the ^1^H NMR spectrum of CD_2_Cl_2_ solutions of **4-Bu*^t^*** indicates the loss of isobutene, and the clean formation of a single species containing a B–H fragment (^11^B NMR: *δ* = –31.1, ^1^*J*_BH_ = 91 Hz), giving strong evidence for the formation of the cyanoborenium cation, [{(NHC)_2_B(H)(CN)}^+^Cl^–^] **5-H**. Remarkably, addition of two equiv. of CNBz to **1** directly leads to the benzyl derivative of **5-H**, [{(NHC)_2_B(Bz)(CN)}^+^Cl^–^] **5-Bz**, *via* C–N bond cleavage of the boraketiminium/borylene cation intermediate. The molecular structure of **5-Bz** confirms the formation of a terminal cyanoborenium complex (Fig. 5(b)), with a considerably contracted C30–N5 bond (*d* = 1.142(7) Å) when compared with the terminal C=N bond in **4-Bu*^t^*** (*d*(C23–N5) = 1.220(2) Å). The formation of these complexes represents a new entry into NHC-cyanoborane chemistry, species which are typically extremely challenging to access.^23^ In fact, cationic cyanoboranes had not been described previously, a further testament to the powerful synthetic utility of complex **1**.

## Conclusions

In summary, the striking reactivity of the hydroborylene ligand in the HB:→Ni^II^ complex **1** with one molar equiv. of isocyanides or CO has given access to a new hydride-bridged isomeric form in hydroborylene transition-metal chemistry in complexes **2**, as well as the hydride- and CO-bridged hydroboryl complex **3**. In addition, the unprecedented boraketiminium and cyanoborenium salts **4** and **5**, respectively, resulted from reaction of **1** with two molar equiv. of isocyanides in good yields. As such, this chemistry demonstrates the potential utility of the hydroborylene ligand in HB:→TM complexes for the realisation of new functional groups in boron chemistry.

## Conflicts of interest

There are no conflicts to declare.

## Supplementary Material

Supplementary information

Crystal structure data
