# Supplementary material for: Striking transformations of the hydroborylene ligand in a HB:→NiII complex with isocyanides and CO
Source: Chem Sci. 2018 Feb 5;9(9):2595–600. doi: 10.1039/c7sc04792d (PMC5897887; doi:10.1039/c7sc04792d)
Supplement: Supplementary file 1 [file SC-009-C7SC04792D-s001.pdf]

Electronic Supplementary Material (ESI) for Chemical Science.
This journal is © The Royal Society of Chemistry 2018

Striking Transformations of the Hydroborylene Ligand in a HB:→Ni^{II} Complex with Isocyanides and CO

Terrance J. Hadlington,[†] Tibor Szilvási,[‡] and Matthias Driess,[†,*]

[†]*Department of Chemistry, Metalorganics and Inorganic Materials, Technische Universität Berlin, Strasse des 17. Juni 135, Sekr. C2, 10623 Berlin, Germany*

[‡] *Department of Chemical & Biological Engineering, University of Wisconsin—Madison, 1415 Engineering Drive, 53706, Madison, WI, United States*

Contents:

1. **Experimental methods and data…………………2**
2. **X-Ray crystallographic data……………………..25**
3. **Computational methods and data……………….12**

1. Experimental methods and data

General considerations. All experiments and manipulations were carried out using standard Schlenk techniques or in an MBraun inert atmosphere glovebox under dry oxygen free nitrogen atmosphere. Hexane, diethylether, toluene and tetrahydrofuran (THF) were dried by standard methods. Benzene-d_6 and THF-d_8 were stirred over a sonicated potassium mirror for a period of 48 h and recondensed into a Schlenk tube containing activated 4 Å mol sieves. Dichloromethane (DCM)-d_2 was stirred over CaH_2 for 24 h and distilled into a Schlenk tube containing activated 4 Å molecular sieves. NMR spectra were recorded on a Bruker AV 200 or 400 Spectrometer. The ^{1}H and $^{13}C\{^{1}H\}$ NMR spectra were referenced to the residual solvent signals as internal standards. ^{11}B NMR spectra were externally calibrated with BF_3Et_2O. ESI mass spectra were recorded on an Orbitrap LTQ XL of Thermo Scientific mass spectrometer, and the raw data evaluated using the X-calibur computer program. Melting point samples were sealed in a glass capillary under argon. The starting materials NHC [:C{N(Pr^{i})C(Me)}$_2$],[1] [:SiCl(^{TMS}L)(NHC)],[2] and [cat(^{TMS}L)Si(Cl)Ni←:BH(NHC)$_2$] (**1**)[3] and were synthesized according to known literature procedures. All other reagents were used as received. In some cases, elemental analyses consistently gave low carbon readings (< -1.5%) most likely due to the formation of silicon carbides.[4]

[{cat(^{TMS}L)Si}(Cl)Ni(μ-CNCy)(μ-H)B(NHC)$_2$], 2-Cy. A solution of **1** (150 mg 0.18 mmol) in THF (15 mL) was cooled to -60 °C and neat CNCy (22 μL, 0,18 mmol) was added using a micro-pipette. The solution immediately became a deep yellow-orange. After stirring for 10 min, a ^{1}H and ^{11}B NMR spectroscopic analysis of the reaction mixture indicated that one clean product had formed. Concentration of the reaction mixture to 7 mL, and storage at -30 °C for 12 h resulted in the formation of large yellow-orange crystals (95 mg, 56 %). M.p.: 137-142 °C (dec.); ^{1}H NMR (d_8-THF, 400 MHz, 298 K): δ = 0.33 (s, 9H, N-Si*Me$_3$*), 1.10-1.40 (m, 10H, Cy-C*H$_2$*), 1.11 (d, $^{3}J_{HH}$ = 6.8 Hz, 6H, Dipp-Pr^{i}-C*H$_3$*), 1.23 (d, $^{3}J_{HH}$ = 6.8 Hz, 6H, Dipp-Pr^{i}-C*H$_3$*), 1.32 (br, 24H, NHC-Pr^{i}-C*H$_3$*), 2.26 (s, 12H, NHC-NC*Me*), 3.27 (m, 1H, Cy-C*H*), 4.06 (sept, $^{3}J_{HH}$ = 6.8 Hz, 2H, Dipp-Pr^{i}-C*H*), 5.48 (br, 4H, NHC-Pr^{i}-C*H*), 6.19 (m, 4H, cat-C*H*), 6.68 (m, 1H, *p*-Dipp-C*H*), 6.76 (m, 2H, *m*-Dipp-C*H*); ^{1}H NMR (d_2-DCM, 500 MHz, 263K): = 0.33 (s, 9H, N-Si*Me$_3$*), 1.15 (d, 6.8 Hz, 6H, Dipp-Pr^{i}-C*H$_3$*), 1.22 (d, 6.8 Hz, 6H, Dipp-Pr^{i}-C*H$_3$*), 1.00–1.60 (br, 34H, overlapping NHC-Pr^{i}-C*H$_3$* and Cy-CH$_2$), 2.23 (s, 12H, NHC-NC*Me*), 3.22 (m, 1H, Cy-C*H*), 3.98 (sept, $^{3}J_{HH}$ = 6.8 Hz, 2H, Dipp-Pr^{i}-C*H$_3$*), 4.89 (br, 2H, NHC-Pr^{i}-C*H*), 5.89 (br, 2H, NHC-Pr^{i}-C*H*), 6.37 (m, 4H, cat-C*H*), 6.89 (m, 3H, Dipp-C*H*); $^{13}C\{^{1}H\}$ NMR (d_2-DCM, 75.5 MHz, 213 K): 4.4 (N-Si*Me$_3$*), 10.6 (Dipp-Pr^{i}-*C*H$_3$), 19.7, 20.1, 22.5, and 24.4 (Cy-*C*H$_2$), 20.7, 21.3, 20.3 and 20.4 (NHC-Pr^{i}-*C*H$_3$), 25.2 and 25.4 (Dipp-Pr^{i}-*C*H), 25.8, 26.1, 27.4 and 27.8 (NHC-NC*Me*), 48.9, 50.3, 50.6 and 50.9 (NHC-Pr^{i}-*C*H), 71.3 (Cy-*C*H), 110.0 and 110.7 (NHC-N*C*Me), 117.0, 117.2, 122.7, 123.0, 125.3, 125.6, 126.4, 141.0, 146.5, 148.4, 151.0, and 152.3 (Ar-*C*); ^{11}B NMR (d_8-THF, 128 MHz, 298 K): -43.2 (br, :*B*-H); ^{11}B NMR (d_2-DCM, 128 MHz, 213 K): -43.8; IR, ν/cm^{-1} (ATR): 1626 (C=N); MS/ESI m/z (%): 181 ([NHC]$^{+}$, 100%); anal. calcd. for $C_{50}H_{82}BClN_6NiO_2Si_2$: C, 62.53 %; H, 8.61 %; N, 8.75 %; found: C, 58.99 %; H, 8.68 %; N, 8.21 %.

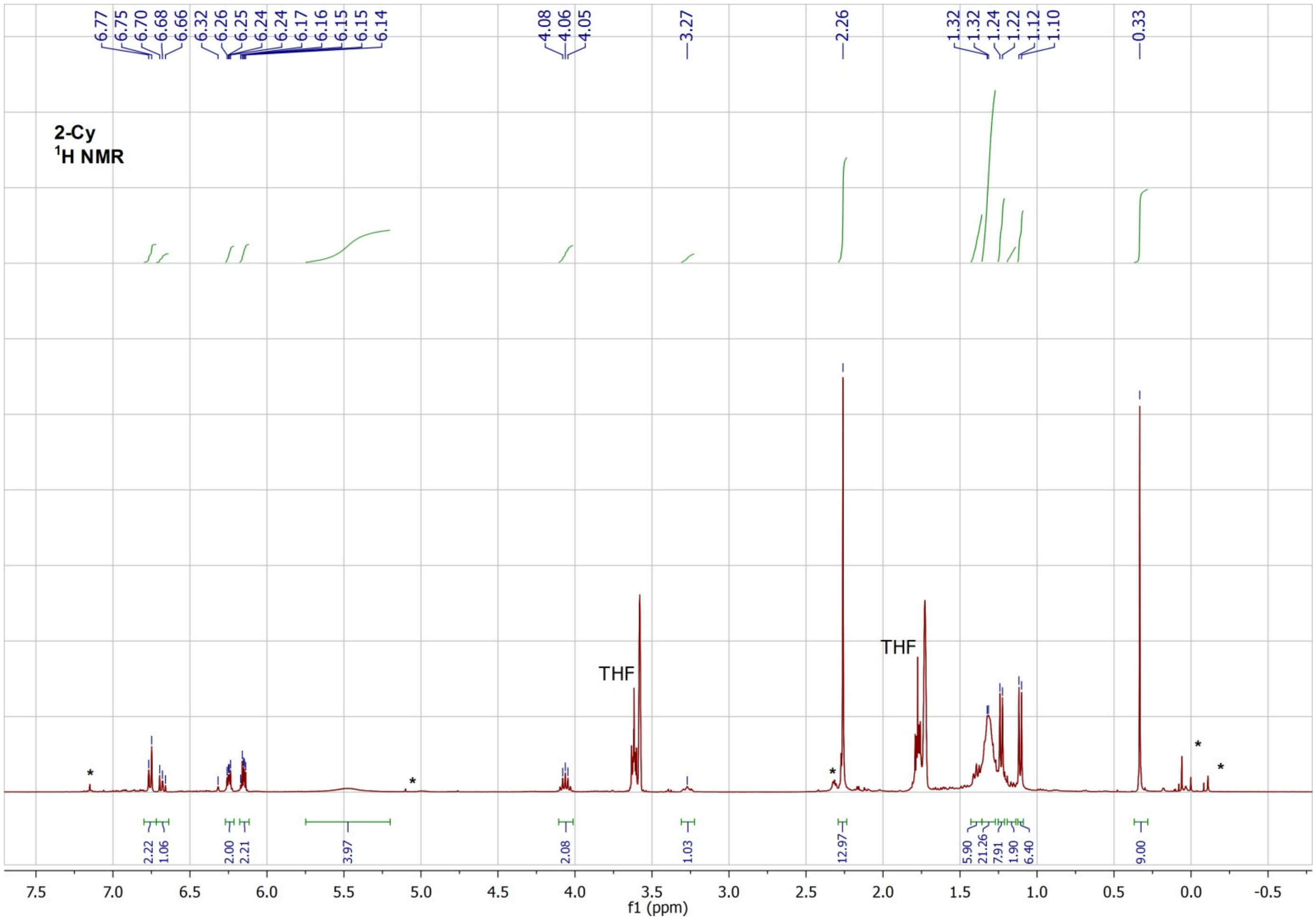

Figure S1. ^{1}H NMR spectrum of **2-Cy** in d_8-THF at ambient temperature. Impurities, labelled with asteriks, are due to the instability of **2-Cy** in solution at ambient temperature.

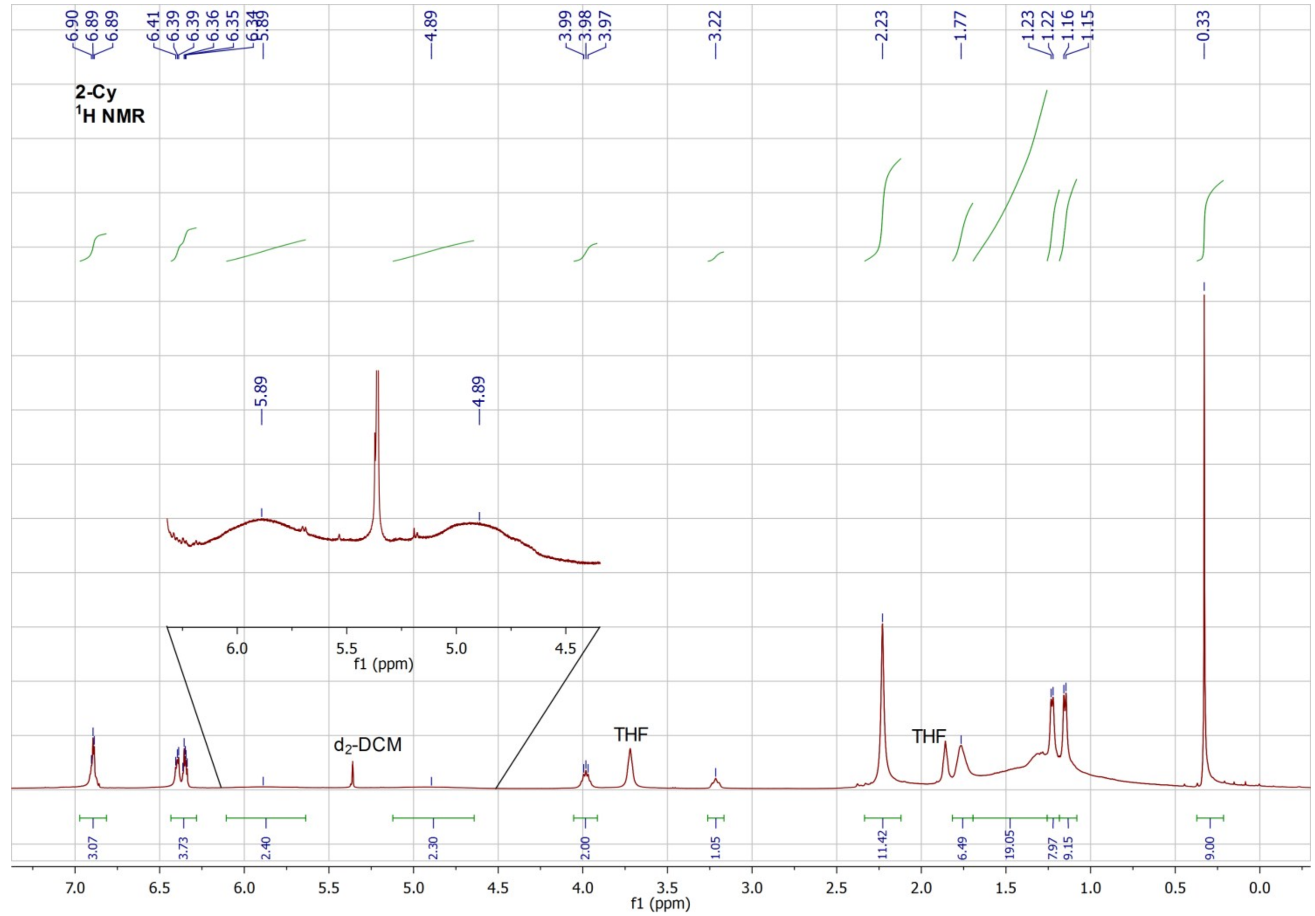

Figure S2. ^{1}H NMR spectrum of **2-Cy** in d_2-DCM at -10 °C.

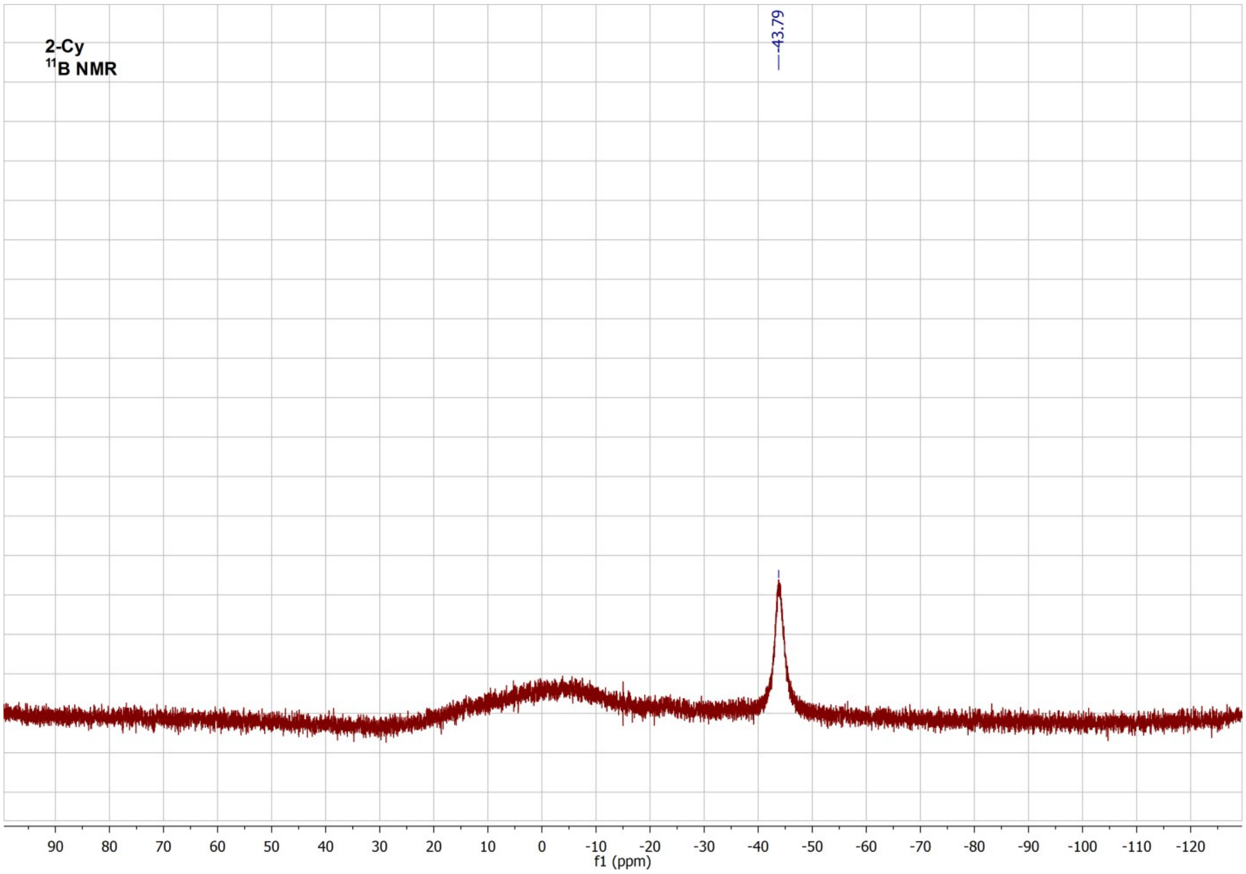

Figure S3 ^{11}B NMR spectrum of **2-Cy** in d_2-DCM at -10 °C.

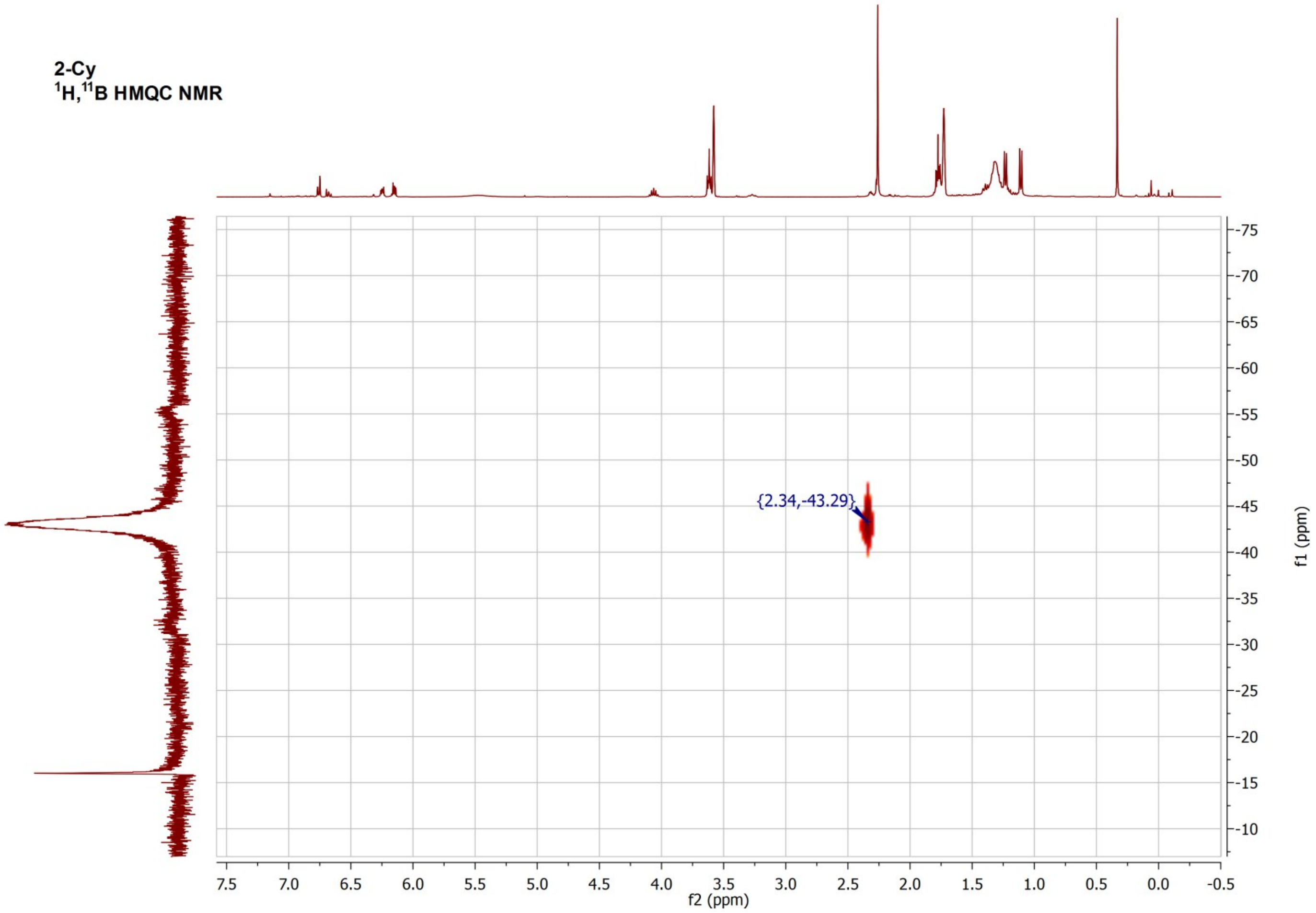

Figure S4. $^{1}H,^{11}B$ HMQC NMR spectrum of **2-Cy** in d_8-THF.

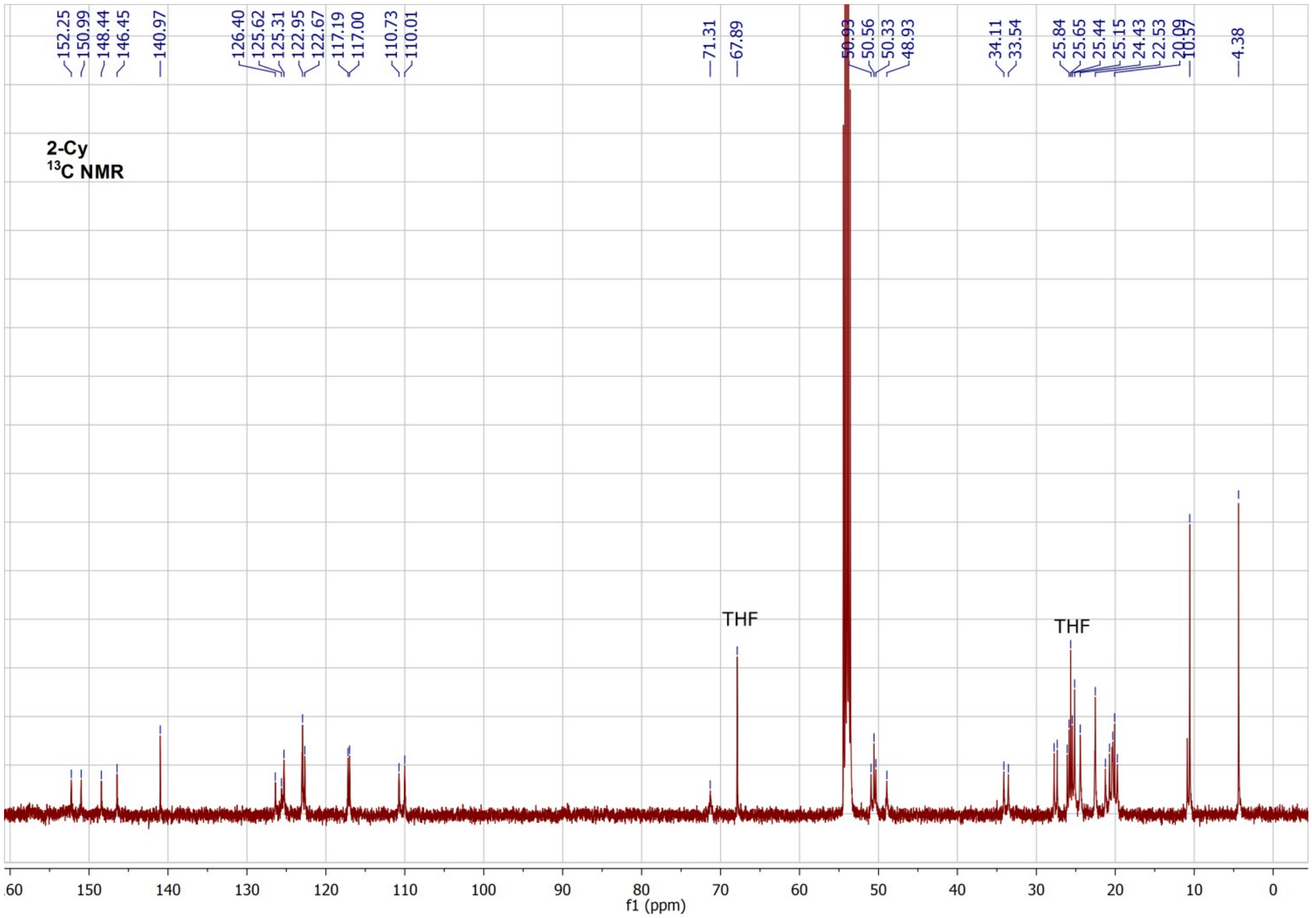

Figure S5. ^{13}C NMR spectrum of **2-Cy** in d_2-DCM at -60 °C. Note, collection of meaningful ^{13}C NMR spectra at higher temperatures was thwarted by severe broadening of signals at those temperatures.

[{cat(^{TMS}L)Si}(Cl)Ni)}(μ-CNBz)(μ-H)B(NHC)$_2$], 2-Bz. The procedure for the synthesis for **2-Cy** was followed, using **1** (150 mg 0.18 mmol) and a toluene solution of CNBz (0.24 mL, 0.5 M). The work-up involved concentration of the reaction mixture to 4 mL, and storage at -30 ^{o}C for 18 h, which yielded a large crop of X-ray quality yellow-orange crystals (90 mg, 53 %). M.p.: 125-130 ^{o}C (dec.); ^{1}H NMR (d_8-THF, 400 MHz, 298 K): δ = 0.30 (s, 9H, N-Si*Me$_3$*), 1.08 (d, $^{3}J_{HH}$ = 6.8 Hz, 6H, Dipp-Pr^{i}-C*H$_3$*), 1.25 (overlapping d, 30H, NHC-Pr^{i}-C*H*$_3$ and Dipp-Pr^{i}-C*H$_3$*), 2.24 (s, 12H, NHC-NC*Me*), 4.00 (sept, $^{3}J_{HH}$ = 6.8 Hz, 2H, Dipp-Pr^{i}-C*H*), 4.72 (s, 2H, Bz-C*H$_2$*), 5.50 (sept, $^{3}J_{HH}$ = 7.2 Hz, 4H, NHC-Pr^{i}-C*H*), 6.24 (m, 4H, cat-C*H*), 6.68 (m, 1H, *p*-Dipp-C*H*), 6.76 (m, 2H, *m*-Dipp-C*H*), 7.13 (m, 2H, Bz-C*H*), 7.21 (m, 2H, Bz-C*H*), 7.43 (m, 1H, Bz-C*H*); ^{1}H NMR (d_2-DCM, 500 MHz, 233 K): δ = 0.30 (s, 9H, N-Si*Me$_3$*), 1.11 (d, $^{3}J_{HH}$ = 6.8 Hz, 6H, Dipp-Pr^{i}-C*H$_3$*), 1.20 (d, $^{3}J_{HH}$ = 6.8 Hz, 6H, Dipp-Pr^{i}-C*H$_3$*), 0.90-1.50 (br, 24H, NHC-Pr^{i}-C*H$_3$*), 2.22 (s, 12H, NHC-NC*Me*), 3.57 (br, 1H, Dipp-Pr^{i}-C*H*), 3.89 (br, 1H, Dipp-Pr^{i}-C*H*), 4.80 (s, 2H, Bz-C*H$_2$*), 5.40 (br, 4H, NHC-Pr^{i}-C*H*), 6.36 (m, 4H, cat-C*H*), 6.90 (m, 3H, Dipp-C*H*), 7.23 (m, 1H, Bz-C*H*), 7.31 (m, 2H, Bz-C*H*), 7.51 (m, 2H, Bz-C*H*); ^{13}C{^{1}H} NMR (d_2-DCM, 75.5 MHz, 233 K): 4.1 (N-Si*Me$_3$*), 10.7 (Dipp-Pr^{i}-*C*H$_3$), 20.2 and 21.1 (br, NHC-Pr^{i}-*C*H$_3$), 25.2 (br, NHC-NC*Me*), 27.9 (br, Dipp-Pr^{i}-*C*H), 50.0 and 50.8 (br, NHC-Pr^{i}-*C*H), 67.8 (Bz-*C*H$_2$), 110.9 (NHC-

N*C*Me), 117.4, 123.1, 123.3, 126.0, 127.7, 129.1, 140.7, 142.5, 147.3, 147.9, 151.3 (Ar-*C*); ^{11}B NMR (d_8-THF, 128 MHz, 298 K): -43.7 (br, :*B*-H); ^{11}B NMR (d_2-DCM, 128 MHz, 298 K): -44.1; IR, ν/cm^{-1} (ATR): 1623 (C=N); MS/ESI m/z (%): 490 ([M-{LNiCl}]$^+$, 22%).

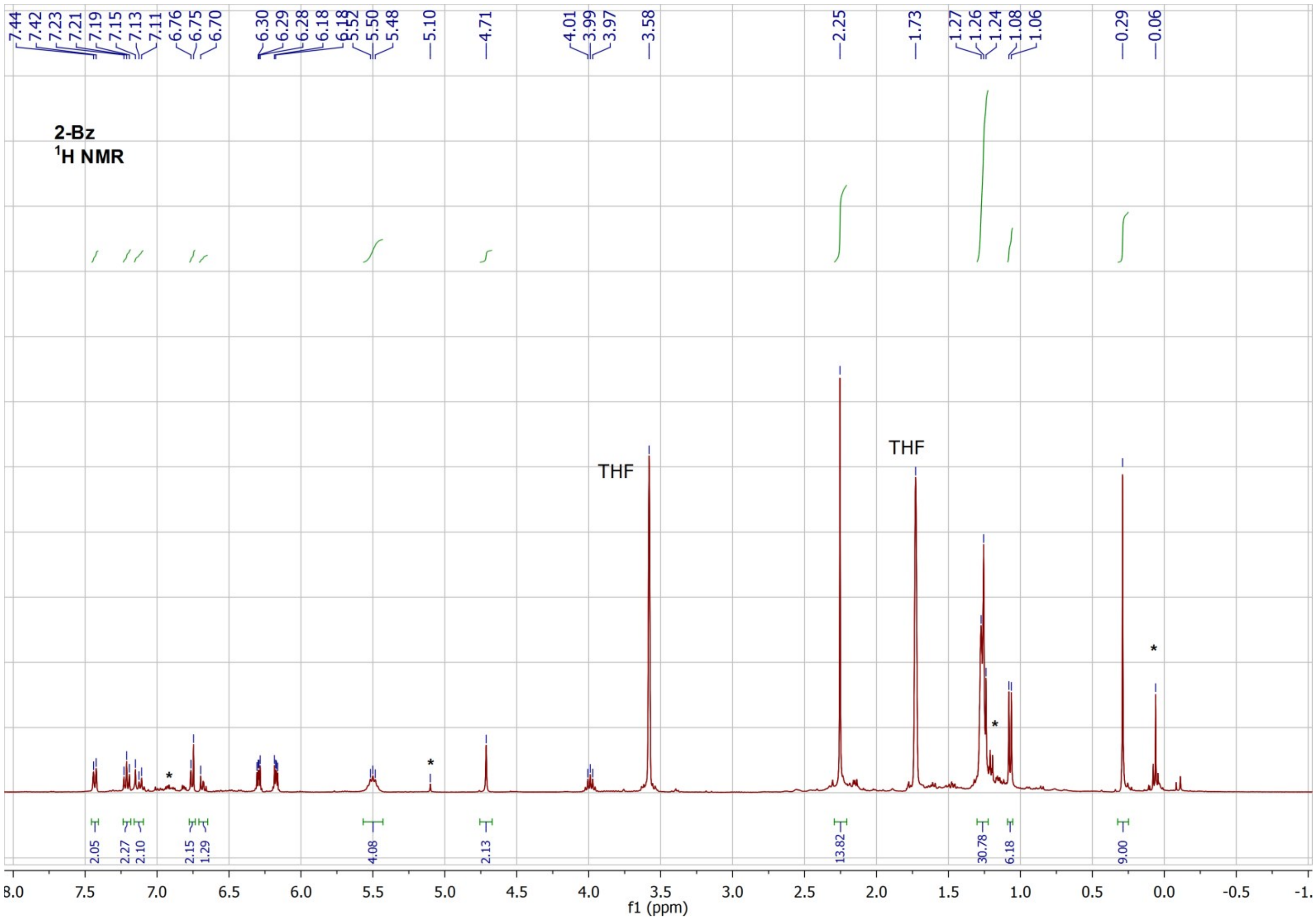

Figure S6. ^{1}H NMR spectrum of **2-Bz** in d_8-THF; * resonances from impurities due to unavoidable decomposition if **2-Bz** is dissolved at ambient temperature.

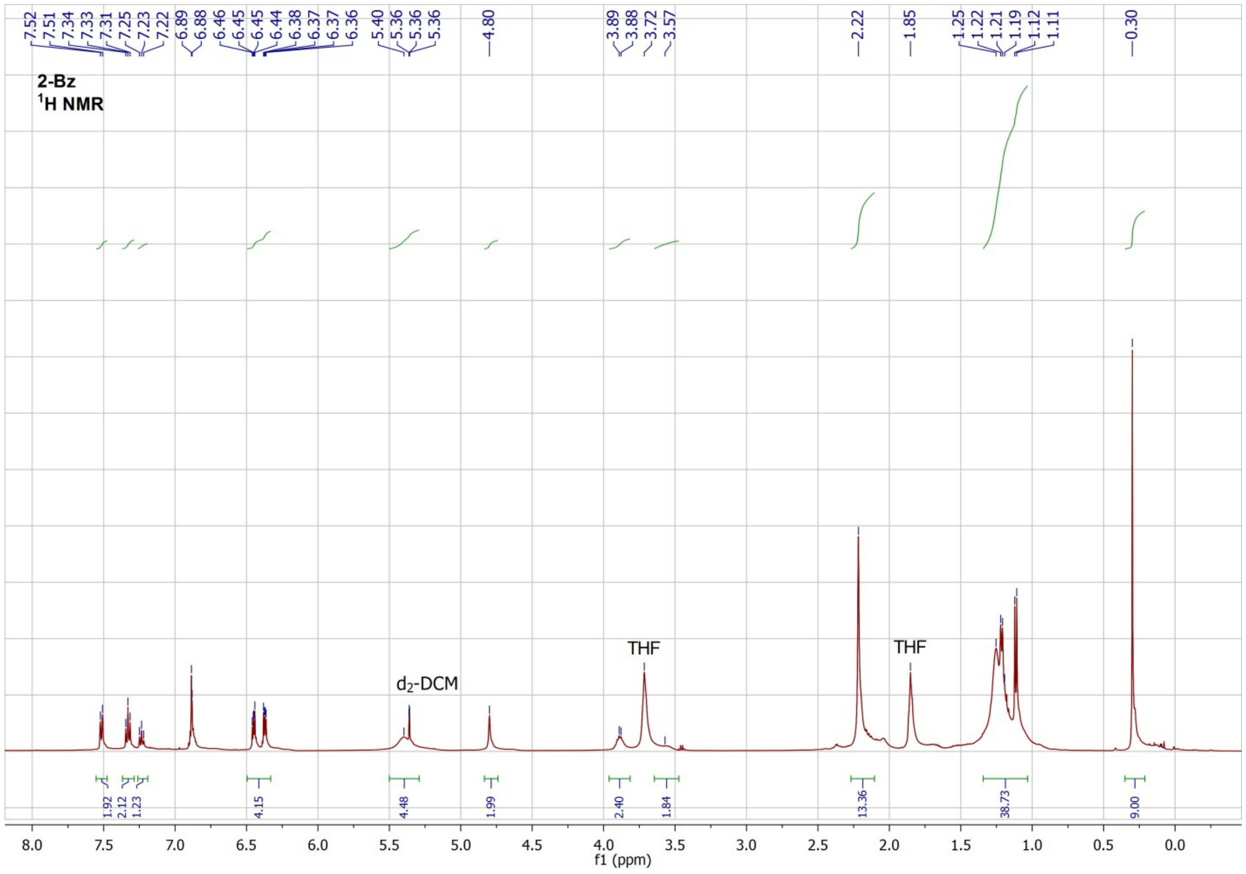

Figure S7. ^{1}H NMR spectrum of **2-Bz** in d$_2$-DCM at -40 °C.

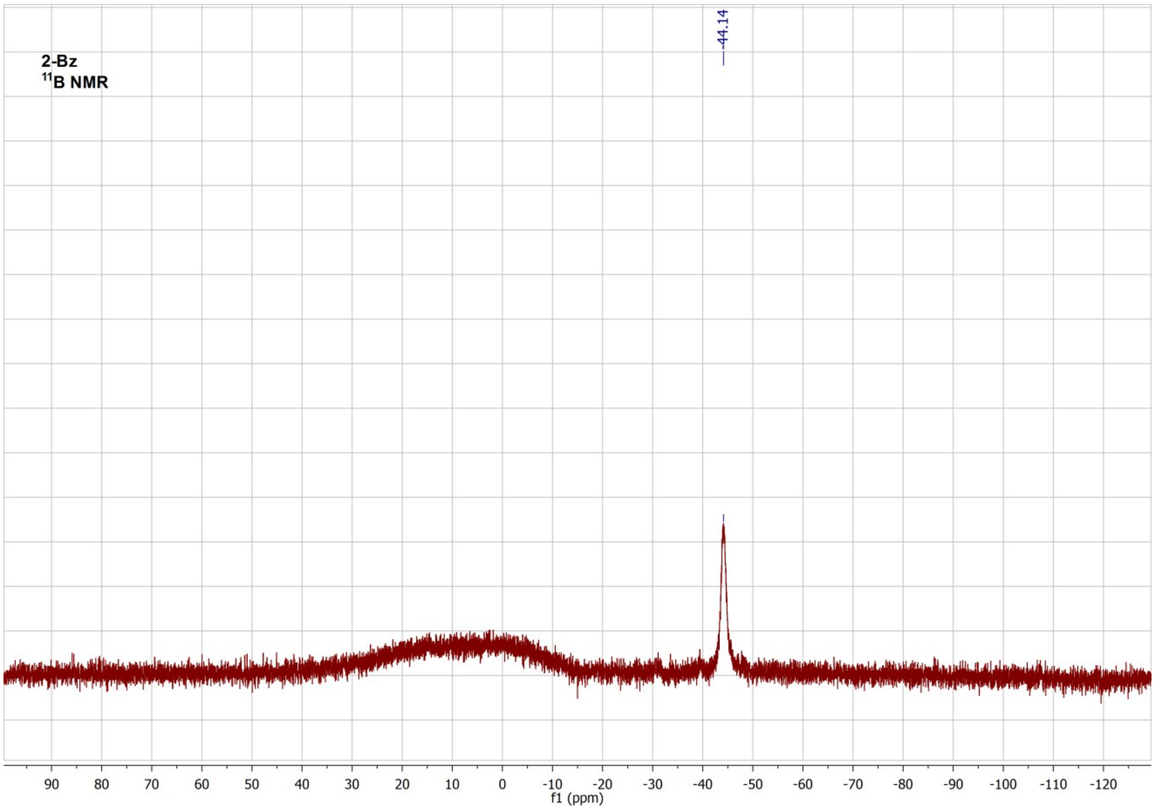

Figure S8. ^{11}B NMR spectrum of **2-Bz** in d_2-DCM at -40 °C.

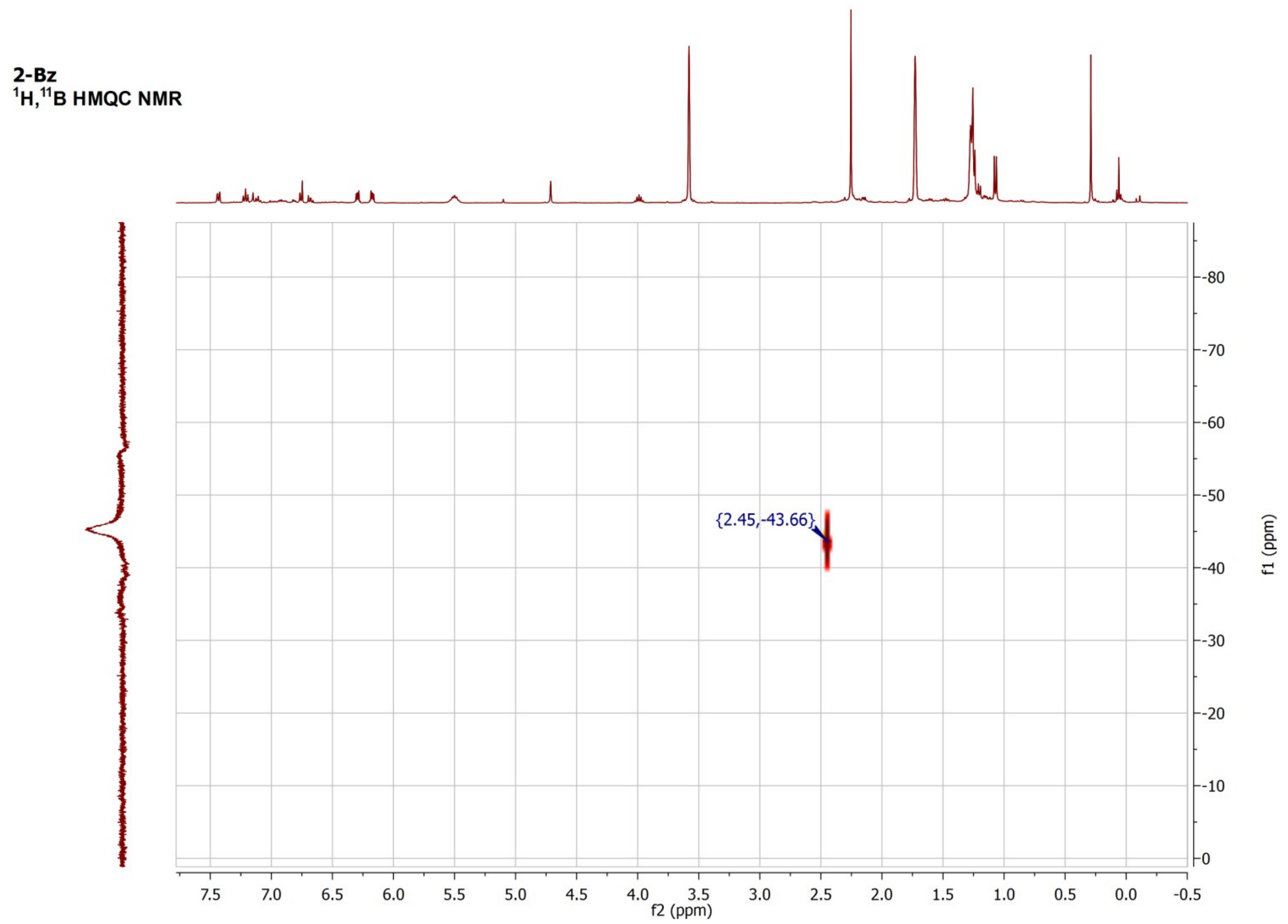

Figure S9. $^{1}H,^{11}B$ HMQC NMR spectrum of **2-Bz** in d_8-THF.

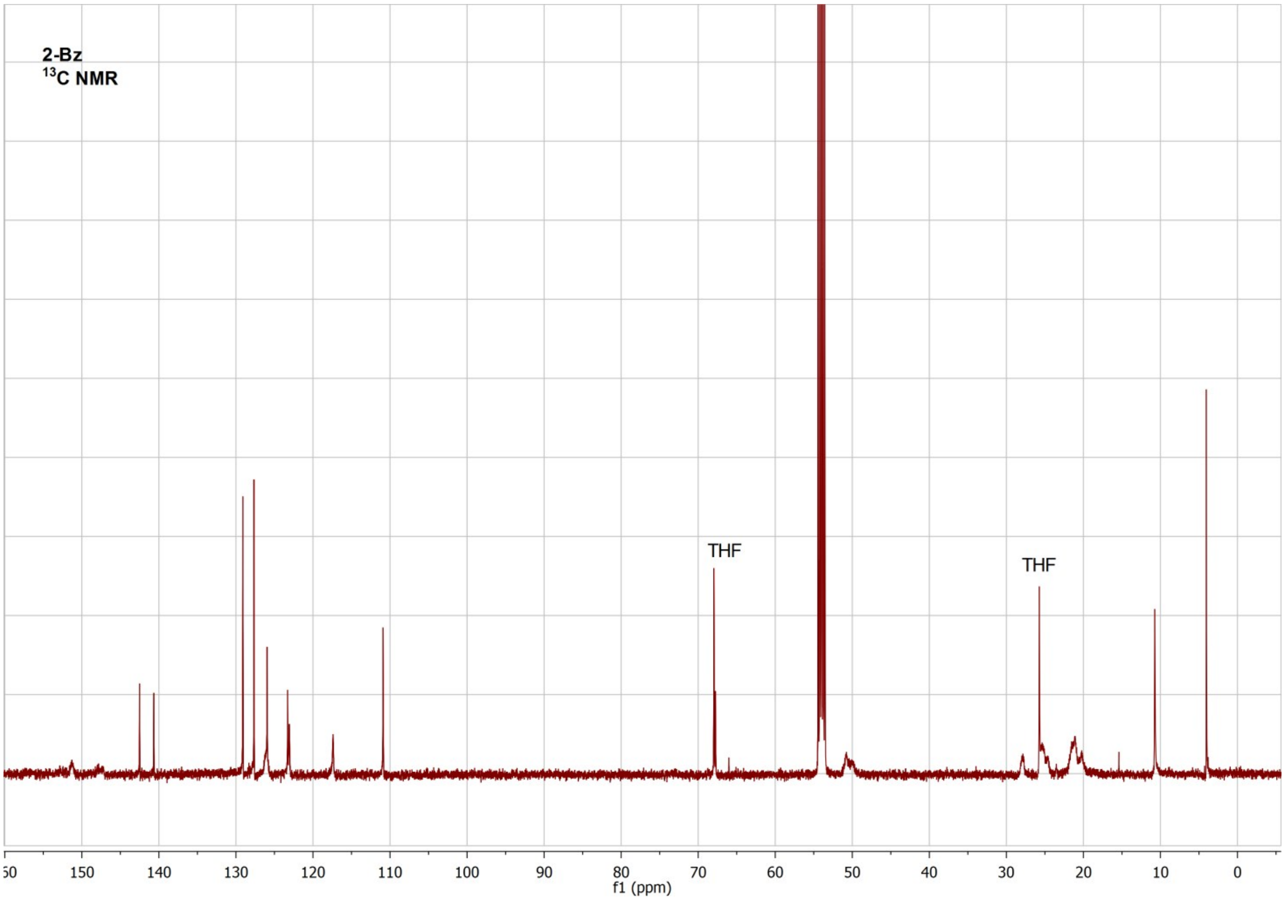

Figure S10. ^{13}C NMR spectrum of **2-Bz** in d_2-DCM at -40 °C.

[{cat(^{TMS}L)Si}(Cl)Ni)}(μ-CNXyl)(μ-H)B(NHC)$_2$], 2-Xyl. The procedure for the synthesis for **2-Cy** was followed, using **1** (100 mg 0.12 mmol) and CNXyl (16 mg, 0.12 mmol) dissolved in THF (2 mL). The work-up involved concentration of the reaction mixture to 2 mL, and layering with hexane (3 mL), which resulted in the formation of a large crop of X-ray quality deep red crystals of **2-Xyl** (65 mg, 56 %). M.p.: 148-155 ^{o}C (dec.); ^{1}H NMR (d_2-DCM, 400 MHz, 213 K): δ = -0.23 (s, 9H, N-Si*Me$_3$*), 0.68 (d, $^{3}J_{HH}$ = 6.8 Hz, 3H, Dipp-Pr^{i}-C*H*$_3$), 0.88 (d, $^{3}J_{HH}$ = 6.8 Hz, 3H, Dipp-Pr^{i}-C*H*$_3$), 0.91 (br, 6H, Dipp-Pr^{i}-C*H*$_3$), 1.00-1.25 (br, 9H, NHC- Pr^{i}-C*H*$_3$), 1.27 (d, $^{3}J_{HH}$ = 7.2 Hz, 3H, NHC- Pr^{i}-C*H*$_3$), 1.31 (d, $^{3}J_{HH}$ = 7.2 Hz, 3H, NHC- Pr^{i}-C*H*$_3$), 1.51 (d, $^{3}J_{HH}$ = 7.2 Hz, 3H, NHC- Pr^{i}-C*H*$_3$), 1.56 (br, 3H, NHC- Pr^{i}-C*H*$_3$), 1.64 (d, $^{3}J_{HH}$ = 7.2 Hz, 3H, NHC- Pr^{i}-C*H*$_3$), 1.83 (s, 3H, Xyl-C*H*$_3$), 2.07 (s, 3H, Xyl-C*H*$_3$), 2.22 (s, 6H, NHC-NC*Me*), 2.25 (s, 3H, NHC-NC*Me*), 3.41 (br, 2H, Dipp-Pr^{i}-C*H*$_3$), 4.67 (br, 1H, NHC-Pr^{i}-C*H*), 4.84 (br, 1H, NHC-Pr^{i}-C*H*), 5.61 (br, 1H, NHC-Pr^{i}-C*H*), 6.02 (br, 1H, NHC-Pr^{i}-C*H*), 6.34 (br m, 4H, cat-C*H*), 6.38 (br m, Xyl-C*H*), 6.83 (br m, 3H, Dipp-C*H*); the compound was found to be too unstable in solution for long enough periods to attain meaningful ^{13}C NMR spectra; ^{11}B NMR (d_2-DCM, 128 MHz, 213 K): -42.3 (br, *B*-H); IR, ν/cm^{-1} (ATR): 1634 (C=N); MS/ESI m/z (%): 502 ([M-{LNiCl}]$^{+}$, 100%).

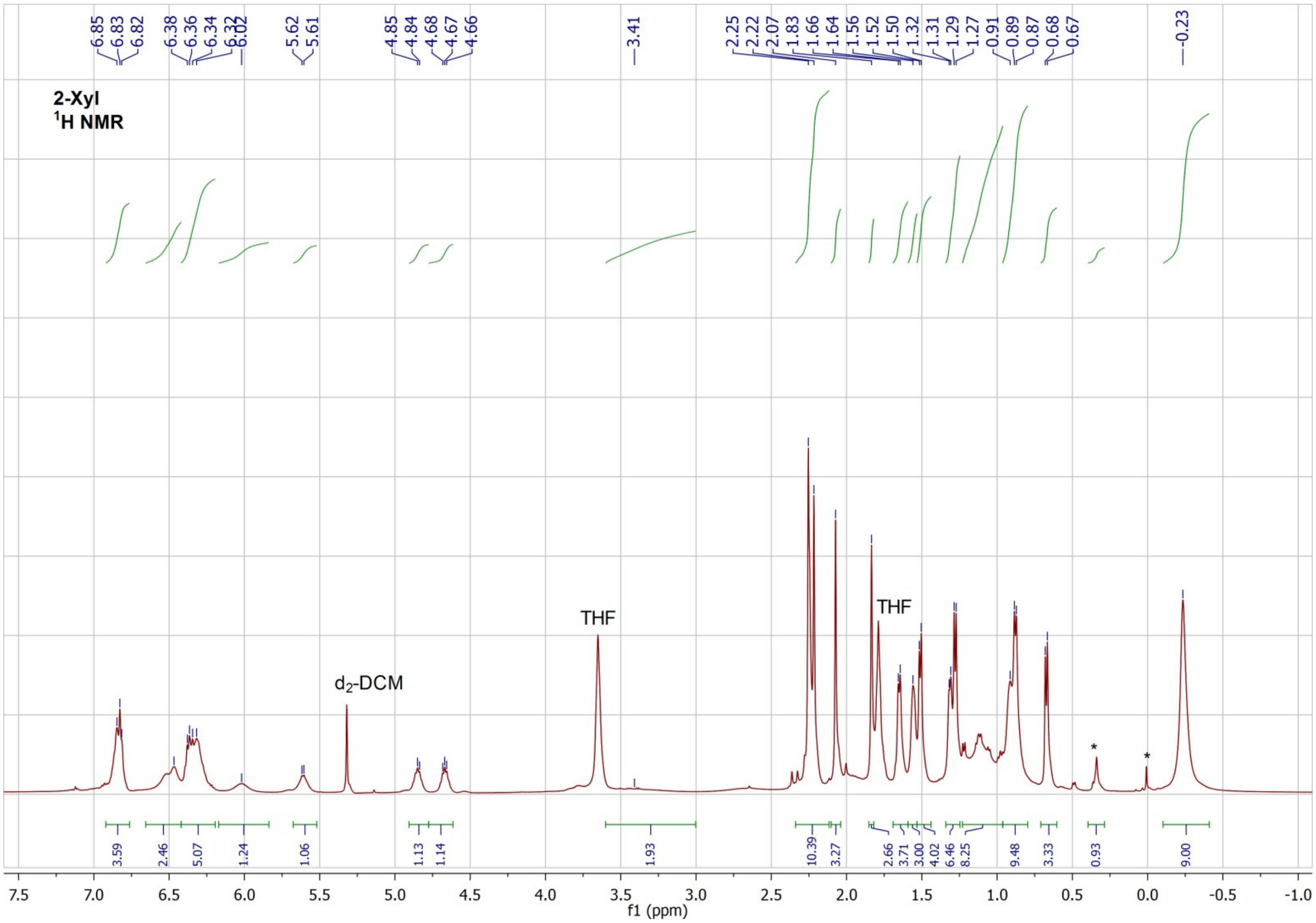

Figure S11. ^{1}H NMR spectrum of **2-Xyl** in d$_2$-DCM at 213K; * Impurities.

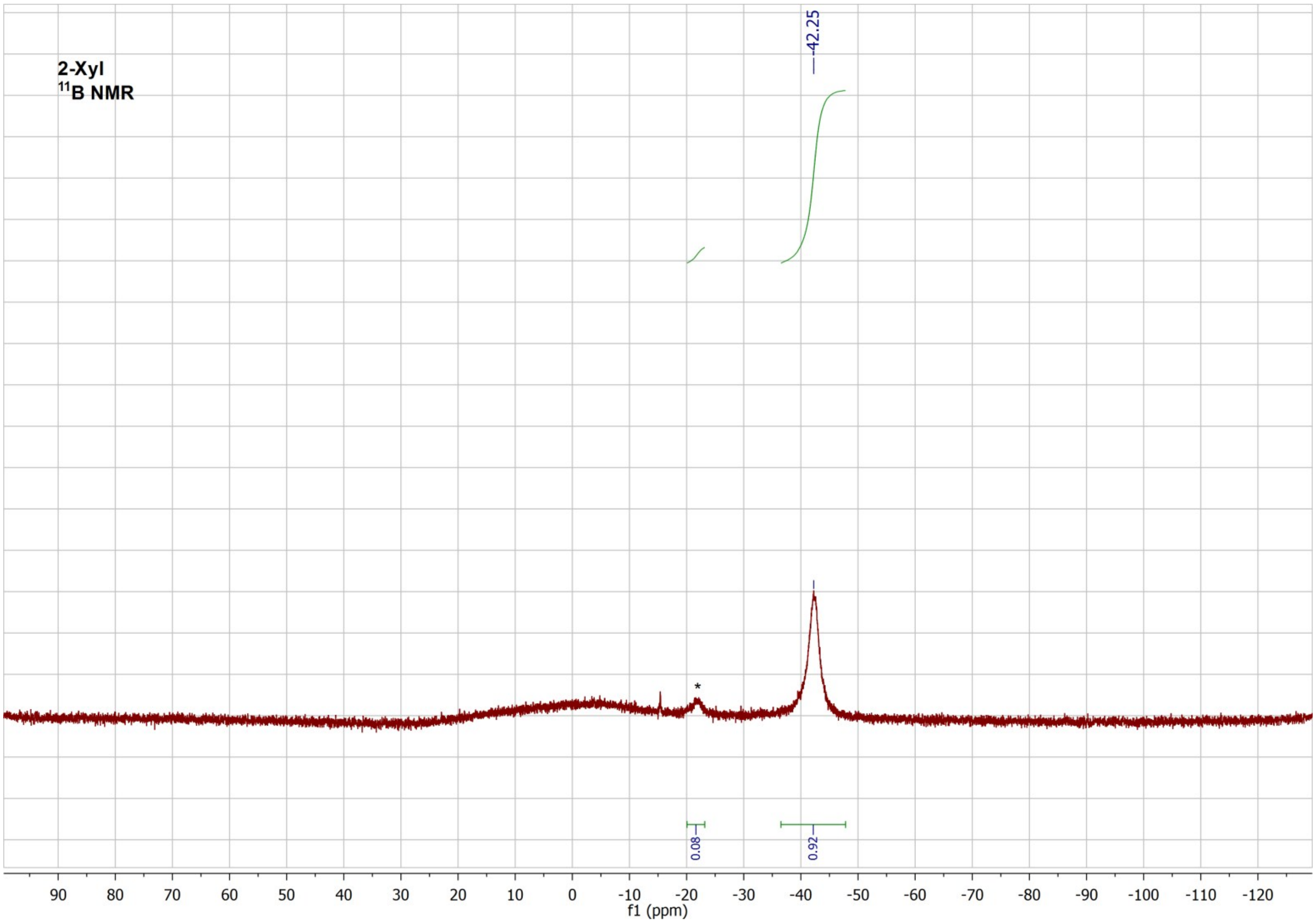

Figure S12. ^{11}B NMR spectrum of **2-Xyl** in d_2-DCM at 213K. * Unidentified impurity.

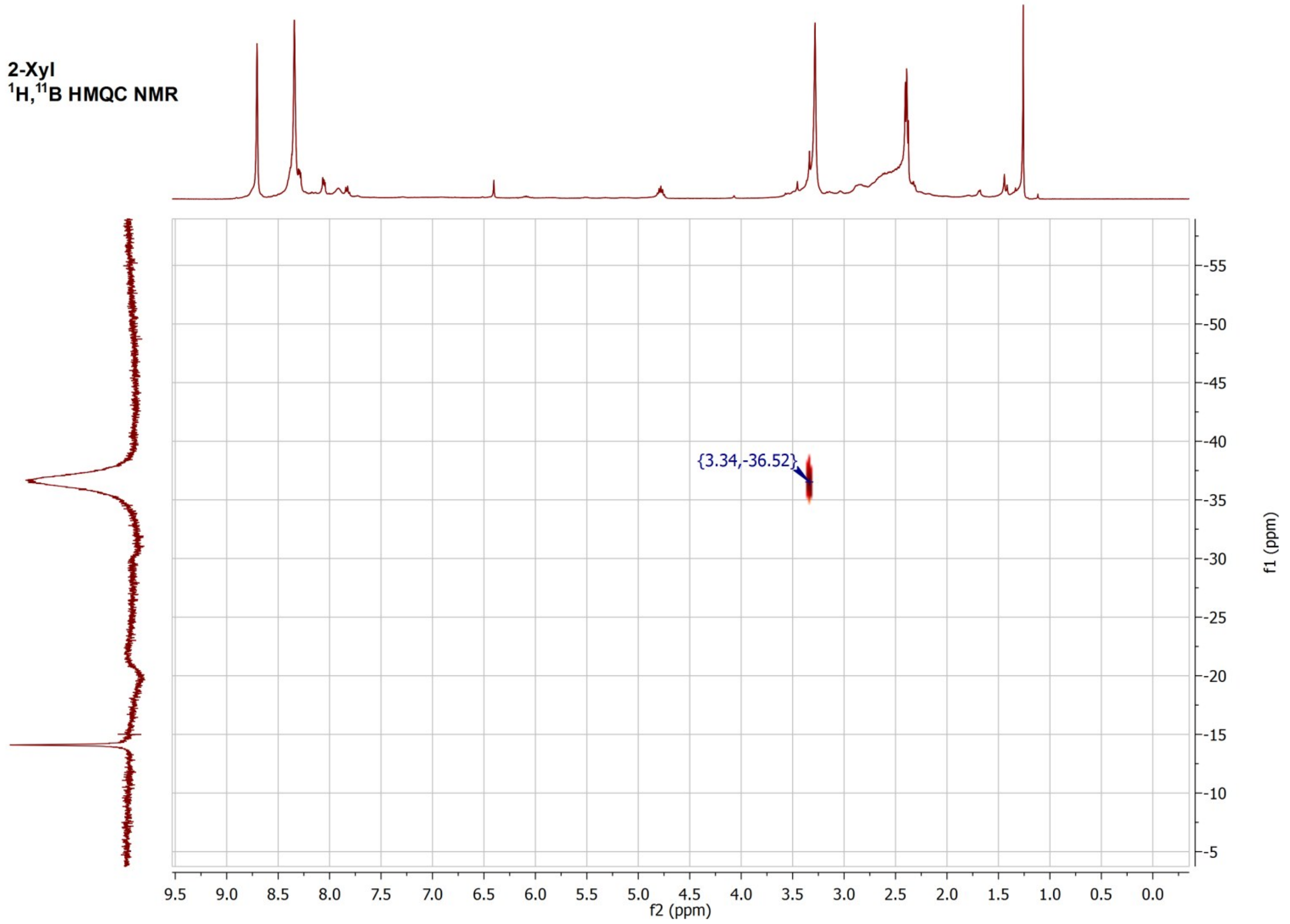

Figure S13. ^{1}H,^{11}B HMQC NMR spectrum of **2-Xyl** in d_5-pyridine.

[{cat(^{TMS}L)Si}(Cl)Ni(μ-CO)(μ-H)B(NHC)$_2$], 2-CO. A solution of **1** (100 mg, 0.12 mmol) dissolved in THF (2 mL) was cooled to -90 ^{o}C in a long thin Schlenk flask. CO gas (3.7 mL, 1.3 equiv.) was carefully layered above the solution under a slow N_2 flow, and the flask carefully sealed. Upon stirring the reaction mixture for 15 min, the reaction had lost its deep blue color, and become yellow-brown. The stir bar was subsequently removed from the reaction flask, and 10 mL of pre-cooled hexane layered above the THF reaction mixture. Storage of the layered mixture over night at -30 ^{o}C led to the formation of a mixed crop of **1** and **2-CO**. Alternatively, pre-cooled hexane (5 mL) can be added to the stirring reaction mixture at -90 ^{o}C, resulting in the precipitation of analytically pure **2-CO**, which was isolated by filtration and dried under vacuum (75 mg, 72 %). N.B. Storage of a suspension of **2-CO** in THF at -30 ^{o}C overnight leads to formation of a pale blue solution and growth of deep blue crystals of **1**. M.p.: 86-95 ^{o}C (dec.); ^{1}H NMR (d_2-DCM, 500 MHz, 233 K): δ = 0.10 (s, 9H, N-Si*Me$_3$*), 1.09 (dd, 12H, Dipp-Pr^{i}-C*H$_3$*), 1.30 (dd, 24H, NHC-Pr^{i}-C*H$_3$*), 2.20 (s, 12H, NHC-NC*Me*), 3.76 (sept, $^{3}J_{HH}$ = 6.8 Hz, 1H, Dipp-Pr^{i}-C*H*), 5.06 (br, 4H, NHC-Pr^{i}-C*H*), 6.43 (m, 4H, cat-C*H*), 6.86 (s, 3H, Dipp-Ar-C*H*); ^{13}C{^{1}H} NMR (d_2-DCM, 75.5 MHz, 233 K): 3.1 (N-Si*Me*$_3$), 10.7 (Dipp-Pr^{i}-*C*H$_3$), 20.8 and

20.9 (NHC-Pr^i-CH_3), 24.8 and 25.0 (NHC-NC*Me*), 27.5 (Dipp-Pr^i-*C*H), 50.9 (NHC-Pr^i-*C*H), 111.1 (NHC-N*C*Me), 117.5, 122.8, 123.1, 126.3, 140.6, 147.1, 151.1 (Ar-*C*); ^{11}B NMR (d_2-DCM, 128 MHz, 233 K): -42.6 (br, :*B*-H); ^{11}B NMR (d_8-THF, 128 MHz, 298 K): -19.1 (br d, $^1J_{BH}$ = 75 Hz, *B*-H); IR, ν/cm^{-1} (ATR): 1694 (C=O); MS/ESI m/z (%): 502 ([M-{LNiCl}]$^+$, 100%); anal. calcd. for $C_{44}H_{71}BClN_5NiO_3Si_2$: C, 60.11 %; H, 8.14 %; N, 7.97 %; found: C, 59.70 %; H, 8.22 %; N, 7.99 %.

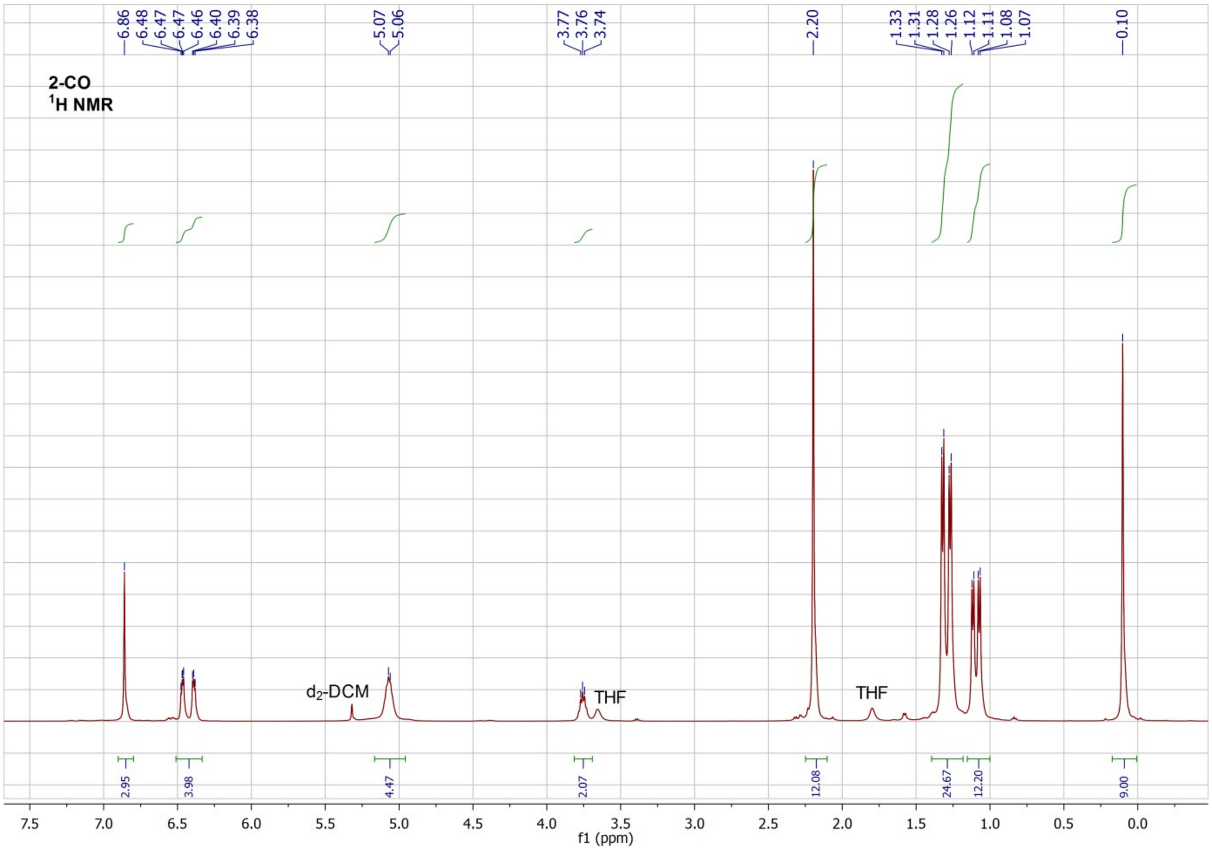

Figure S14. ^{1}H NMR spectrum of **2-CO** in d_2-DCM at -40 °C.

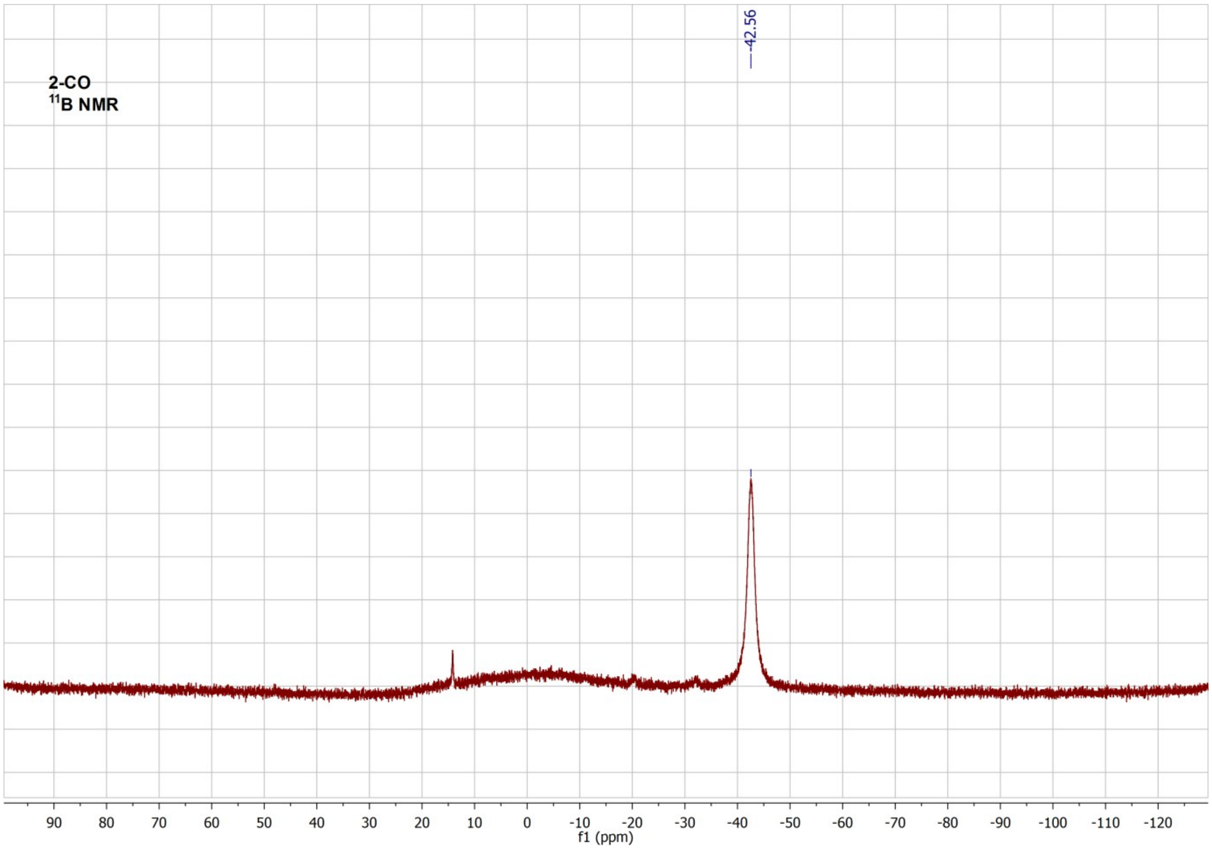

Figure S15. ^{11}B NMR spectrum of **2-CO** in d_2-DCM at -40 °C.

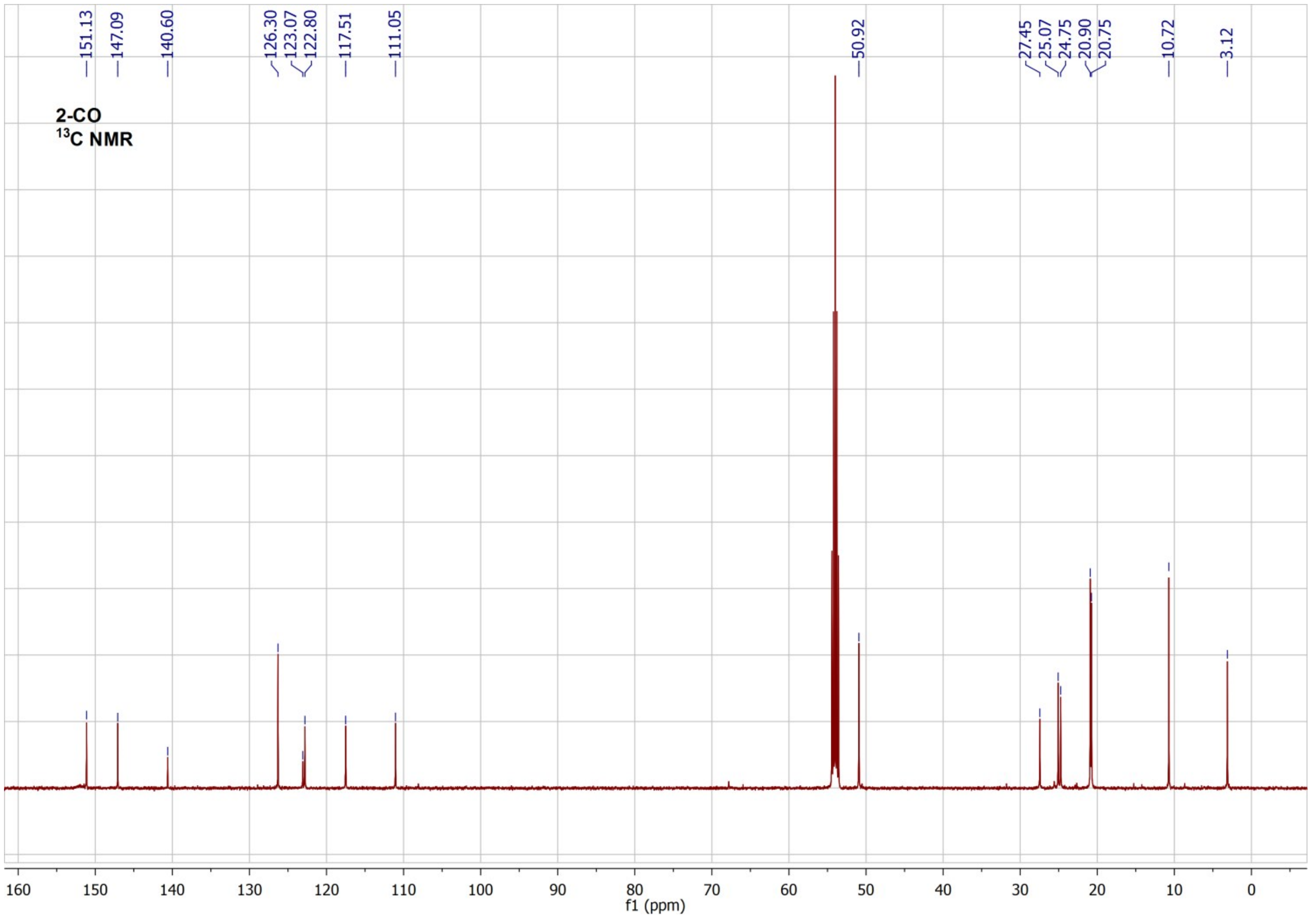

Figure S16. ^{13}C NMR spectrum of **2-CO** in d_2-DCM at -40 °C.

[(NHC)(Cl)Ni)}(μ-CO)(μ-H)B(NHC){cat(^{TMS}L)Si}], 3. A solution of **1** (100 mg, 0.12 mmol) dissolved in THF (2 mL) was cooled to -90 ^{o}C in a long thin Schlenk flask. CO gas (3.7 mL, 1.3 equiv.) was carefully layered above the solution, under a slow N_2 flow, and the flask carefully sealed. Upon stirring the reaction first became dark green, and then yellow-brown after 15 min. All volatiles were subsequently removed *in vacuo*, and the residue redissolved in diethyl ether, and filtered. Storage of the reaction mixture for 1 week at -30 ^{o}C led to the crystallisation of a mixture of **1** and **3**. Compound **3** was isolated by selective picking of crystals (20 mg, 20 %). M.p.: 161-170 ^{o}C (dec.); ^{1}H NMR (d_8-THF, 400 MHz, 298 K): δ = 0.04 (s, 9H, N-Si*Me₃*), 0.75 (d, $^3J_{HH}$ = 7.2 Hz, 3H, NHC-Pr^i-C*H*$_3$), 0.89 (d, $^3J_{HH}$ = 7.2 Hz, 3H, NHC-Pr^i-C*H*$_3$), 1.15 (d, $^3J_{HH}$ = 6.8 Hz, 6H, Dipp-Pr^i-C*H*$_3$), 1.18 (d, $^3J_{HH}$ = 6.8 Hz, 6H, Dipp-Pr^i-C*H*$_3$), 1.48 (br, 6H, NHC-Pr^i-C*H*$_3$), 1.52 (d, $^3J_{HH}$ = 7.2 Hz, 6H, NHC-Pr^i-C*H*$_3$), 1.55 (br, 6H, NHC-Pr^i-C*H*$_3$), 2.10 (s, 6H, NHC-NC*Me*), 2.23 (s, 6H, NHC-NC*Me*), 3.83 (sept, $^3J_{HH}$ = 6.8 Hz, 2H, Dipp-Pr^i-C*H*), 5.40 (br, 2H, NHC-Pr^i-C*H*), 5.50 (sept, $^3J_{HH}$ = 7.2 Hz, 2H, NHC- Pr^i-C*H*), 6.49 (m, 2H, cat-Ar-C*H*), 6.56 (m, 1H, cat-Ar-C*H*), 6.63 (m, 1H, cat-Ar-C*H*), 6.90 (m, 1H, Dipp-Ar-C*H*), 6.95 (m, 1H, Dipp-Ar-C*H*), 7.04 (m, 1H, Dipp-Ar-C*H*); ^{11}B NMR (d_8-THF, 128 MHz, 298 K): -57.8

(br d, $^{1}J_{BH}$ = 54 Hz, *B*-H); IR, ν/cm^{-1} (ATR): 1720 (C=O); MS/ESI m/z (%): 842 ([M-Cl]$^{+}$, 35%); anal. calcd. for $C_{44}H_{71}BClN_5NiO_3Si_2$: C, 60.11 %; H, 8.14 %; N, 7.97 %; found: C, 59.48 %; H, 8.41 %; N, 7.38 %.

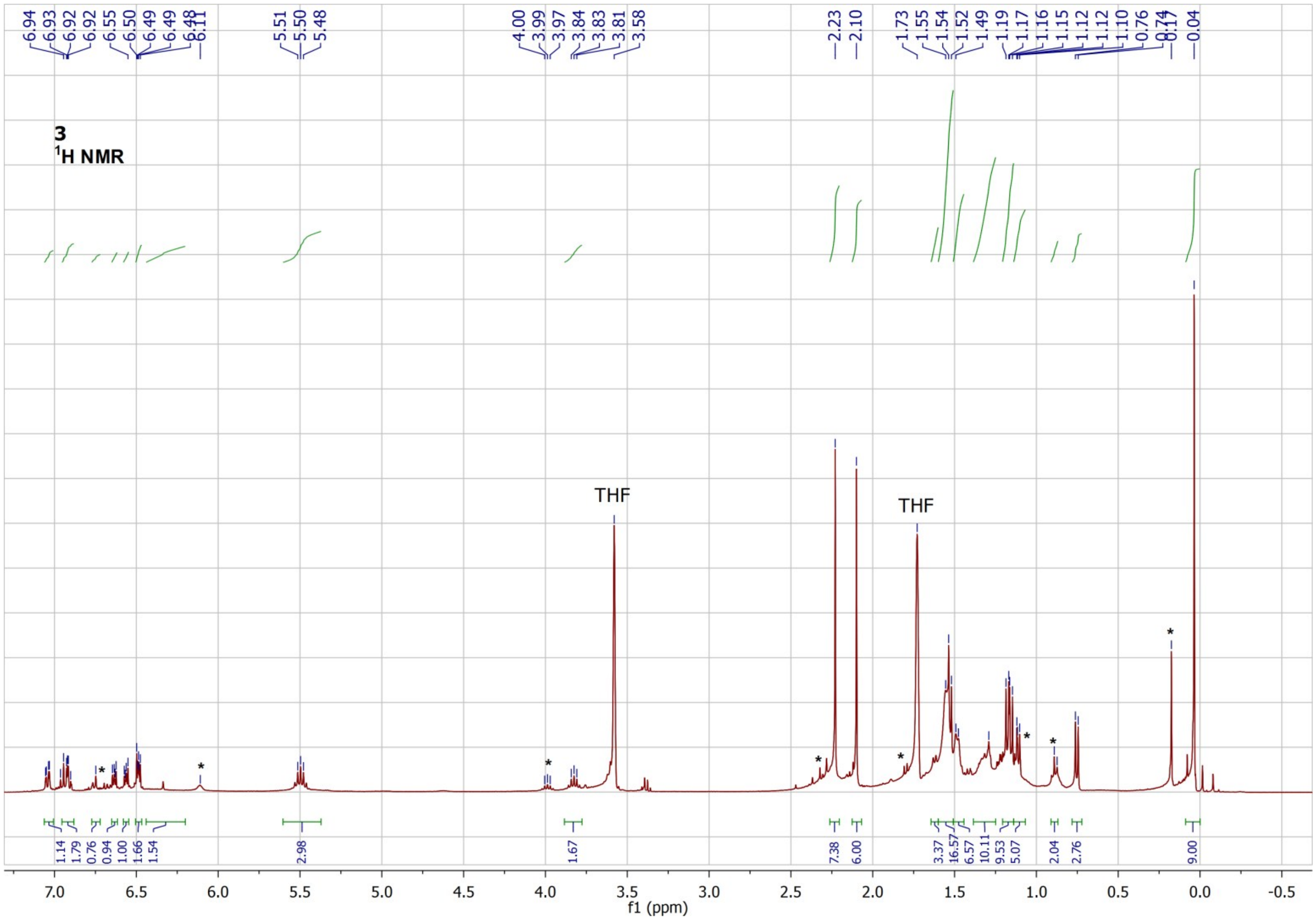

Figure S17. ^{1}H NMR spectrum of **3** in d_8-THF. * peaks from co-crystallised **1**.

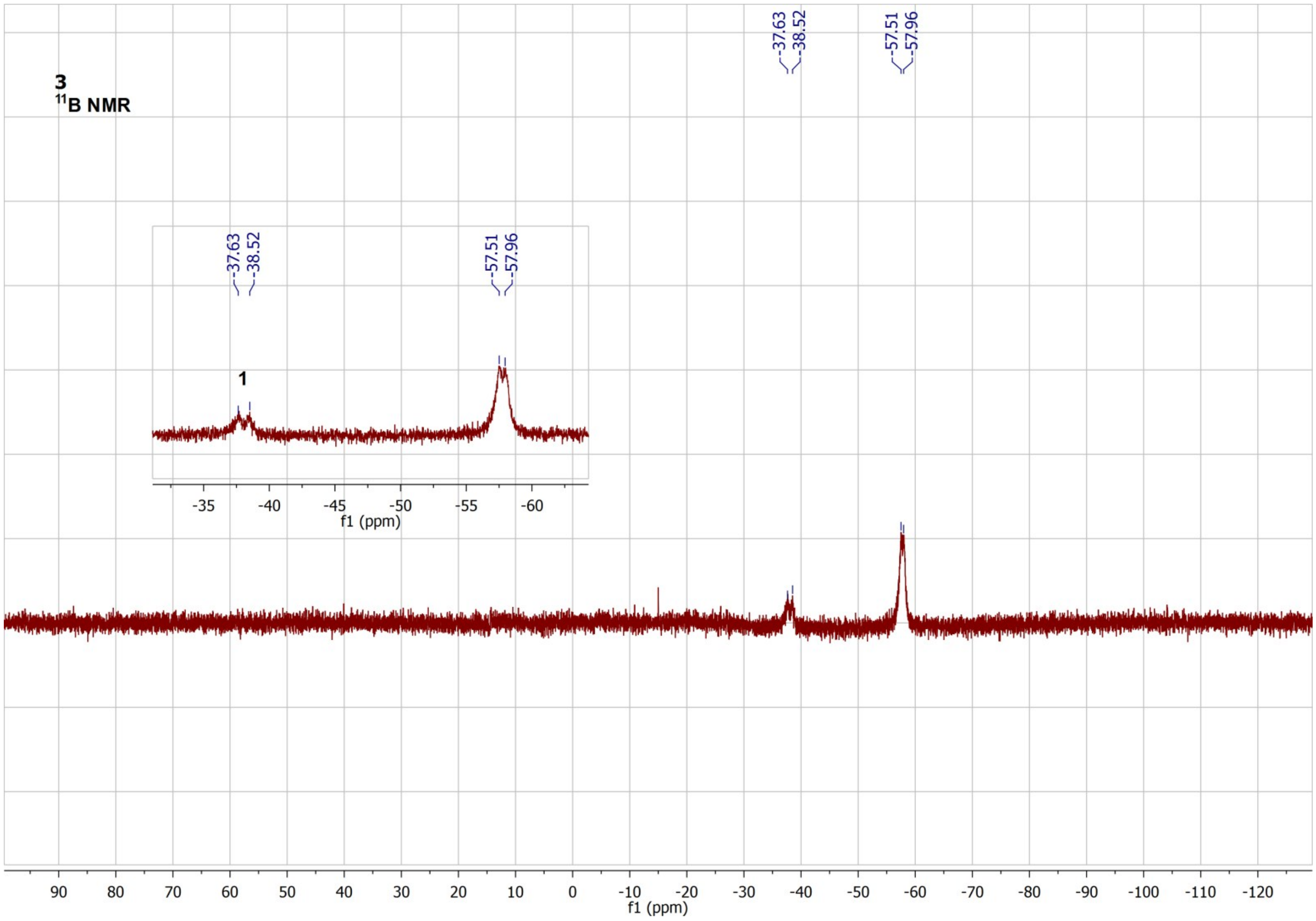

Figure S18. ^{11}B NMR spectrum of **3** in d_8-THF with co-crystallised **1**.

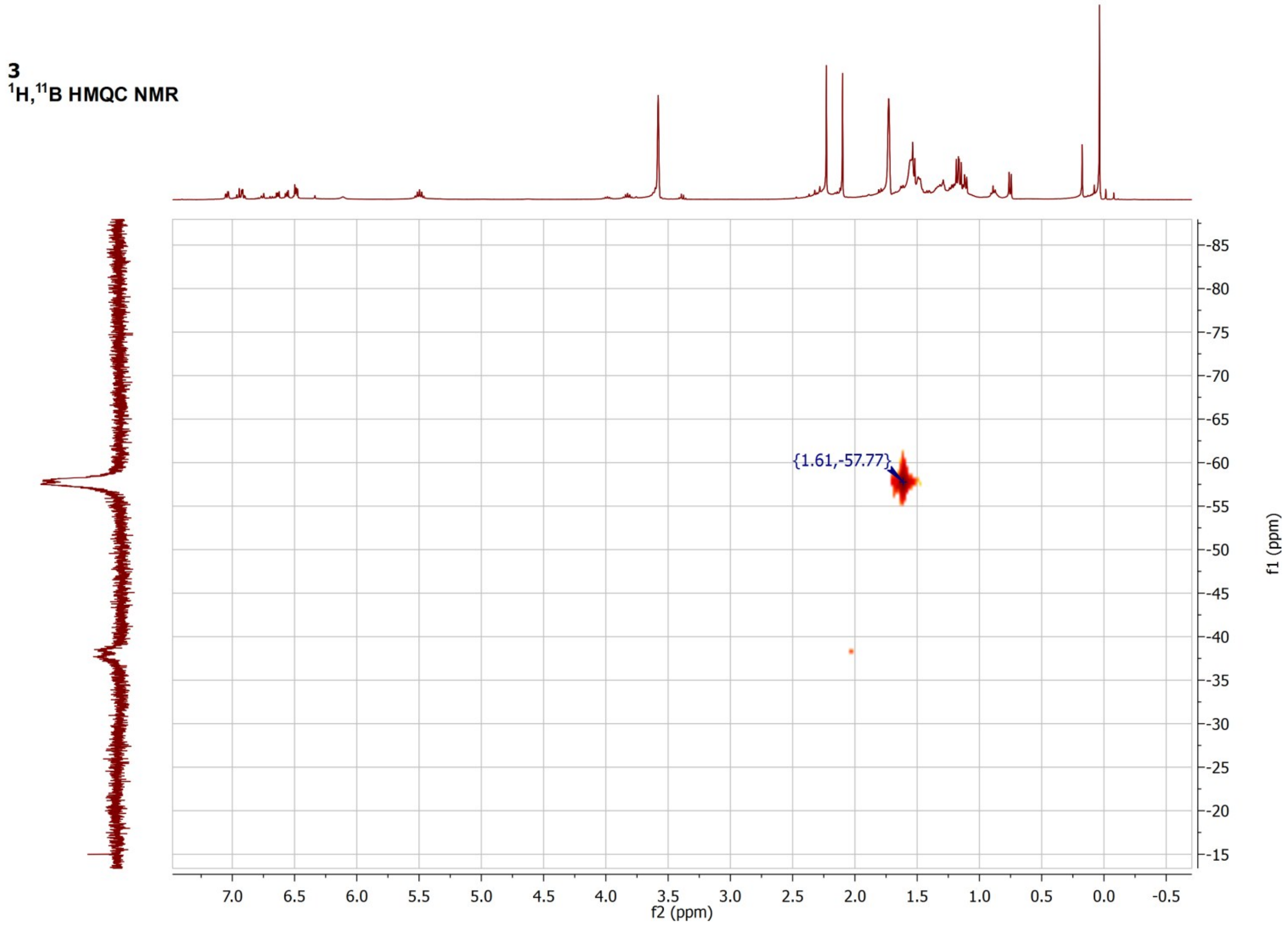

Figure S19. ^{1}H,^{11}B HMQC NMR spectrum of **3** in d_8-THF.

[(NHC)$_2$BCNCy]Cl, 4-Cy. A solution of **1** (150 mg 0.18 mmol) in THF (15 mL) was cooled to -80 ^{o}C and neat CNCy (44 µL, 0.36 mmol) was added using a micro-pipette. The solution immediately became deep yellow/brown in color. The reaction mixture was warmed to ambient temperature, at which stage an ^{11}B NMR spectroscopic analysis indicated that a single boron containing species had formed. Concentration of the reaction mixture to 3 mL, layering with 10 mL of hexane, and storage at 5 ^{o}C for 2 days resulted in the formation of yellow crystals and a brown oil. The crystals were isolated by washing with small amounts (1-5 mL) of THF, and were dried under vaccum (50 mg, 64 %). M.p.: 107-113 ^{o}C (melt); ^{1}H NMR (DCM-d_2, 400 MHz, 298 K): δ = 1.20-1.80 (m, 10H, Cy-C*H*$_2$), 1.39 (d, $^{3}J_{HH}$ = 7.2 Hz, 24H, NHC-Pr^{i}-C*H*$_3$), 2.31 (s, 12H, NHC-NC*Me*), 3.14 (m, 1H, Cy-C*H*), 4.95 (sept, $^{3}J_{HH}$ = 7.2 Hz, 4H, NHC-Pr^{i}-C*H*); $^{13}C\{^{1}H\}$ NMR (DCM-d_2, 75.5 MHz, 298 K): δ = 10.8 (Cy-*C*H$_2$),21.4 (NHC-Pr^{i}-*C*H$_3$), 21.6 (NHC-Pr^{i}-*C*H$_3$), 24.8 (Cy-*C*H), 34.3 (NHC-Pr^{i}-*C*H), 52.7 (NHC-NC*Me*), 118.2 (br, Cy-*C*-N), 127.4 (NHC-N<u>C</u>Me); ^{11}B NMR (DCM-d_2, 128 MHz, 298 K): -15.84 (s, *B*(CNCy)(NHC)$_2$); IR, ν/cm^{-1} (ATR): 1922 (C=N); MS/ESI m/z (%): 482 ([M-Cl]$^{+}$, 63%); anal. calcd. for $C_{33}H_{59}BClN_5O$: C, 67.40 %; H, 10.11 %; N, 11.91 %; found: C, 66.59 %; H, 10.30 %; N, 11.81 %.

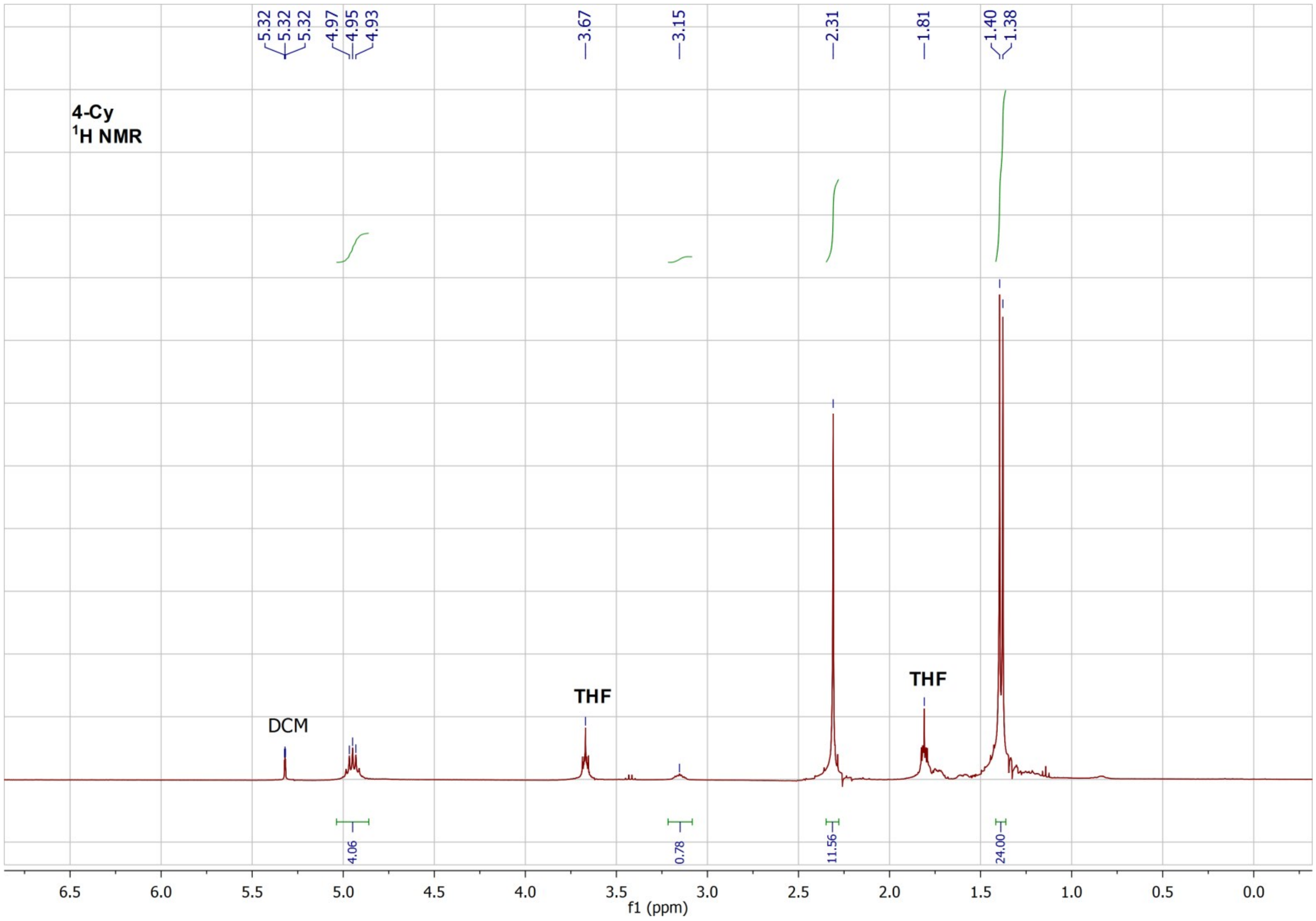

Figure S20. ^{1}H NMR spectrum of **4-Cy** in d_2-DCM.

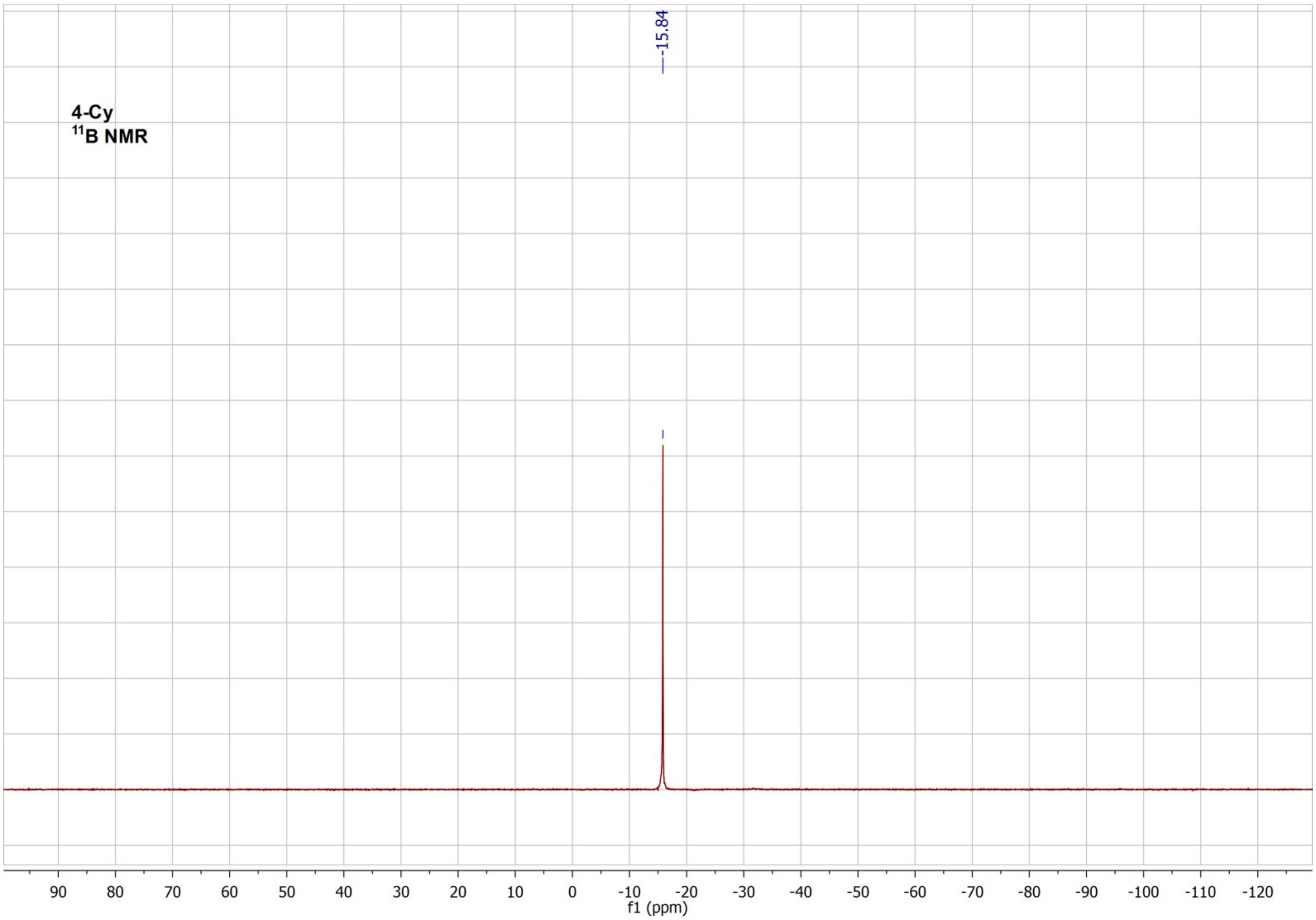

Figure S21. ^{11}B NMR spectrum of **4-Cy** in d_2-DCM.

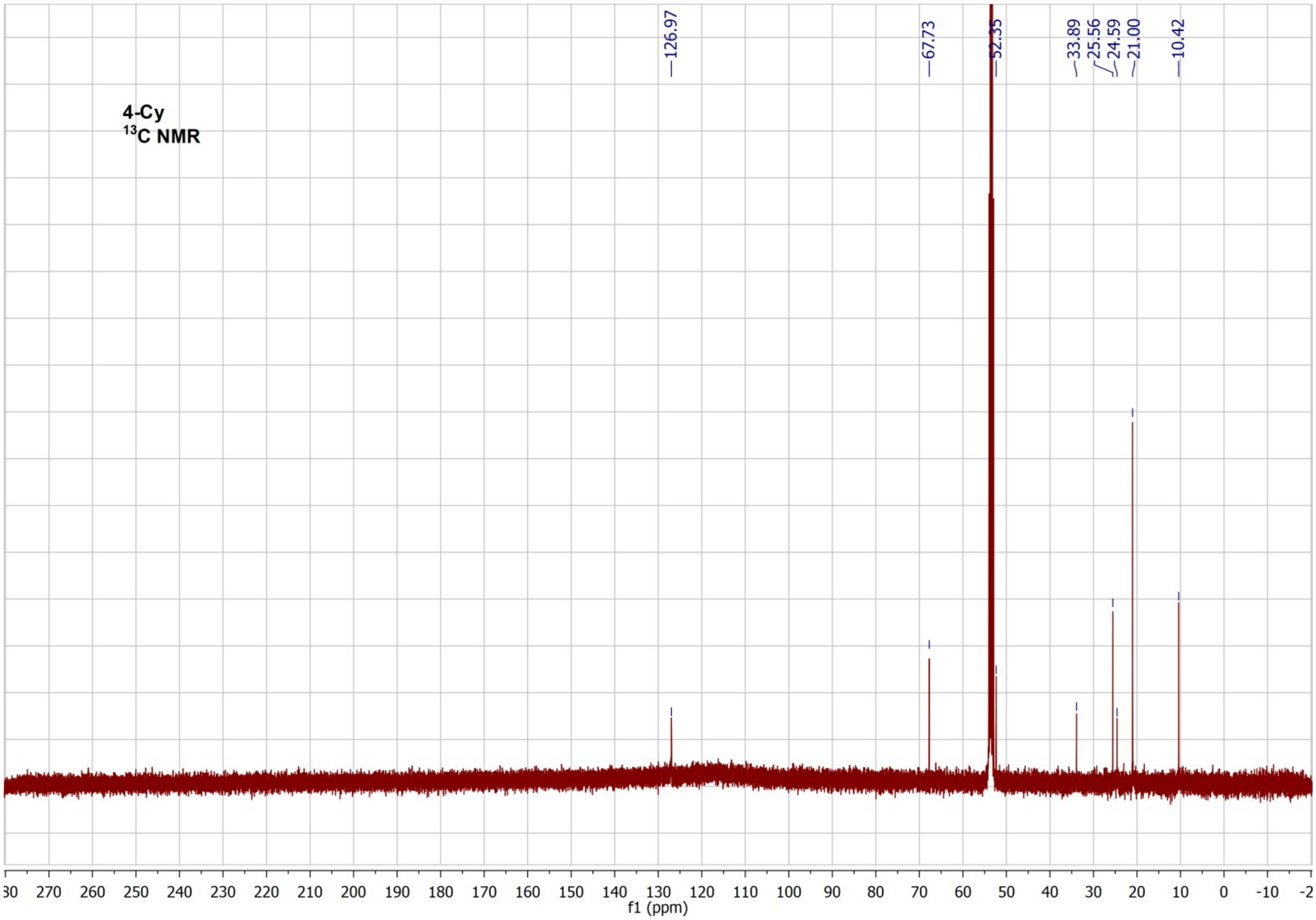

Figure S22. ^{13}C NMR spectrum of **4-Cy** in d_2-DCM.

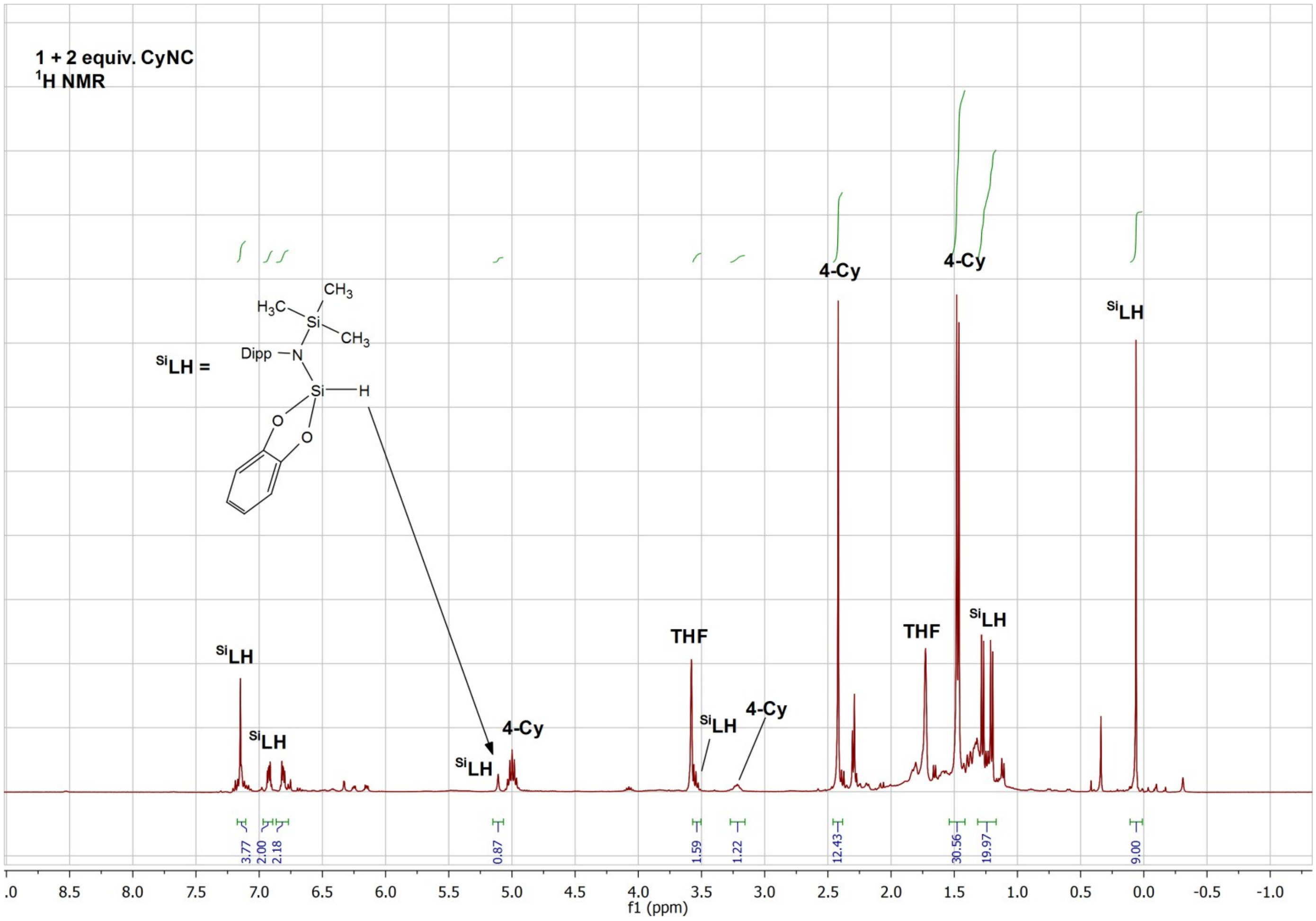

Figure S23. ^{1}H NMR of a 1:2 mixture of **1** and CyNC in d_8-THF after 15 min. ^{Si}LH denotes peaks relating to 'free' silane.

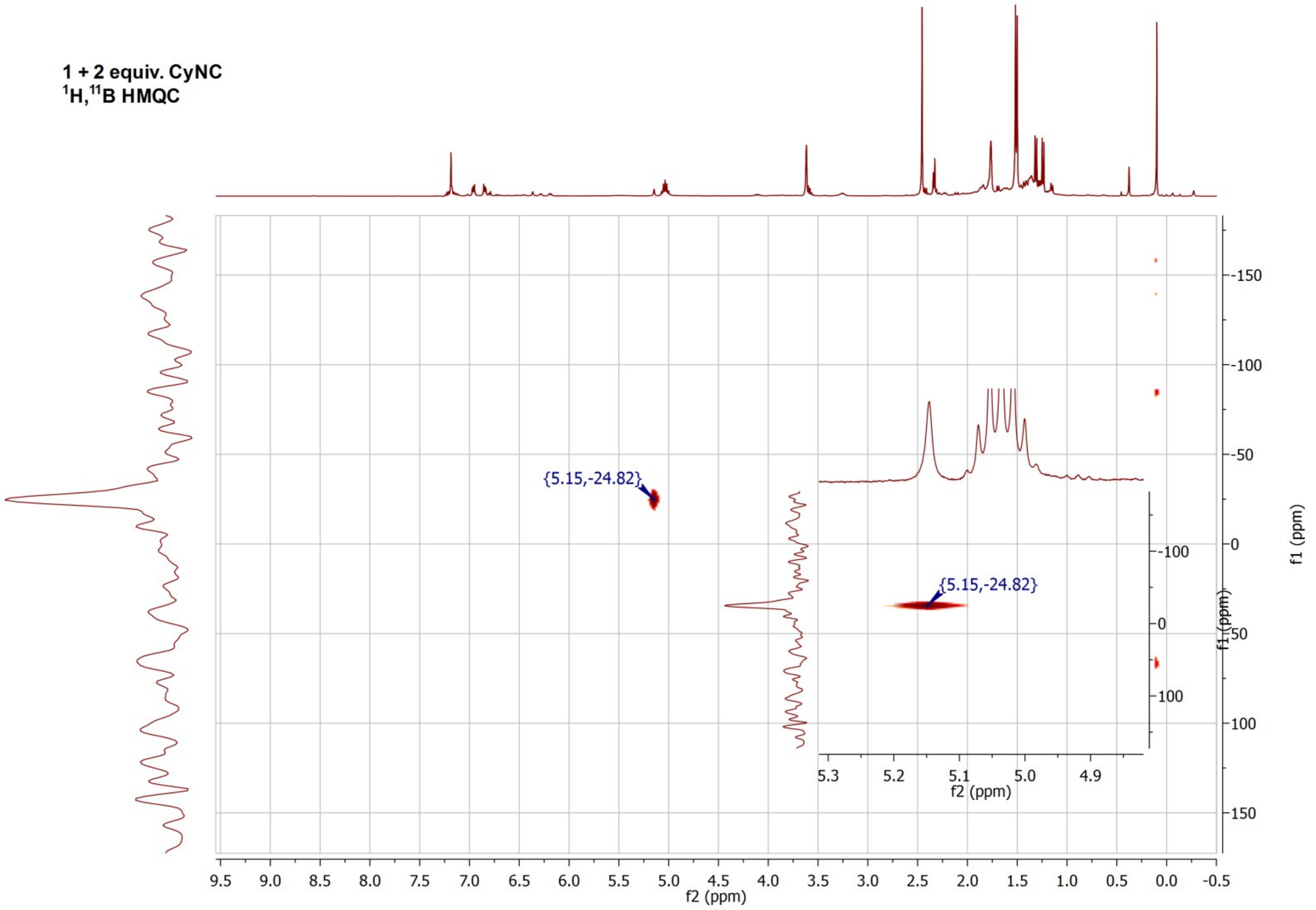

Figure S24. $^{1}H,^{29}Si$ HMQC NMR of a 1:2 mixture of **1** and CyNC in d_8-THF after 15 min.

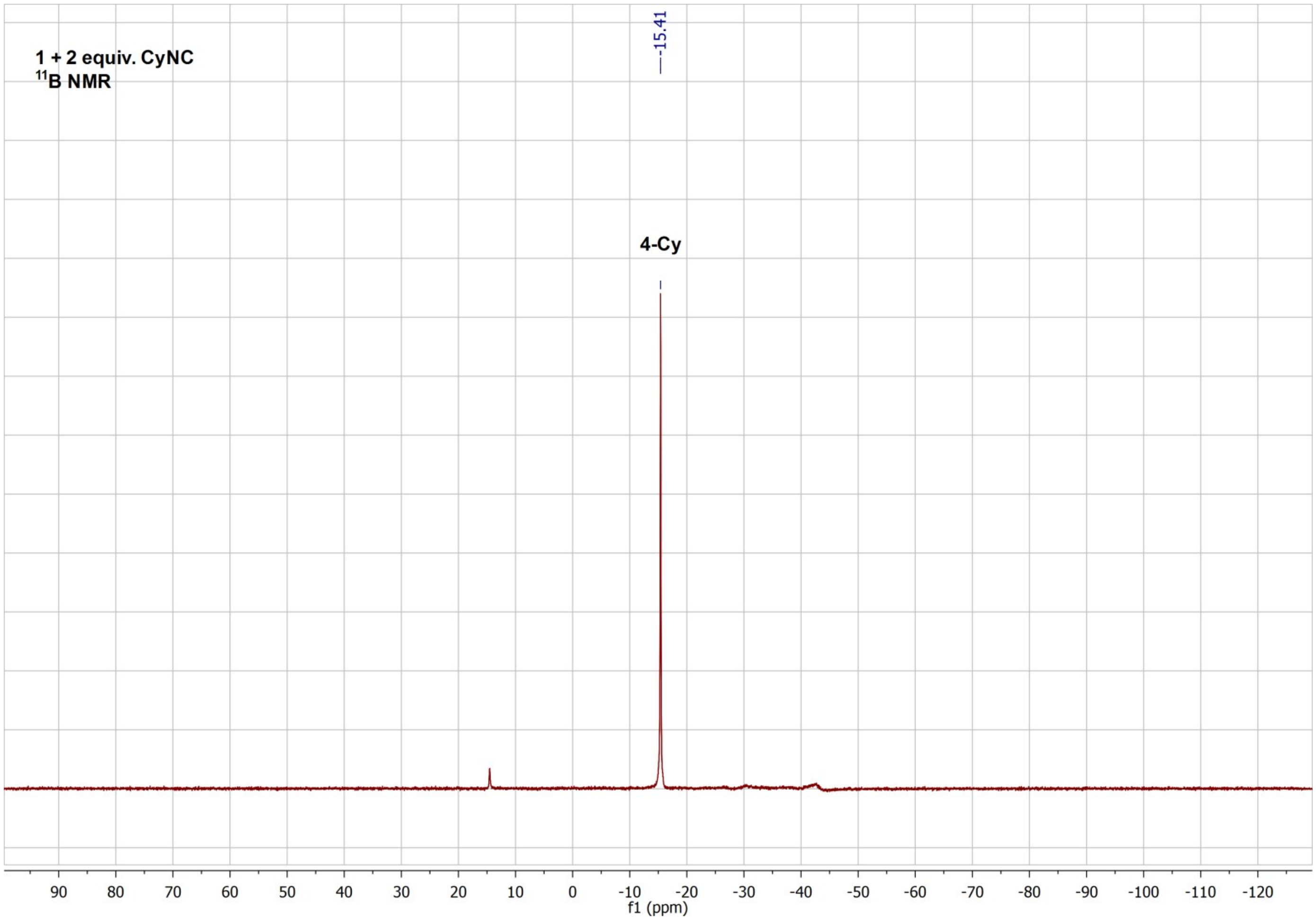

Figure S25. ^{11}B NMR of a 1:2 mixture of **1** and CyNC in d_8-THF after 15 min.

[(NHC)$_2$BCNBu^t]Cl, 4-Bu^t. The procedure for the synthesis for **4-Cy** was followed, using **1** (150 mg, 0.18 mmol) and CNBu^t (40 µL, 0.36 mmol). The work-up was also as indicated for **4-Cy**. Compound **4-Bu^t** was isolated as X-ray quality yellow plates (40 mg, 46 %). M.p.: 108-115 ^{o}C (dec.); ^{1}H NMR (d_2-DCM, 200 MHz, 298 K): δ = 1.23 (s, 9H, Bu^t-C*H*$_3$), 1.39 (d, $^3J_{HH}$ = 7.2 Hz, 24H, NHC-Pr^i-C*H*$_3$), 2.32 (s, 12H, NHC-NC*Me*), 4.98 (sept, $^3J_{HH}$ = 7.2 Hz, 4H, NHC-Pr^i-C*H*); ^{13}C{^{1}H} NMR (d_2-DCM, 75.5 MHz, 298 K): δ = 10.4 (Bu^t-*C*H$_3$), 21.1 (NHC-Pr^i-*C*H$_3$), 22.6 (Bu^t-*C*), 29.5 (NHC-Pr^i-*C*H), 51.4 (NHC-NC*Me*), 126.7 (NHC-N$\underline{C}$Me); ^{11}B NMR (d_2-DCM, 128 MHz, 298 K): -14.7 (s, *B*(CNBu^t)(NHC)$_2$); IR, ν/cm^{-1} (ATR): 1865 (C=N); MS/ESI m/z (%): 398 ([M-Cl-(*iso*-butene)]$^+$, 100%); anal. calcd. for $C_{27}H_{49}BClN_5$: C, 65.91 %; H, 10.45 %; N, 14.23 %; found: C, 63.07 %; H, 10.51 %; N, 14.36 %.

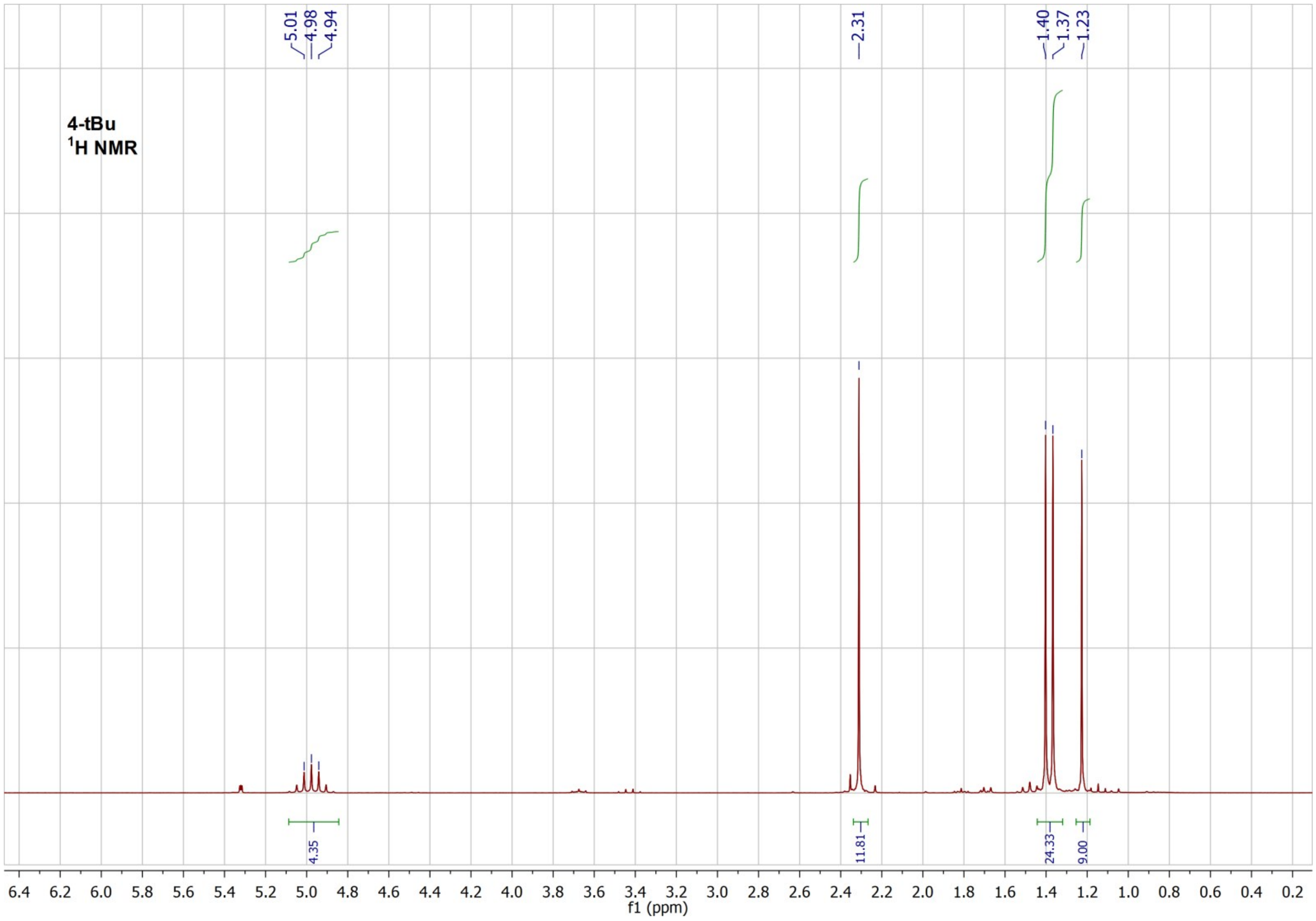

Figure S26. ^{1}H NMR spectrum of **4-Bu^t** in d_2-DCM.

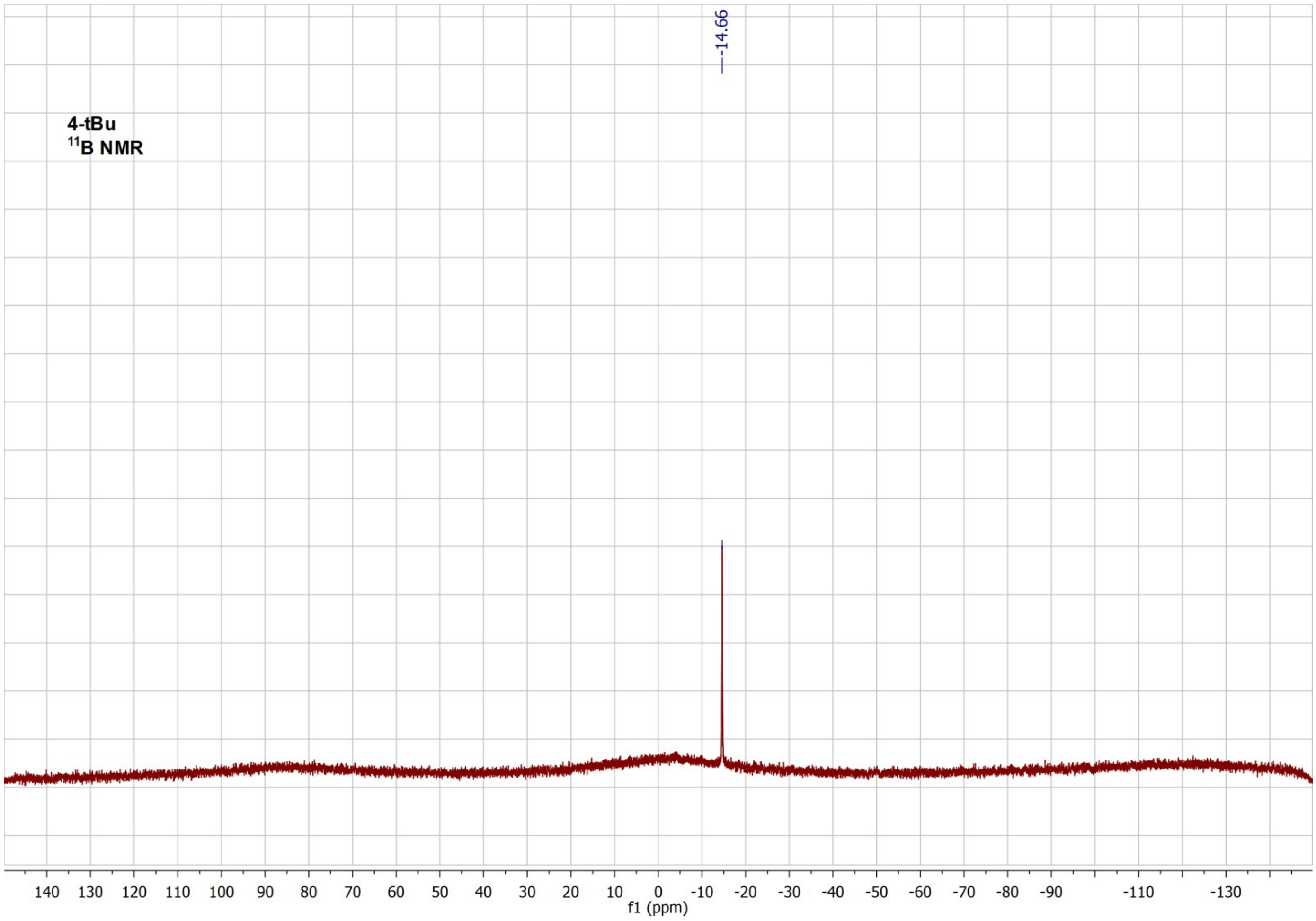

Figure S27. ^{11}B NMR spectrum of **4-Bu^t** in d_2-DCM.

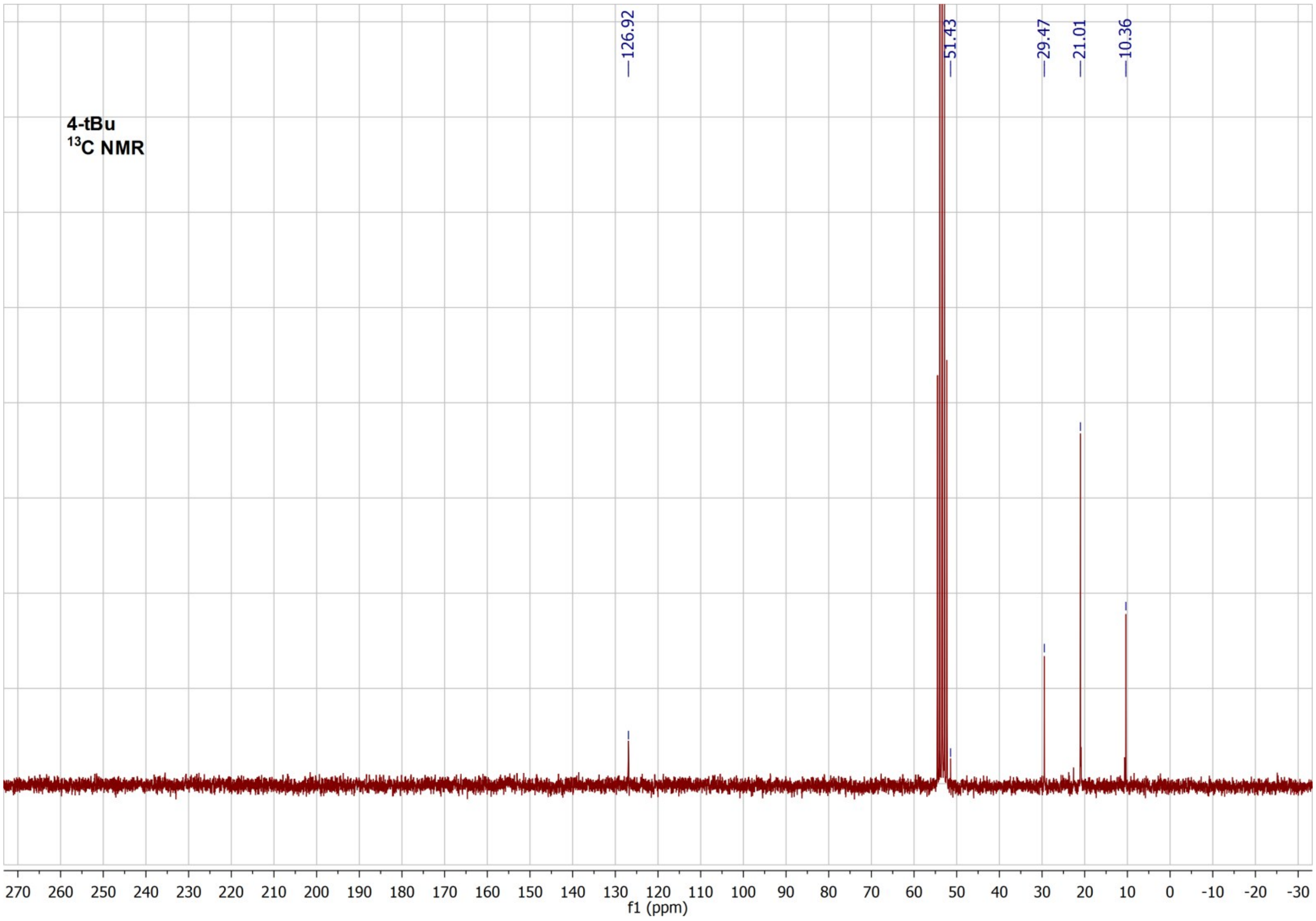

Figure S28. ^{13}C NMR spectrum of **4-Bu^{t}** in d_2-DCM.

[(NHC)$_2$B(CN)(Bz)]Cl, 5-Bz. The procedure for the synthesis for **4-Cy** was followed, using **1** (100 mg, 0.12 mmol) and a toluene solution of CNBz (0.47 mL, 0.5 M). The work-up was also as indicated for **4-Cy**. Compound **5-Bz** was isolated as X-ray quality colorless needles (35 mg, 57 %). M.p.: 112-118 ^{o}C (melt); ^{1}H NMR (d_2-DCM, 400 MHz, 298 K): δ =1.27 (br, 12H, NHC-Pr^{i}-C*H*$_3$), 1.52 (br, 12H, NHC-Pr^{i}-C*H*$_3$), 2.46 (s, 12H, NHC-NC*Me*), 2.67 (br, 2H, PhC*H*$_2$N), 4.98 (br, 4H, NHC-Pr^{i}-C*H*), 7.13 (m, 5H, Ar-C*H*); ^{13}C{^{1}H} NMR (d_2-DCM, 75.5 MHz, 298 K): δ = 11.2 (NHC-Pr^{i}-*C*H$_3$), 20.3 (NHC-Pr^{i}-*C*H$_3$), 21.1 (NHC-Pr^{i}-*C*H), 50.9 (NHC-NC*Me*), 125.7 (NHC-N$\underline{C}$Me), 128.2, 128.8, 128.9, and 141.1 (Ar-*C*); ^{11}B NMR (d_2-DCM, 128 MHz, 298 K): -19.5 (s, *B*(CN)(Bz)); MS/ESI m/z (%): 488 ([M-Cl], 100%); anal. calcd. for $C_{27}H_{49}BClN_5$: C, 68.77 %; H, 9.04 %; N, 13.37 %; found: C, 67.43 %; H, 9.27 %; N, 13.18 %.

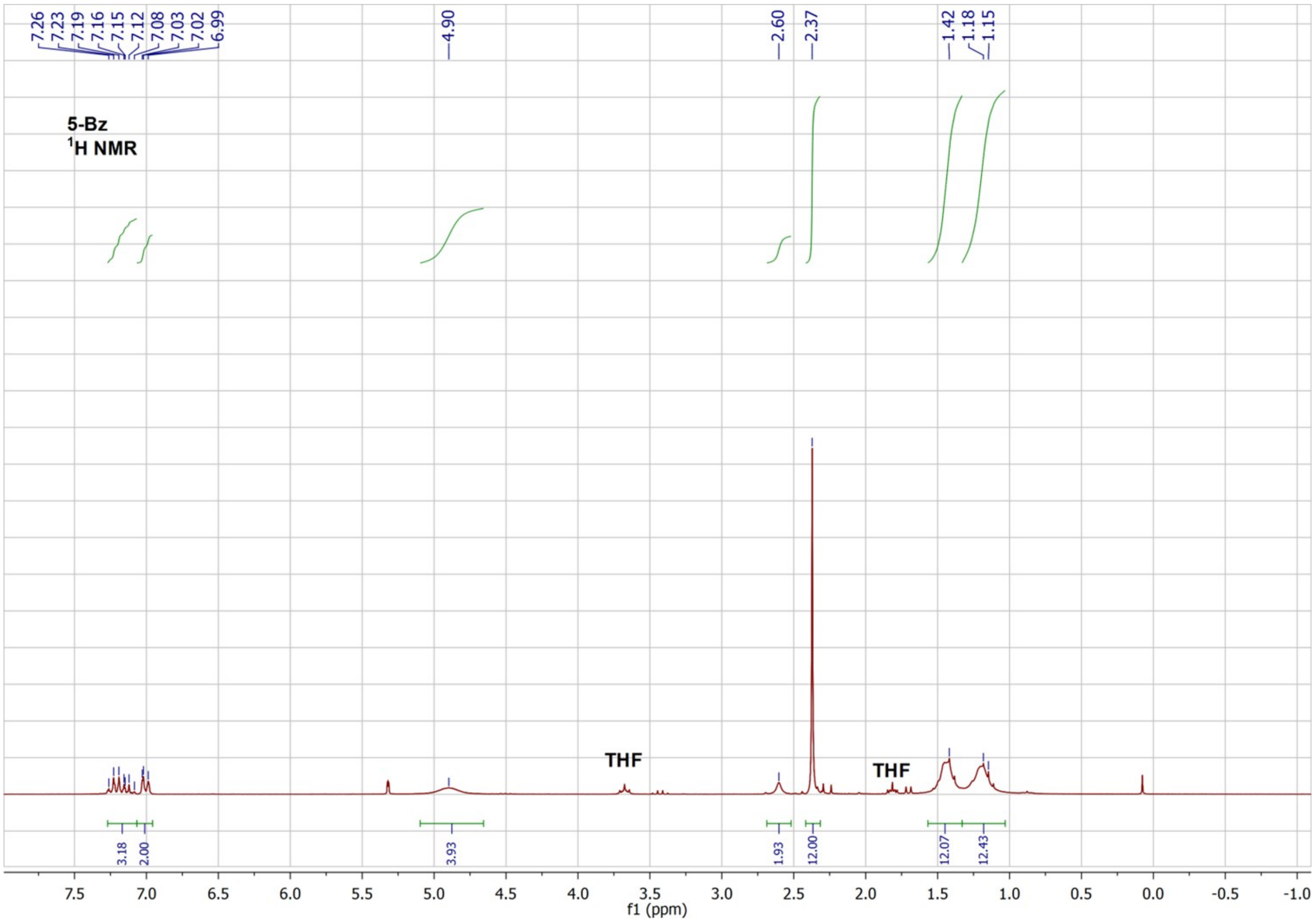

Figure S29. ^{1}H NMR spectrum of **5-Bz** in d_2-DCM.

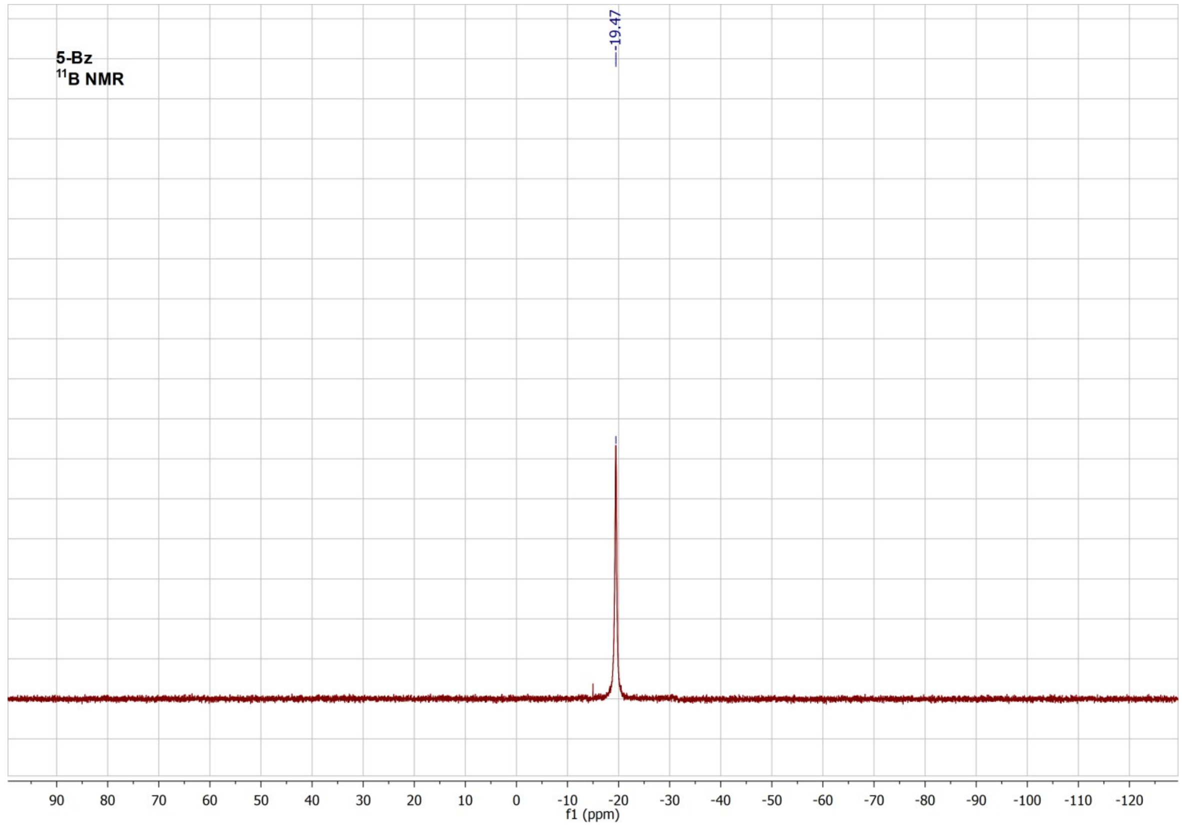

Figure S30. ^{11}B NMR spectrum of **5-Bz** in d_2-DCM.

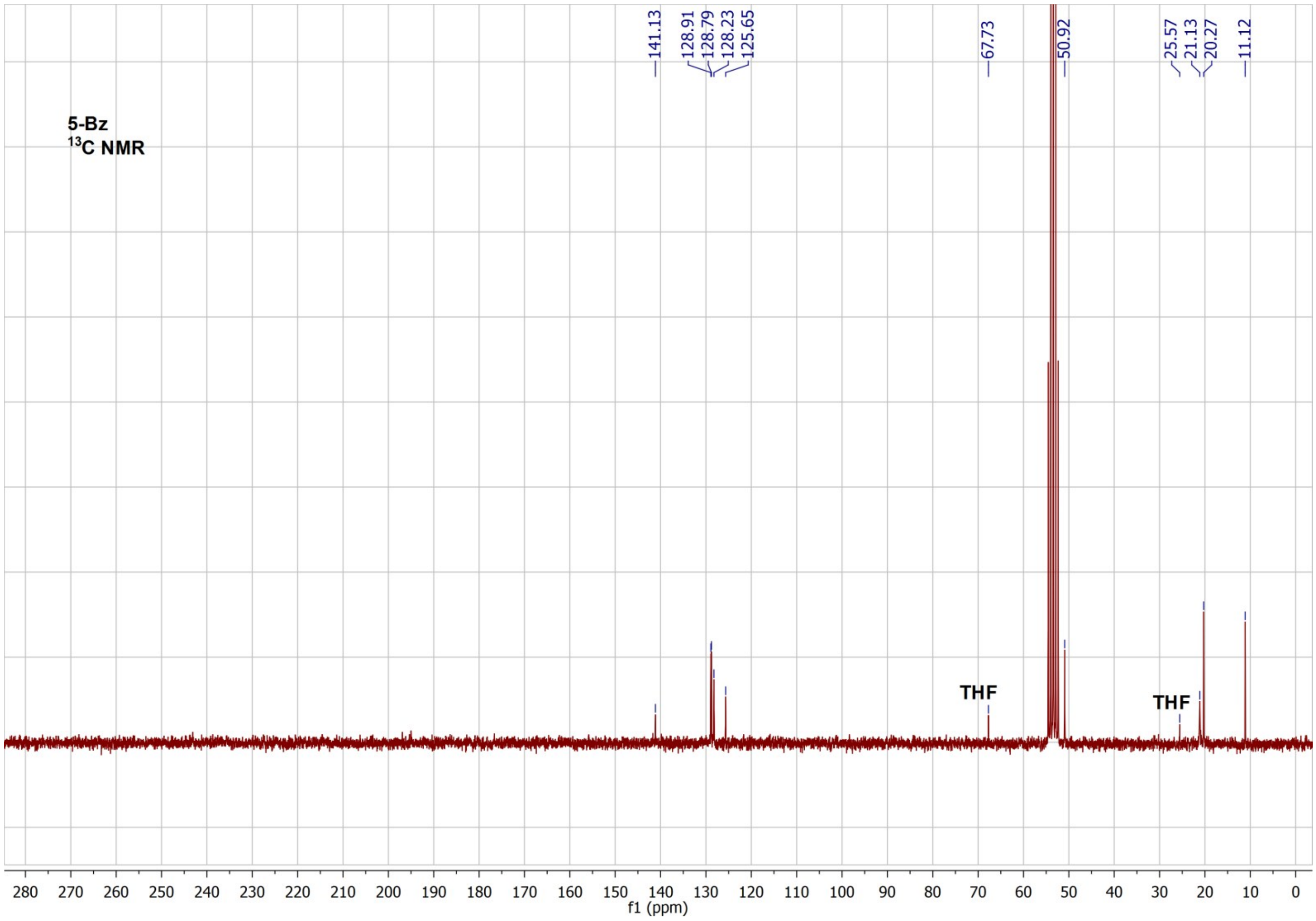

Figure S31. ^{13}C NMR spectrum of **5-Bz** in d_2-DCM.

$[(NHC)_2B(CN)H]Cl$, 5-H. Compound **4-Bu^t** was dissolved in CD_2Cl_2, and monitored by ^1H NMR spectroscopy for 1 week. After this time, the starting material had completely converted into a single compound assigned as **5-H**, with a second set of peaks attributable to *iso*-butene (Figure S4). ^1H NMR (DCM-d_2, 400 MHz, 298 K): δ =1.46 (d, $^3J_{HH}$ = 7.2 Hz, 12H, NHC-Pr^i-C*H*$_3$), 1.50 (d, $^3J_{HH}$ = 7.2 Hz, 12H, NHC-Pr^i-C*H*$_3$), 2.35 (s, 12H, NHC-NC*Me*), 3.22 (br, 1H, B*H*), 4.96 (sept, $^3J_{HH}$ = 7.2 Hz, 4H, NHC-Pr^i-C*H*), 7.13 (m, 5H, Ar-C*H*); $^{13}C\{^1H\}$ NMR (DCM-d_2, 75.5 MHz, 298 K): δ = 10.7 (NHC-Pr^i-*C*H), 20.9 and 21.1 (NHC-Pr^i-*C*H$_3$), 51.4 (NHC-NC*Me*), 127.7 (NHC-N$\underline{C}$Me); ^{11}B (DCM-d_2, 128 MHz, 298 K): -31:1 (d, $^1J_{BH}$ = 91 Hz, *B*(CN)H); $^{11}B\{^1H\}$ (DCM-d_2, 128 MHz, 298 K): -31.1 (s); IR, ν/cm^{-1} (ATR): 2401 (CN); MS/ESI m/z (%): 432 ([M-H], 3%), 398 ([M-Cl]$^+$, 100%).

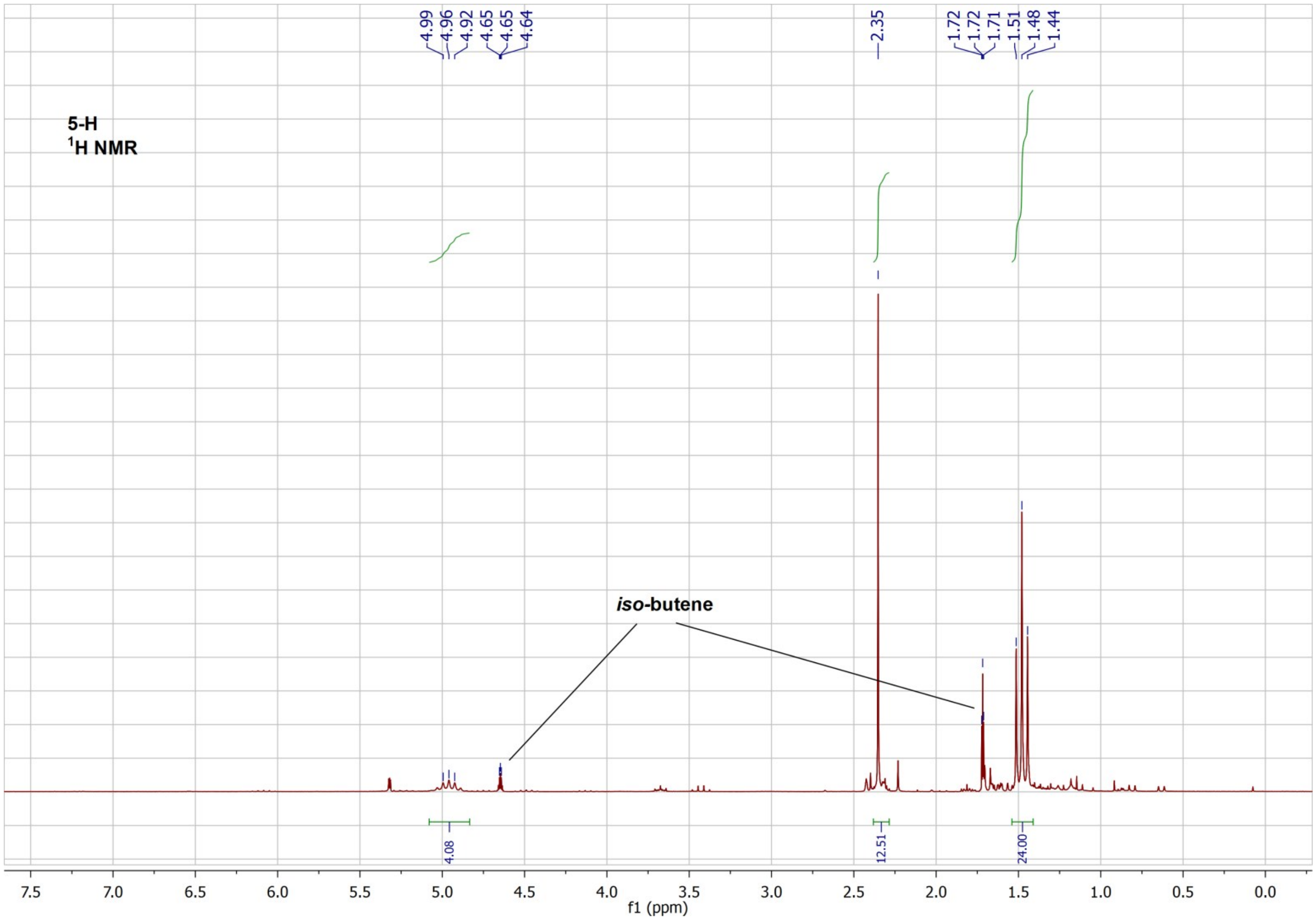

Figure S32. ^{1}H NMR spectrum of **5-Bz** in d_2-DCM showing the formation of *iso*-butene.

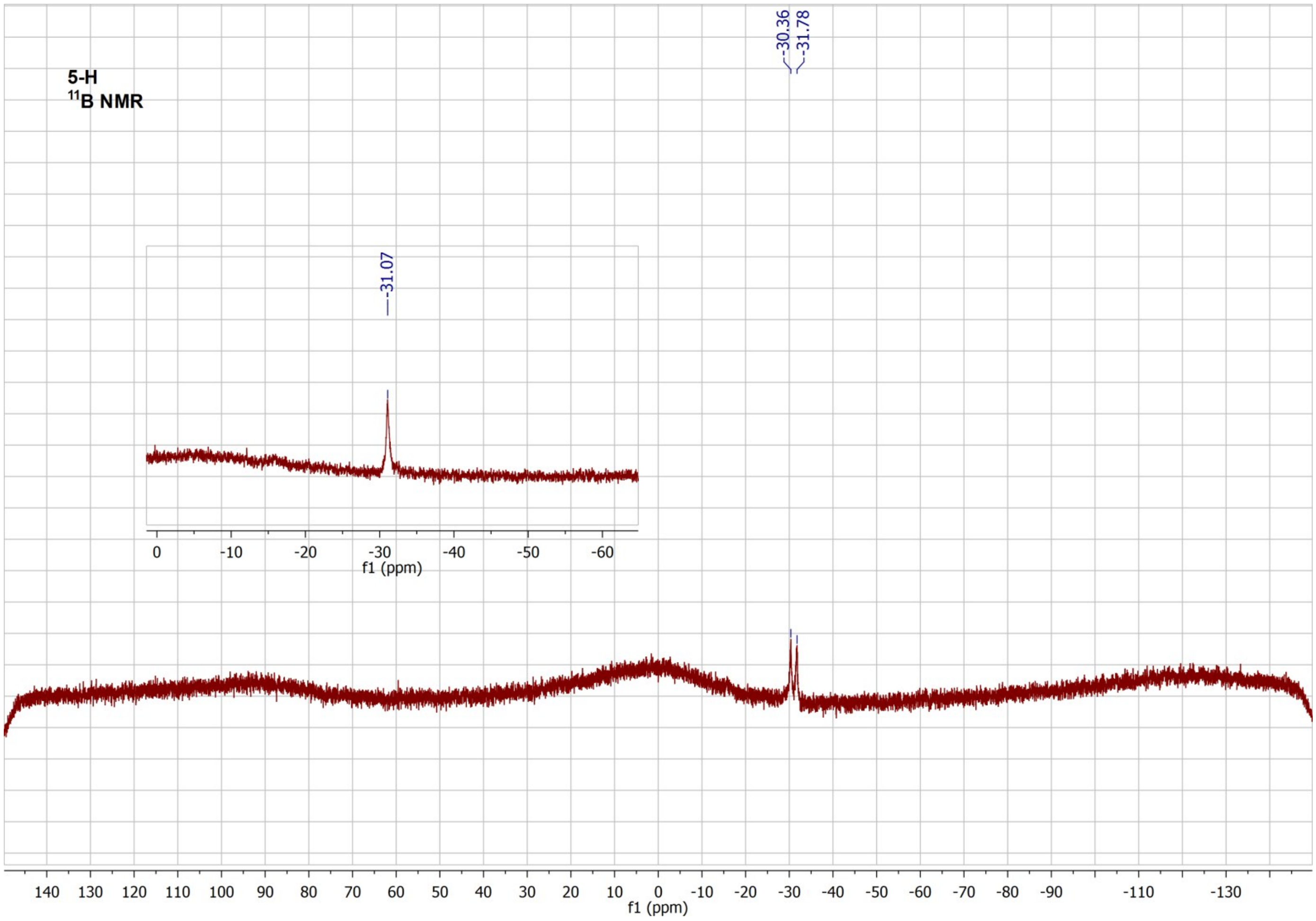

Figure S33. ^{11}B NMR spectrum of **5-H** in d_2-DCM; inset:$^{11}B\{^1H\}$ NMR spectrum.

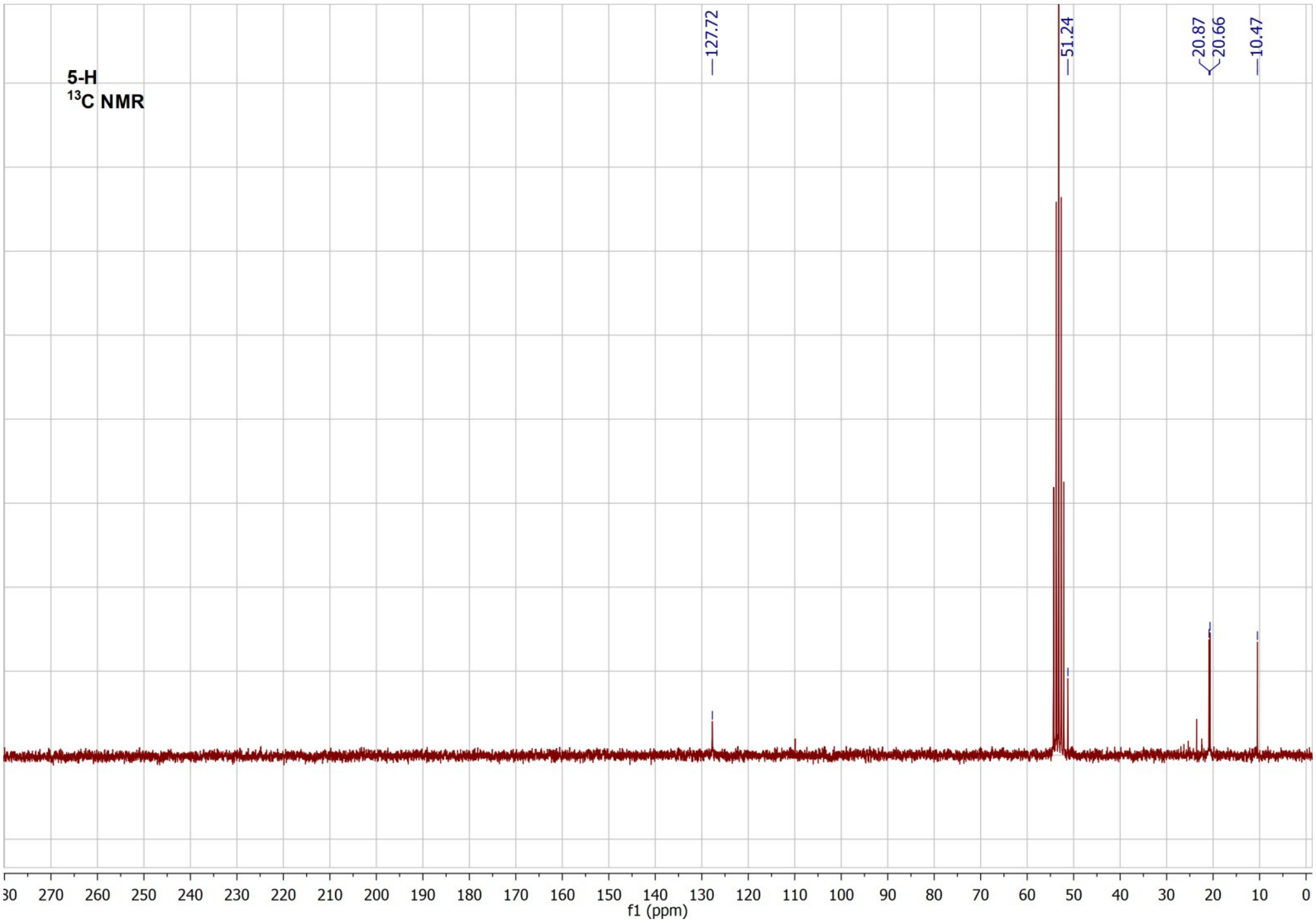

Figure S34. ^{13}C NMR spectrum of **5-H** in d_2-DCM.

IR spectra of all compounds:

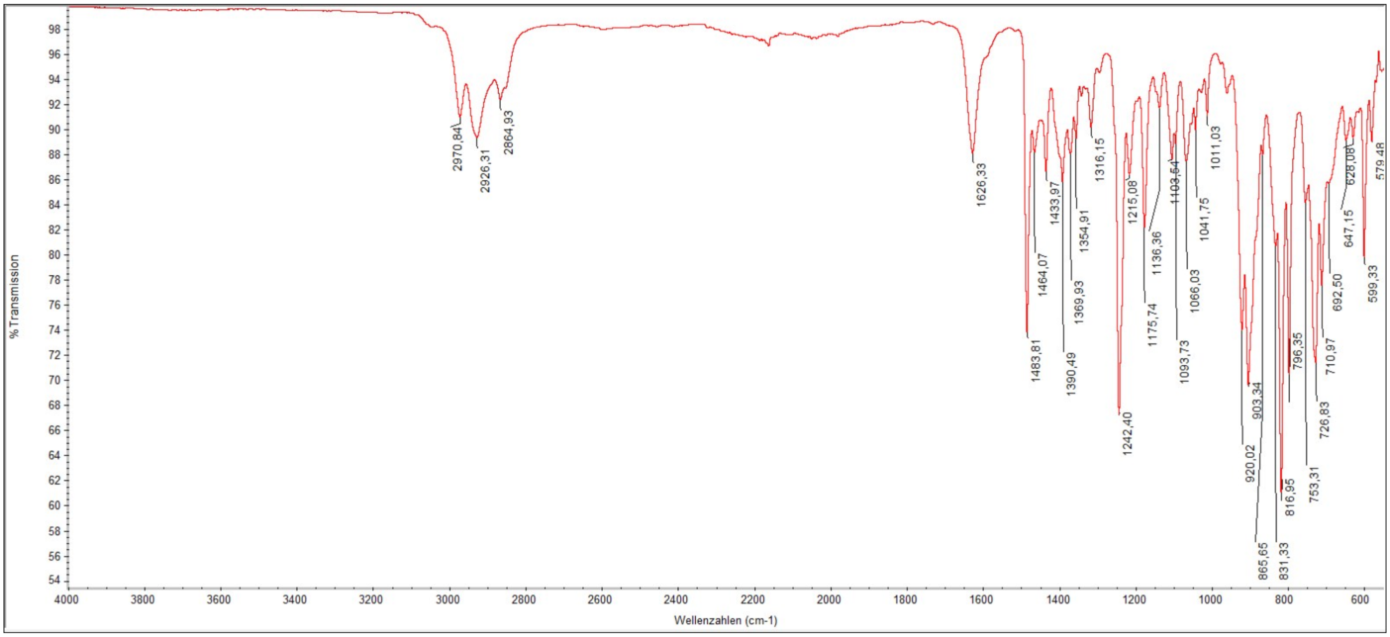

Figure S35. ATR IR spectrum of **2-Cy**.

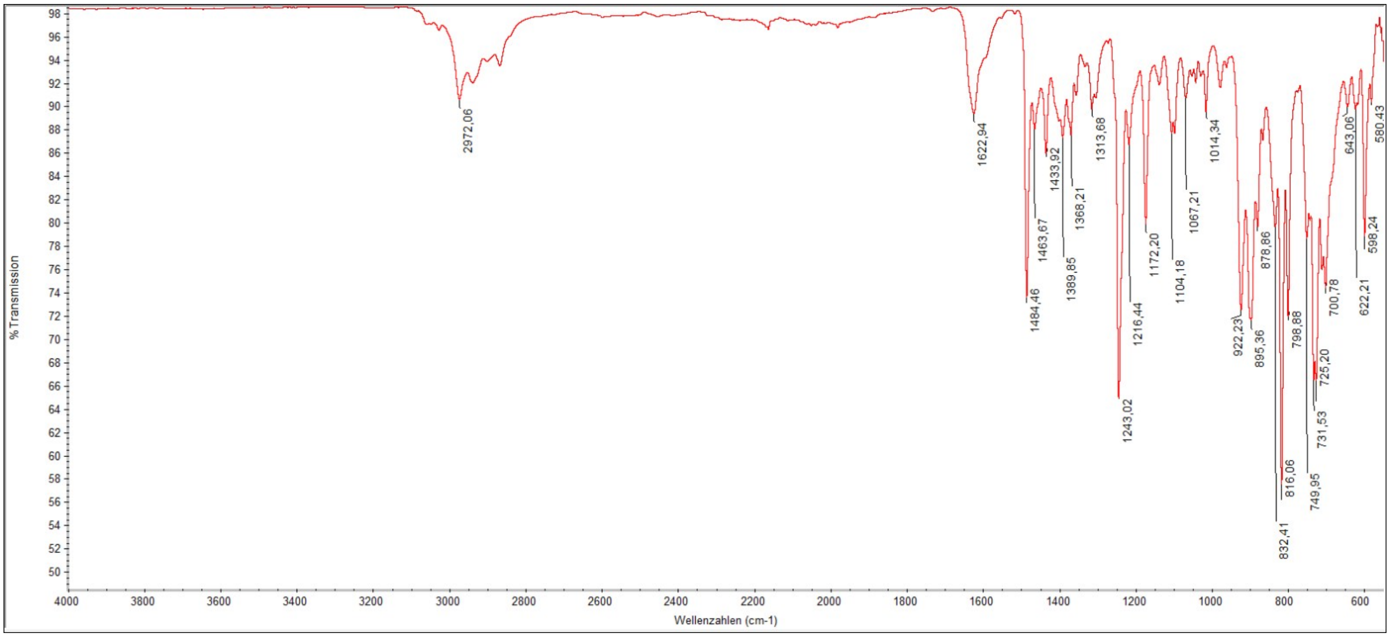

Figure S36. ATR IR spectrum of **2-Bz**.

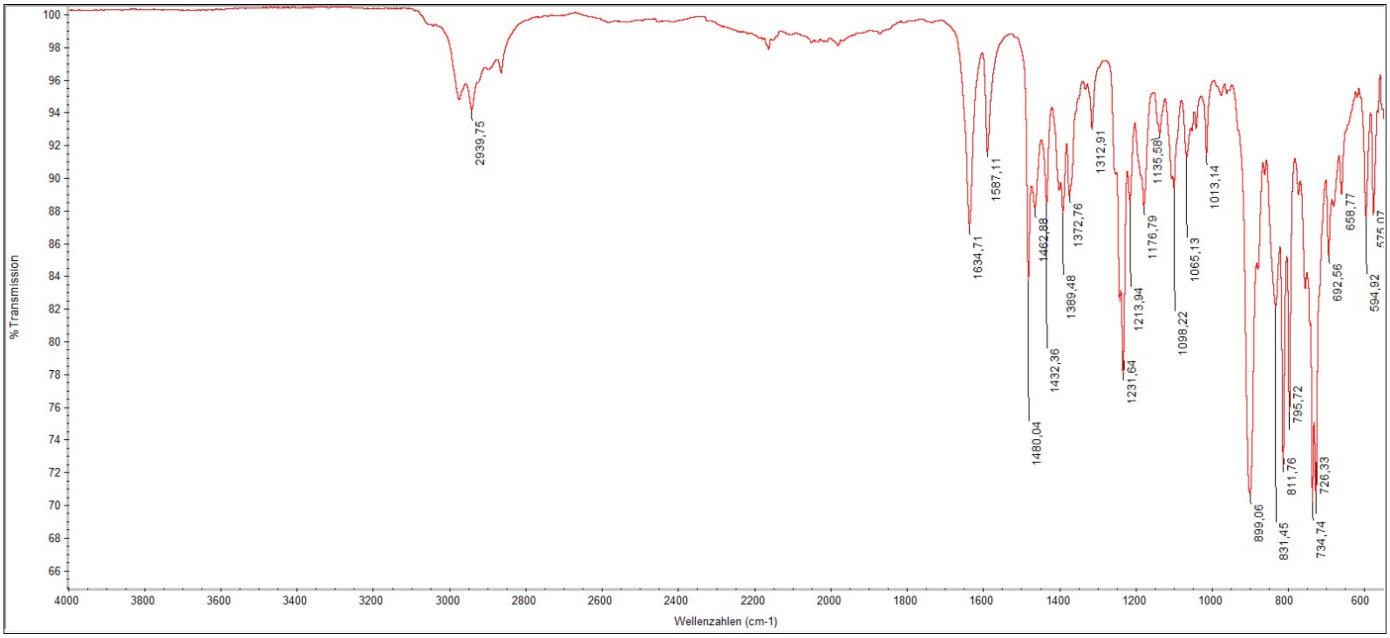

Figure S37. ATR IR spectrum of **2-Xyl**.

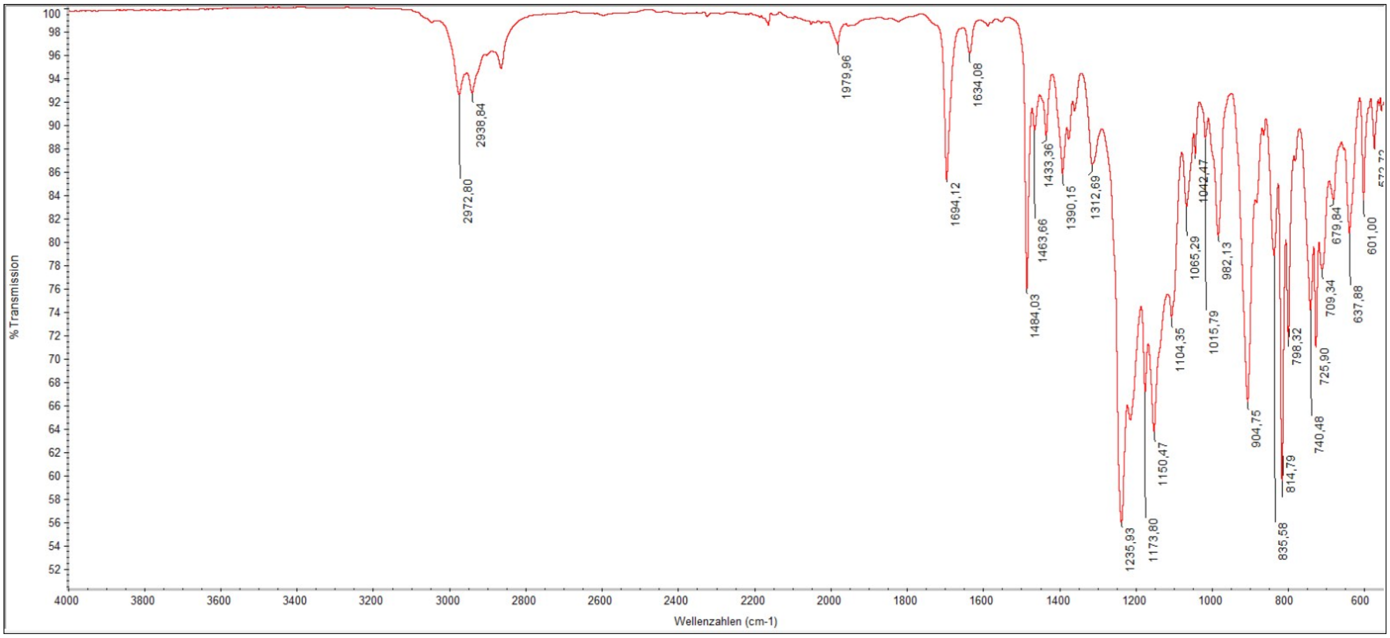

Figure S38. ATR IR spectrum of **2-CO**.

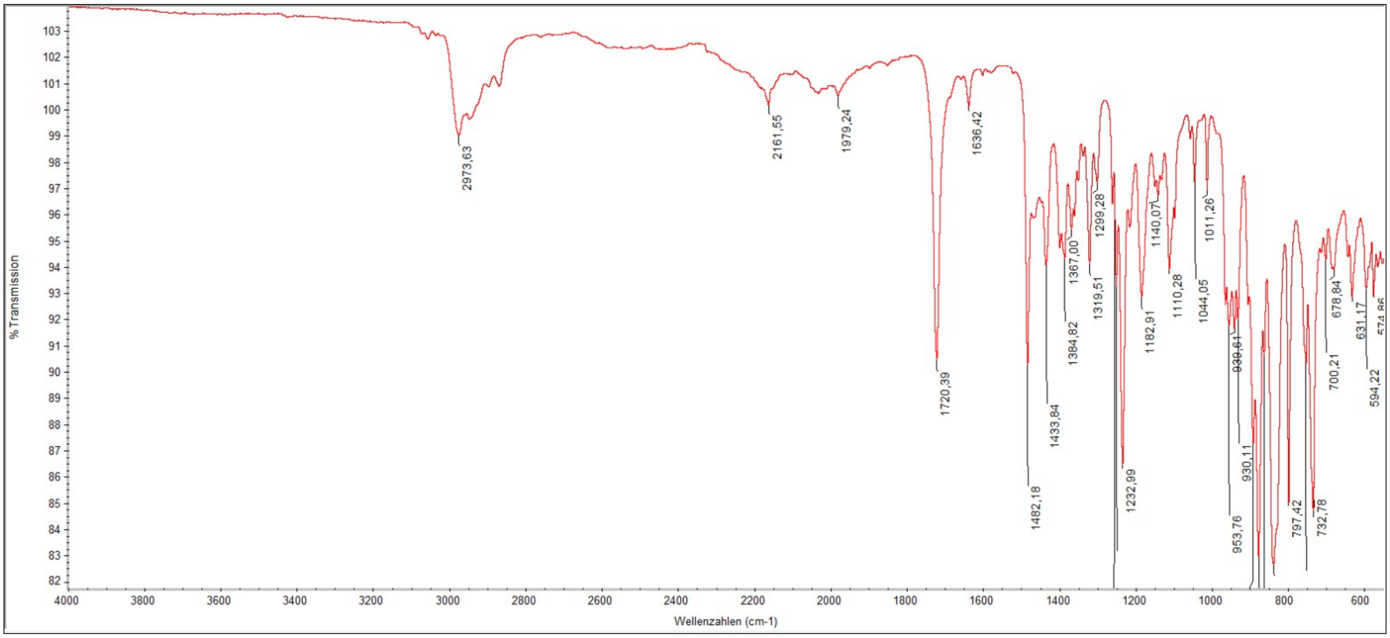

Figure S39. ATR IR spectrum of compound **3**.

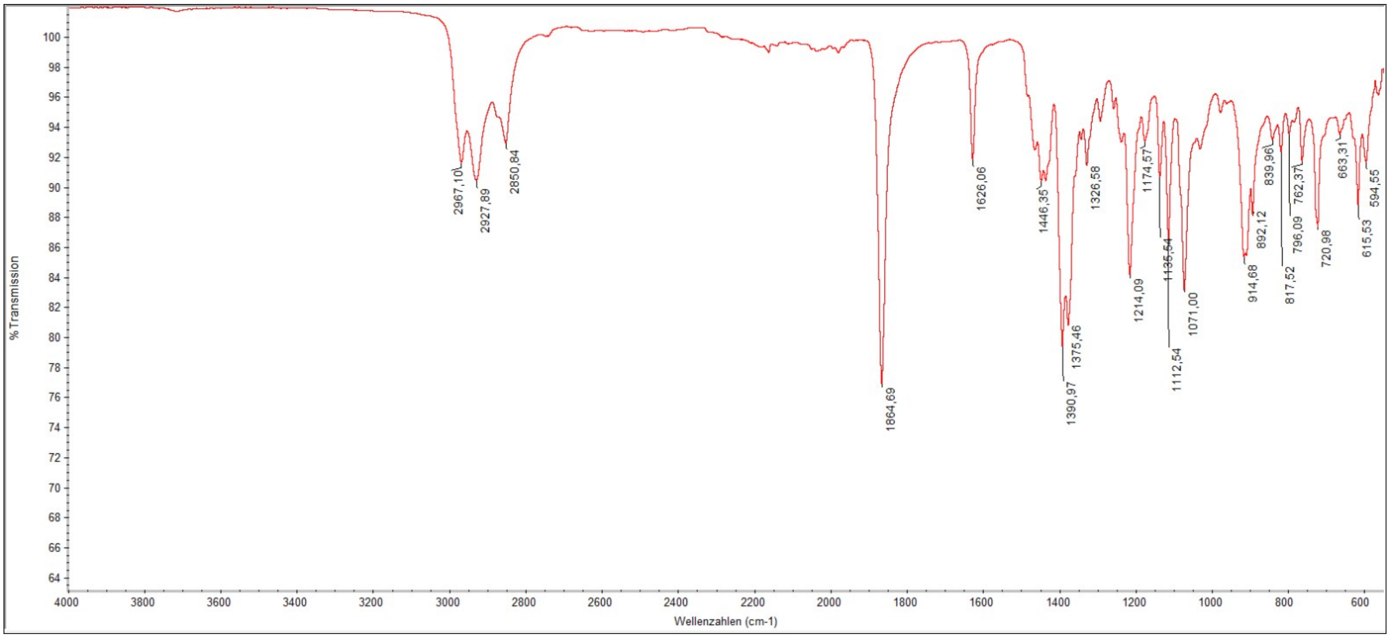

Figure S40. ATR IR spectrum of **4-Cy**.

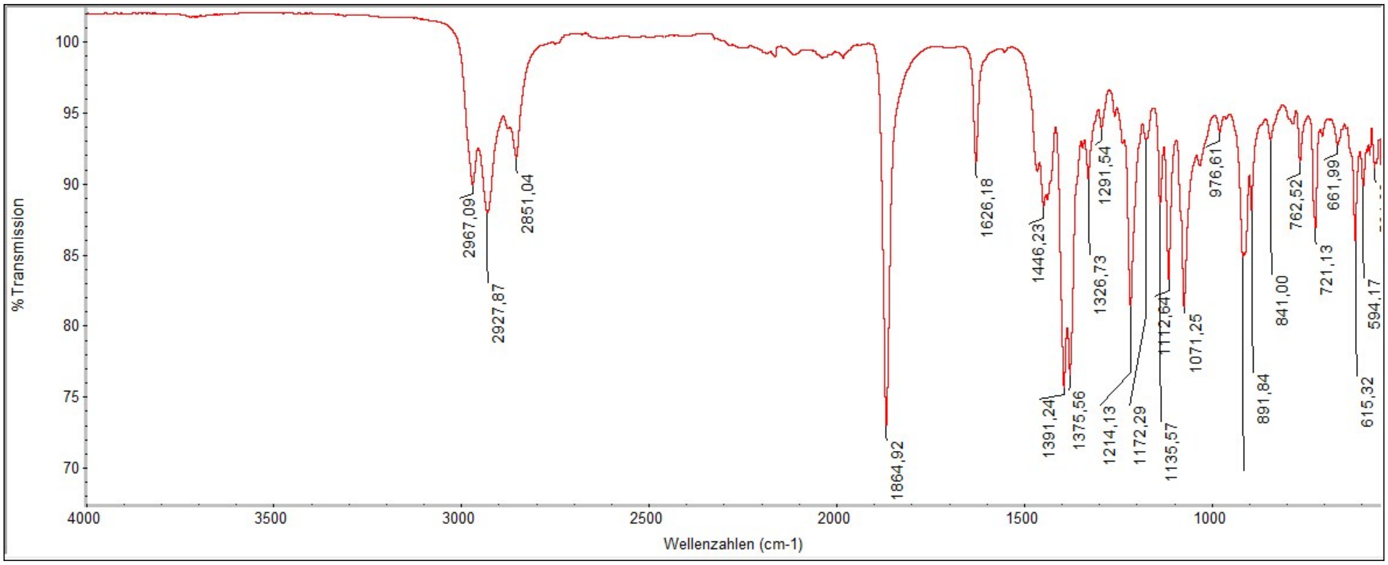

Figure S41. ATR IR spectrum of **4-tBu**.

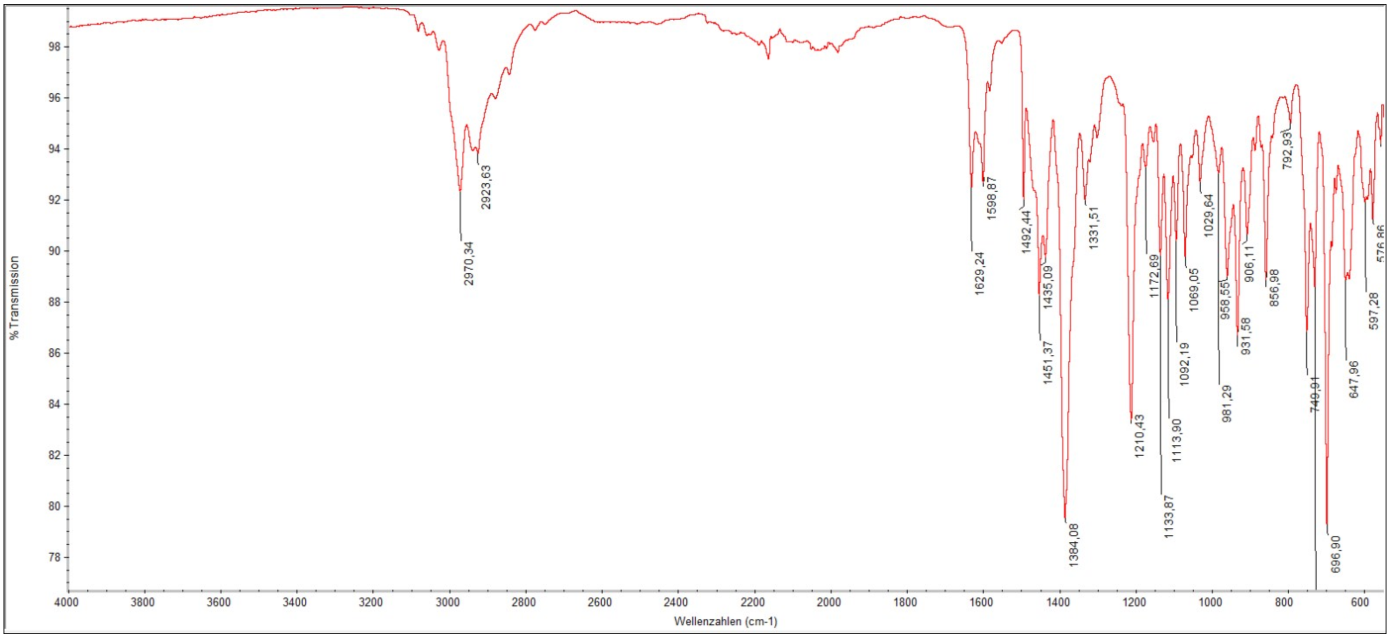

Figure S42. ATR IR spectrum of **5-Bz**.

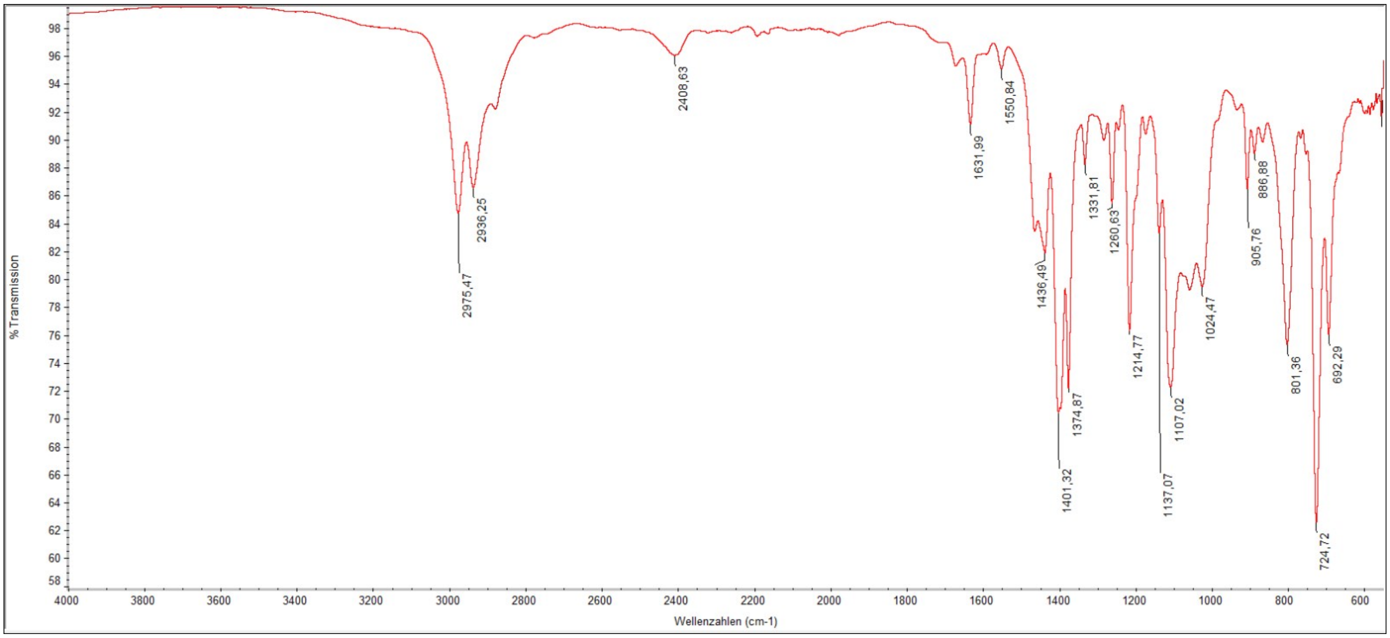

Figure S43. ATR IR spectrum of **5-H**.

2. X-Ray crystallographic data

Crystals were mounted on a glass capillary in perfluorinated oil and measured in a cold N_2 flow. The data was collected on an Oxford Diffraction SuperNova Atlas at 150K (Cu-Kα radiation, λ= 1.54184 Å). The structures were solved by direct methods or using the SHELXT program[5] and refined on F^2 with the SHELX-2016 software package.[6] The PLATON/SQUEEZE function was used to remove highly disordered solvent in **2-Cy** and **2-Bz**,[7] details of which can be found in the CIFs of those compounds. The positions of the H atoms were calculated and considered isotropically according to a riding model, aside from hydride ligands in **2-Cy**, **2-Xyl**, **2-Bz**, and **3** (H1), which were located and freely refined. Full crystal data, refinement and data collection details for **2-Cy**, **2-Xyl**, **2-Bz**, **3**, **4-Cy**, **4-^{t}Bu**, and **5-Bz** can be found in their CIFs, with key parameters summarized in Table S1.

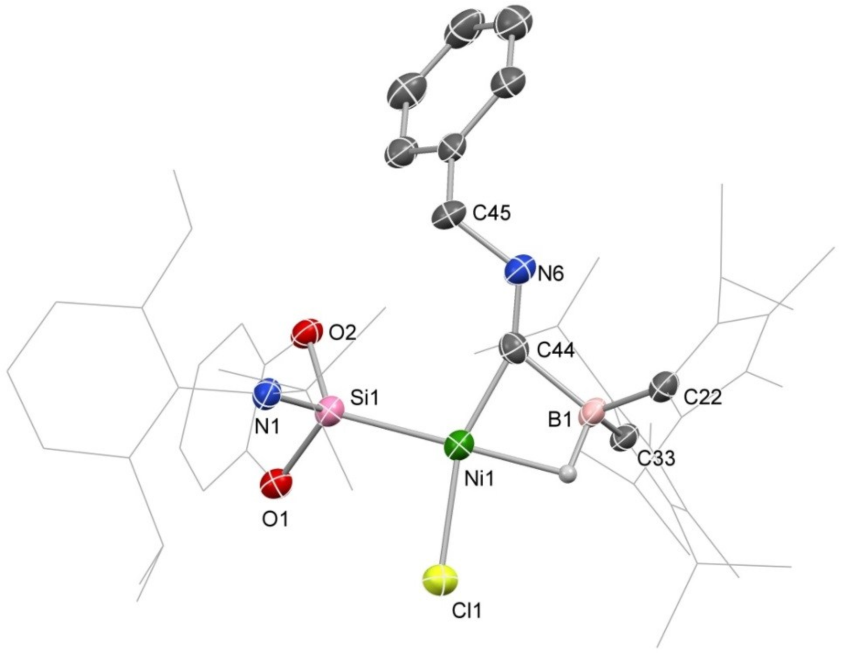

Figure S44. Molecular structure of **2-Bz** with thermal ellipsoids at 30% probability. Selected bond lengths (Å) and angles (°): B1-Ni1 2.235(6); B1-C44 1.65(1); Ni1-C44 1.819(9); Ni1-Si1 2.184(2); C44-N6 1.27(1); Ni1-C44-N6 153.0(7); B1-C44-N6 125.2(7); B1-C44-Ni1 80.8(5); B1-Ni1-Cl1 112.9(2); Si1-Ni1-C44 103.5(3); Si1-Ni1-Cl1 97.74(9).

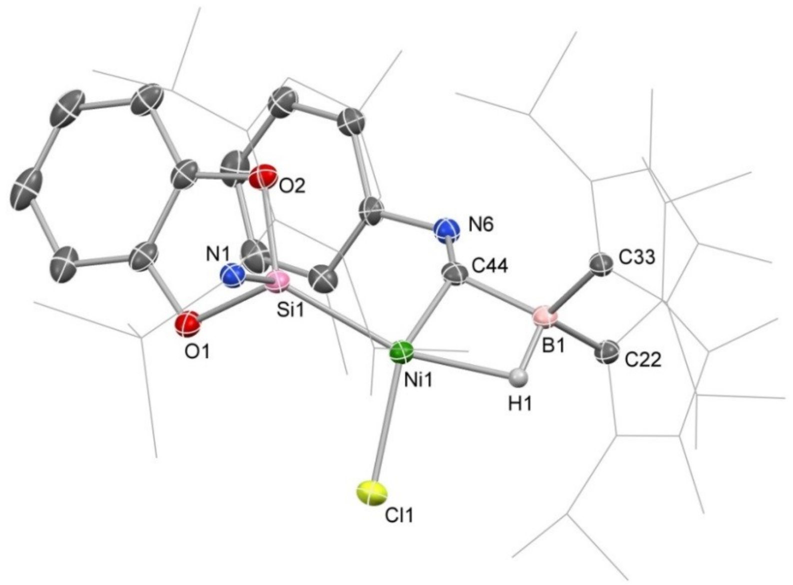

Figure S45. Molecular structure of **2-Xyl** with thermal ellipsoids at 30% probability. Selected bond lengths (Å) and angles (°): B1-Ni1 2.289(3); B1-C44 1.629(5); Ni1-C44 1.805(3); Ni1-Si1 2.1896(9); C44-N6 1.261(4); Ni1-C44-N6 144.3(2); B1-C44-N6 127.1(2); B1-C44-Ni1 83.5(2); B1-Ni1-Cl1 117.10(8); Si1-Ni1-C44 99.17(9); Si1-Ni1-Cl1 105.30(3).

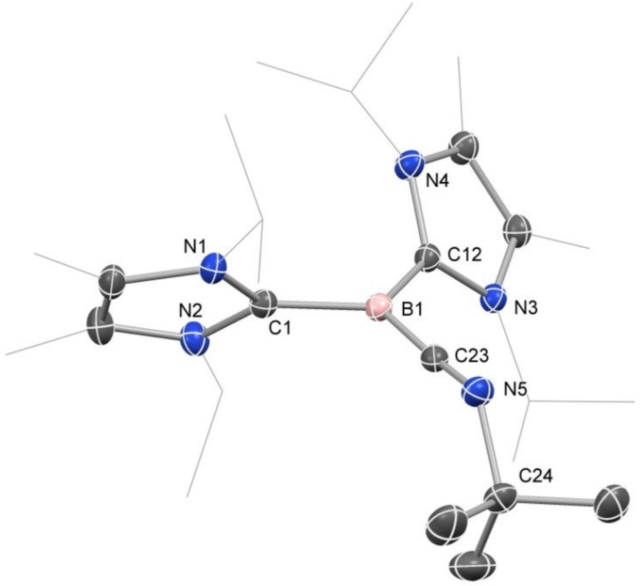

Figure S46. Molecular structure of **4-^{t}Bu** with thermal ellipsoids at 30% probability, and chloride counter ion omitted. Selected bond lengths (Å) and angles (°): B1-C23 1.433(2); B1-C1 1.576(2); B1-C12 1.568(2); C23-N5 1.220(2); C1-B1-C12 120.0; C1-B1-C23 116.7(1); C12-B1-C23 123.3(1); B1-C23-N5 171.4(1); C23-N5-C24 127.9(1).

Table S1. Summary of Crystallographic Data for **2-Cy**, **2-Bz**, **2-Xyl**, **3**, **4-Cy**, **4-Bu^t**, and **5-Bz**.

	**2-Cy**	**2-Bz**	**2-Xyl**·3.5(THF)	**3**	**4-Cy**·(THF)	**4-Bu^t**	**5-Bz**·2(THF)
empirical form.	$C_{50}H_{82}BClN_6NiO_2Si_2$	$C_{51}H_{78}BClN_6NiO_2Si_2$	$C_{66}H_{105}BClN_6NiO_6Si_2$	$C_{44}H_{71}BClN_5NiO_3Si_2$	$C_{33}H_{59}BClN_5O$	$C_{27}H_{49}BClN_5$	$C_{38}H_{63}BClN_5O_2$
formula wt	960.36	968.34	1239.70	879.20	588.11	489.97	668.19
crystal syst.	monoclinic	monoclinic	triclinic	monoclinic	orthorhombic	monoclinic	triclinic
space group	*C2/c*	*P2$_1$/c*	*P-1*	*P2$_1$/c*	*Pbca*	*P2$_1$/n*	*P-1*
a (Å)	27.8322(3)	14.0633(7)	11.0148(4)	10.87820(10)	15.6878(2)	13.7173(2)	9.8905(12)
b (Å)	21.4898(2)	26.4632(11)	14.6131(5)	25.2119(2)	12.34570(10)	15.3059(2)	12.6054(14)
c (Å)	26.4066(3)	18.1718(10)	22.7930(8)	17.86600(10)	36.8495(5)	15.7157(2)	16.945(2)
α (deg.)	90	90	84.552(3)	90	90	90	69.660(11)
β (δεγ)	116.468(2)	100.801(5)	83.601(3)	91.9480(10)	90	110.275(2)	89.159(10)
γ (deg.)	90	90	70.398(4)	90	90	90	78.594(10)
vol (Å^3)	14138.5(3)	6643.0(6)	3428.1(2)	4897.10(7)	7136.90(14)	3095.16(8)	1938.4(4)
Z	8	4	2	4	8	4	2
ρ(calc) (g.cm^{-3})	0.902	0.968	1.201	1.193	1.095	1.051	1.145
μ (mm^{-1})	1.311	1.401	1.508	1.859	1.172	1.241	1.157
F(000)	4144	2080	1338	1888	2576	1072	728
T (K)	150(2)	150(2)	150(2)	150(2)	150(2)	150(2)	150(2)
reflns collect.	27061	46775	24731	19886	27357	12159	13387
unique reflns	13278	11947	12907	9232	6743	5837	7283
R_{int}	0.0272	0.2168	0.0350	0.0200	0.0372	0.0177	0.0807
R1 [$I>2\sigma(I)$]	0.0364	0.2691	0.0455	0.0231	0.0267	0.0206	0.1065
*w*R2 (all data)	0.1268	0.2240	0.1660	0.0889	0.1974	0.1198	0.2981
CCDC No.	1579154	1579155	1579156	1579157	1579158	1579159	1579160

3. **Computational methods and data**

DFT calculations were performed at the B97-D/cc-pVTZ//B97-D/6-31G(d)[Ni:cc-pVTZ] level of theory[8] which has been successfully applied for reaction energies of low-valent main group compounds previously.[9] Stationary points on the potential energy surface (PES) were characterized by harmonic vibrational frequency calculations. Transition states, which had one imaginary frequency, were analysed by intrinsic reaction coordinate (IRC) calculations to confirm the corresponding intermediates. Electronic structure analyses were executed at the B97-D/cc-pVTZ level. Calculations were carried out using the GAUSSIAN 09 program suite.[10]

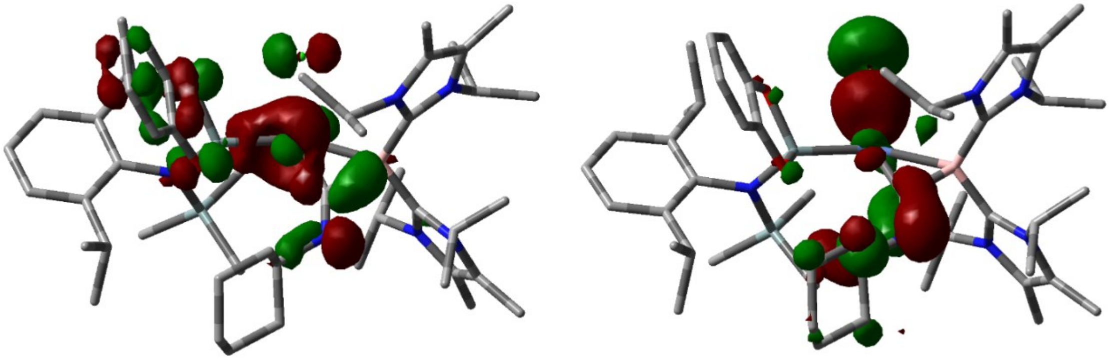

Figure S47. HOMO (left, -3.28 eV) and HOMO-6 (right, -4.38 eV) of **2-Cy**.

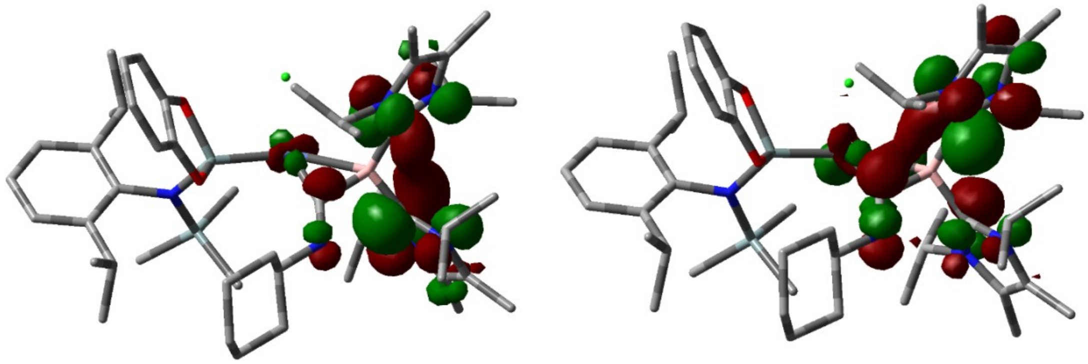

Figure S48. LUMO (left, -1.13 eV) and LUMO+1 (right, -1.03 eV) of **2-Cy**.

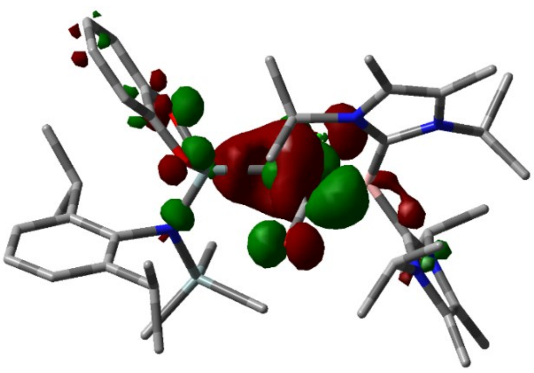

Figure S49. HOMO (left, -3.47 eV) and HOMO-5 (right, -4.59 eV) of **2-CO**.

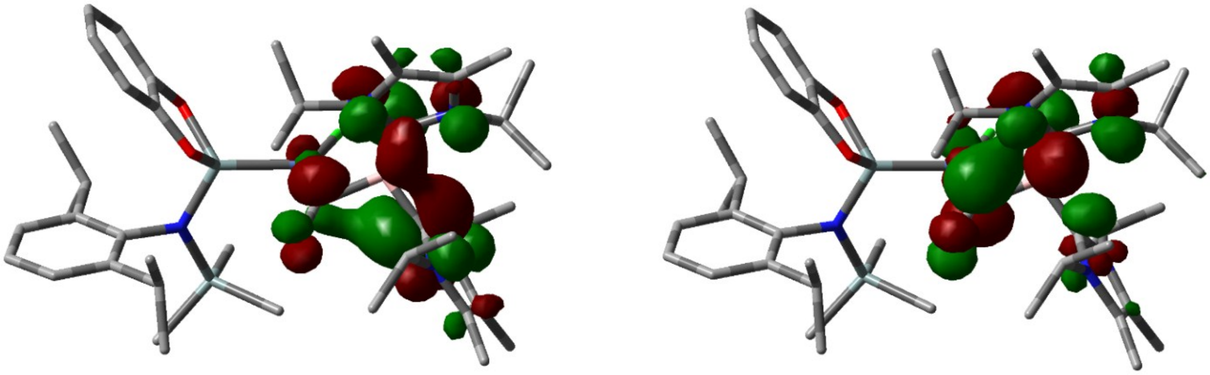

Figure S50. LUMO (left, -1.65 eV) and LUMO+1 (right, -1.47 eV) of **2-CO**.

Table S2. Cartesian geometry of **2-Cy** in Angstrom [Å].

Atomtype	X coordinates	Y coordinates	Z coordinates
Ni	0.29549	0.207508	-0.928067
Si	-2.229708	-2.393148	-1.307881
Cl	0.259654	1.265266	-2.949093
Si	-1.748609	0.500776	-0.321595
O	-2.11389	0.990336	1.329111
O	-2.467995	1.961567	-0.966739
N	1.049363	-0.783829	1.774218
N	2.800921	2.541762	0.369941
N	4.199362	-1.364781	0.943785
C	-2.759348	2.194956	1.334029
N	-2.826598	-0.821606	-0.747007
C	3.27027	-1.274717	-0.049847
N	4.116927	1.749282	-1.17601

N	3.250659	-2.484552	-0.676126
C	1.012814	-0.300318	0.594473
C	-2.956297	2.738474	0.040332
C	-1.184464	-3.215406	0.054884
H	-0.254538	-2.656856	0.241552
H	-0.919865	-4.252986	-0.214442
H	-1.747189	-3.245174	1.001912
C	-0.140474	-1.02388	2.581762
H	-1.048244	-1.034286	1.955053
C	-4.250799	-0.589544	-0.800564
C	3.089305	1.414165	-0.338153
C	4.306877	-0.335196	2.015814
H	3.4317	0.301373	1.863792
C	-0.042895	-2.380997	3.305968
H	0.859105	-2.380032	3.945414
H	0.073512	-3.187045	2.564686
C	-0.272708	0.117	3.615969
H	-0.351776	1.071798	3.077579
H	0.647783	0.134668	4.228618
C	-4.842013	-0.11237	-2.002891
C	-5.055049	-0.873451	0.335655
C	-3.169922	2.884732	2.476632
H	-3.00519	2.450249	3.464128
C	1.648816	2.64815	1.316871
H	1.346466	1.615333	1.492275
C	3.627459	3.592115	-0.035608
C	4.458347	3.093721	-1.010709
C	-1.300419	-2.609167	4.170999
H	-2.185016	-2.641632	3.511215
H	-1.241275	-3.584279	4.683737
C	-4.453074	-1.318074	1.669231
H	-3.364199	-1.205596	1.598845
C	2.218252	-2.810983	-1.707429
H	1.369078	-2.164898	-1.455409
C	4.226337	-3.322455	-0.11917
C	-3.578584	3.977838	-0.121216
H	-3.722509	4.387299	-1.12231
C	-1.50976	-0.092012	4.508274
H	-2.403224	-0.014903	3.871216
H	-1.575124	0.710505	5.263524
C	3.595383	4.975837	0.539058
H	4.018421	5.008303	1.556233
H	2.571625	5.370846	0.591206

H	4.186381	5.652392	-0.093321
C	-6.240955	0.014522	-2.061683
H	-6.699137	0.373041	-2.986502
C	0.473714	3.378273	0.652979
H	0.6945	4.441128	0.47055
H	-0.40215	3.319389	1.314438
H	0.217046	2.897173	-0.302439
C	-4.011064	0.260843	-3.22948
H	-2.949986	0.191441	-2.961607
C	4.63164	0.828586	-2.226266
H	4.23406	-0.150567	-1.94782
C	-1.482919	-1.471812	5.199358
H	-0.642625	-1.501991	5.91782
H	-2.410044	-1.626926	5.777215
C	5.580257	0.510209	1.866394
H	6.489861	-0.093494	2.008579
H	5.577211	1.30367	2.630312
H	5.617056	0.98348	0.875151
B	2.343105	-0.025406	-0.360526
C	-1.188779	-2.235184	-2.895701
H	-1.780022	-1.76841	-3.699156
H	-0.853724	-3.225739	-3.25476
H	-0.306268	-1.592574	-2.753254
C	-6.451127	-0.737881	0.225043
H	-7.077028	-0.971356	1.089426
C	4.819038	-2.620204	0.902457
C	6.164711	0.711644	-2.210861
H	6.658924	1.597201	-2.631827
H	6.447714	-0.154409	-2.828835
H	6.538196	0.542663	-1.189318
C	-4.942135	-0.423529	2.830489
H	-4.70724	0.633032	2.638973
H	-4.45588	-0.730701	3.771213
H	-6.031401	-0.51721	2.973224
C	-3.717988	-3.507985	-1.704978
H	-4.384466	-3.647835	-0.84129
H	-3.362302	-4.502163	-2.029156
H	-4.331426	-3.085629	-2.515098
C	-3.802351	4.136543	2.311861
H	-4.137295	4.687773	3.193375
C	5.550283	3.802126	-1.754063
H	5.468376	4.884195	-1.583522
H	5.488849	3.628895	-2.837933

H	6.552443	3.48537	-1.420683
C	-7.047925	-0.306007	-0.964261
H	-8.134297	-0.207815	-1.031055
C	4.045365	1.200094	-3.596711
H	2.946178	1.238038	-3.543944
H	4.344562	0.434635	-4.330517
H	4.419596	2.172946	-3.94945
C	4.132771	-0.950099	3.412973
H	3.240487	-1.590521	3.418669
H	3.969235	-0.127578	4.126507
H	5.015809	-1.514167	3.744356
C	-4.276947	1.716534	-3.673987
H	-5.31905	1.844662	-4.013454
H	-3.607611	1.975508	-4.510661
H	-4.082522	2.409322	-2.845529
C	2.715537	-2.446542	-3.111022
H	2.912931	-1.368441	-3.179607
H	1.932653	-2.690253	-3.844493
H	3.629579	-3.008266	-3.365673
C	-4.004314	4.672872	1.031049
H	-4.496371	5.641609	0.919048
C	4.574129	-4.690139	-0.624245
H	5.549153	-4.99339	-0.21699
H	4.644638	-4.706946	-1.722859
H	3.835316	-5.450572	-0.326738
C	2.058117	3.227934	2.6801
H	2.989615	2.767141	3.047004
H	1.254841	2.990454	3.393371
H	2.181439	4.31932	2.66113
C	5.930568	-3.057415	1.808102
H	5.560239	-3.403065	2.787087
H	6.650432	-2.246426	1.991657
H	6.475775	-3.893253	1.346631
C	-4.276344	-0.703142	-4.408944
H	-4.03981	-1.745222	-4.145466
H	-3.657607	-0.417545	-5.276416
H	-5.335578	-0.662045	-4.71464
C	1.7115	-4.256755	-1.610837
H	2.404921	-4.978098	-2.064028
H	0.761964	-4.303448	-2.160523
H	1.513068	-4.53914	-0.567459
C	-4.767604	-2.799075	1.98158
H	-5.856643	-2.973725	1.986191

H	-4.373691	-3.070739	2.976148
H	-4.314195	-3.473387	1.239972
H	1.968115	-0.129732	-1.537936

Table S3. Cartesian geometry of **1** in Angstrom [Å].

Atomtype	X coordinates	Y coordinates	Z coordinates
C	-1.486477	2.131927	4.310867
C	-1.436427	3.266771	3.499892
C	-1.345658	3.144836	2.099775
C	-1.316493	1.87089	1.540176
C	-1.377081	0.72065	2.361336
C	-1.454478	0.842988	3.745254
O	-1.230389	1.575988	0.221435
Si	-1.208684	-0.140067	-0.049428
N	-2.78521	-0.563839	-0.723669
C	-3.99	-0.093527	-0.076127
C	-4.617505	-0.879574	0.934898
C	-5.810149	-0.419702	1.512572
C	-6.392398	0.784684	1.128207
C	-5.772557	1.55466	0.148541
C	-4.578697	1.145487	-0.464183
C	-4.061924	-2.22177	1.414643
C	-4.966819	-3.404366	1.019842
O	-1.34808	-0.435726	1.657778
C	-3.978104	2.055288	-1.537634
C	-3.719331	3.478997	-1.013616
Ni	0.47365	-1.006655	-1.162522
Cl	1.393602	-2.211041	-2.77089
B	1.637836	-0.037034	0.211577
C	2.699665	-1.233211	0.371001
N	3.894654	-1.463722	-0.258455
C	4.488466	-2.623374	0.243737
C	3.634523	-3.130976	1.187252
N	2.542766	-2.267904	1.253929
C	4.392598	-0.650681	-1.393924
C	4.804211	-1.488054	-2.610977
C	1.331495	-2.447188	2.09352
C	0.615192	-3.770918	1.809832
C	3.795142	-4.389019	1.980099
C	5.805504	-3.184719	-0.18864
Si	-2.968528	-1.584124	-2.16299

C	-2.240364	-3.293711	-1.890475
C	-2.151723	-0.805422	-3.665545
C	-4.789928	-1.804703	-2.571119
C	-4.850213	2.120798	-2.806018
C	2.220965	1.444406	-0.118189
N	2.835521	2.230005	0.819232
C	3.164463	3.469749	0.270028
C	2.739271	3.447729	-1.033276
N	2.158358	2.197796	-1.250377
C	3.037511	1.811469	2.232121
C	2.358256	2.757841	3.225666
C	1.45691	1.764465	-2.489458
C	0.169074	2.556647	-2.72589
C	3.833879	4.592489	0.998488
C	2.876734	4.52374	-2.062615
C	4.505864	1.535881	2.571774
C	2.366438	1.715535	-3.718642
C	1.615002	-2.204975	3.579228
C	5.495704	0.326298	-0.973684
C	-3.819275	-2.24152	2.934237
H	-1.064771	-0.699592	-3.533046
H	-2.57961	0.184426	-3.889709
H	-2.30549	-1.448226	-4.548663
H	-5.297475	-0.855208	-2.789627
H	-5.353465	-2.296121	-1.766273
H	-4.866691	-2.44023	-3.469765
H	-2.767948	-3.833184	-1.088626
H	-1.170311	-3.251803	-1.637397
H	-2.32636	-3.890442	-2.814192
H	-3.007331	1.628695	-1.819313
H	-1.469328	4.261844	3.951095
H	-1.497485	-0.050567	4.371612
H	3.334112	1.24284	-3.496702
H	2.554993	2.708474	-4.153668
H	1.875129	1.102091	-4.488661
H	2.500488	0.859024	2.286641
H	1.153753	0.728649	-2.252709
H	0.354614	3.584538	-3.07356
H	-0.441481	2.586827	-1.813295
H	-0.414764	2.046893	-3.507303
H	-1.301792	4.025172	1.455017
H	-7.323414	1.122287	1.59146
H	0.651835	-1.656889	1.76534

H	-6.295831	-1.023942	2.283336
H	-6.227878	2.501591	-0.152893
H	4.84446	-4.713091	1.956754
H	3.190661	-5.214791	1.571449
H	3.512813	-4.262262	3.033869
H	-5.847495	2.537281	-2.58729
H	-4.997648	1.130042	-3.259061
H	-4.380293	2.768594	-3.565268
H	6.390317	-0.198807	-0.603776
H	5.154613	1.016451	-0.191401
H	5.803891	0.92729	-1.843487
H	-4.764755	-2.1921	3.499591
H	-3.189426	-1.399549	3.247759
H	-3.313753	-3.177377	3.227924
H	5.737797	-3.726046	-1.145331
H	6.167555	-3.89744	0.564796
H	6.575545	-2.40772	-0.299489
H	3.51294	-0.078019	-1.699089
H	-3.12453	3.464452	-0.091348
H	-4.660561	4.013295	-0.802429
H	-3.171666	4.070484	-1.76677
H	-3.088771	-2.364125	0.927765
H	2.101477	-1.230093	3.735177
H	2.24815	-2.982363	4.034068
H	0.660808	-2.191532	4.127349
H	3.311233	5.422017	-1.605655
H	1.909273	4.814399	-2.496035
H	3.534801	4.223317	-2.892526
H	-1.558274	2.239479	5.396224
H	1.125812	-4.646337	2.236299
H	0.493774	-3.920154	0.726448
H	-0.390255	-3.723757	2.254777
H	4.062262	-2.270689	-2.816898
H	5.805548	-1.927422	-2.506804
H	4.832177	-0.826367	-3.490412
H	4.187213	5.343464	0.27981
H	4.707298	4.258436	1.575877
H	3.149811	5.101525	1.695757
H	-5.119353	-3.46539	-0.067092
H	-5.960908	-3.323875	1.490096
H	-4.521496	-4.358378	1.349015
H	5.139784	2.432142	2.494577
H	4.9305	0.755442	1.925472

H	4.569917	1.178975	3.611424
H	1.324373	2.977012	2.923259
H	2.903081	3.70301	3.361176
H	2.313726	2.264027	4.208287
H	1.109948	0.10794	1.281619

Table S4. Cartesian geometry of **TS1** in Angstrom [Å].

Atomtype	X coordinates	Y coordinates	Z coordinates
N	1.783351	2.482789	-0.857299
C	1.948349	1.521118	0.092958
N	2.64118	2.129583	1.109653
C	2.911966	3.461188	0.792459
C	2.368179	3.683405	-0.448677
B	1.481983	-0.038556	0.164646
C	2.683184	-1.087834	0.093133
N	3.795273	-1.062059	-0.702976
C	4.665594	-2.095745	-0.355885
C	4.064818	-2.792173	0.666948
N	2.844306	-2.168703	0.923848
C	3.951452	-0.102814	-1.829679
C	4.885583	1.057794	-1.456721
C	5.992617	-2.360709	-0.997509
C	4.576632	-4.014292	1.366278
C	1.857389	-2.566732	1.968901
C	2.334615	-2.176198	3.377259
C	3.035981	1.432004	2.358637
C	4.53503	1.095821	2.36522
C	3.646882	4.428898	1.669779
C	2.40334	4.944851	-1.255525
C	0.97888	2.29148	-2.0941
C	1.780315	2.519921	-3.379196
Ni	0.39187	-0.898579	-1.387573
Cl	1.052422	-1.138246	-3.591514
Si	-1.18546	-0.232915	-0.043601
O	-1.279778	-1.165819	1.433658
C	-1.207915	-0.349548	2.520139
C	-1.117615	1.028896	2.195416
C	-1.037096	1.989457	3.205253
C	-1.04613	1.563185	4.551107
C	-1.125472	0.201817	4.869547
C	-1.206538	-0.771135	3.85009

O	-1.105846	1.27657	0.860622
N	-2.794707	-0.253083	-0.766088
Si	-3.179643	-0.598005	-2.471915
C	-5.054489	-0.480652	-2.735843
C	-3.92006	0.012191	0.107273
C	-4.563088	-1.064931	0.78075
C	-5.688142	-0.79314	1.578379
C	-6.181895	0.505295	1.725564
C	-5.538741	1.558232	1.070477
C	-4.409121	1.340316	0.263223
C	-4.092349	-2.51455	0.65822
C	-3.783083	-3.133969	2.037535
C	-3.761044	2.544059	-0.418904
C	-4.660543	3.180237	-1.501508
C	-5.116901	-3.395516	-0.089554
C	-3.3591	3.628338	0.602897
C	4.365227	-0.783373	-3.142144
C	1.462967	-4.047651	1.89083
C	-2.670141	-2.334647	-3.010072
C	-2.338659	0.655198	-3.613482
C	2.554989	2.153129	3.624467
C	-0.323276	3.093874	-2.018702
H	-1.242532	0.586993	-3.536144
H	-2.655134	1.684243	-3.376309
H	-2.607318	0.451852	-4.665037
H	-5.476399	0.505837	-2.501944
H	-5.608573	-1.215288	-2.133673
H	-5.262859	-0.693291	-3.799714
H	-3.202899	-3.116205	-2.444876
H	-1.58878	-2.505985	-2.903173
H	-2.912594	-2.466545	-4.079324
H	-2.847453	2.189322	-0.907806
H	-0.991337	2.307642	5.34891
H	-1.27687	-1.835364	4.084361
H	2.762012	2.026229	-3.333936
H	1.927841	3.586854	-3.607825
H	1.222441	2.063638	-4.20923
H	2.487479	0.490897	2.306565
H	0.694914	1.225062	-2.056304
H	-0.172415	4.176175	-2.149344
H	-0.821803	2.90957	-1.057251
H	-0.986041	2.749791	-2.824155
H	-0.969074	3.047659	2.945437

H	-7.059177	0.695695	2.349002
H	0.961524	-1.986447	1.738563
H	-6.18614	-1.620064	2.090586
H	-5.918332	2.576547	1.185125
H	5.65504	-4.120166	1.182946
H	4.086534	-4.931466	1.000632
H	4.423821	-3.966655	2.453443
H	-5.603551	3.548616	-1.063692
H	-4.912302	2.471601	-2.302292
H	-4.144452	4.040213	-1.962072
H	5.904683	0.700481	-1.238555
H	4.517161	1.614332	-0.585309
H	4.949725	1.753342	-2.307911
H	-4.699905	-3.245258	2.640848
H	-3.072602	-2.51085	2.596132
H	-3.34432	-4.138033	1.908556
H	5.89123	-2.867046	-1.970474
H	6.594345	-3.009535	-0.345463
H	6.560418	-1.433365	-1.162392
H	2.937515	0.277454	-1.992025
H	-2.744101	3.205436	1.406955
H	-4.248596	4.096852	1.056533
H	-2.783637	4.425253	0.10129
H	-3.160246	-2.517383	0.079403
H	2.610863	-1.115869	3.433307
H	3.197187	-2.777431	3.705153
H	1.510798	-2.342092	4.087061
H	2.8799	5.742731	-0.670251
H	1.398054	5.29408	-1.529711
H	2.978555	4.821746	-2.186101
H	-1.133379	-0.114832	5.91506
H	2.241832	-4.71885	2.27905
H	1.209757	-4.342243	0.863526
H	0.564941	-4.182856	2.51238
H	3.780254	-1.695988	-3.312318
H	5.44198	-1.002373	-3.180207
H	4.130476	-0.093708	-3.965464
H	3.936459	5.311984	1.084092
H	4.565773	3.996585	2.09112
H	3.02696	4.776562	2.511997
H	-5.32197	-3.017685	-1.101442
H	-6.074032	-3.436731	0.457328
H	-4.735339	-4.426552	-0.18209

H	5.16744	1.996252	2.360582
H	4.799344	0.476365	1.495805
H	4.769951	0.526273	3.27822
H	1.493486	2.423672	3.53545
H	3.149167	3.046684	3.862166
H	2.647742	1.457041	4.472605
H	1.010628	-0.132828	1.268142
C	0.394344	-2.827787	-1.173174
O	0.066433	-3.916506	-1.452411

Table S5. Cartesian geometry of **IM1** in Angstrom [Å].

Atomtype	X coordinates	Y coordinates	Z coordinates
N	2.169732	2.396963	-1.045477
C	2.225225	1.487145	-0.03702
N	2.967897	2.08092	0.952757
C	3.396071	3.343573	0.546975
C	2.891878	3.54307	-0.711685
B	1.564991	0.009657	0.126223
C	2.624535	-1.202037	0.164968
N	3.775779	-1.38811	-0.561766
C	4.465167	-2.508468	-0.101319
C	3.718238	-3.04905	0.912991
N	2.589747	-2.247697	1.061227
C	4.176457	-0.540419	-1.714316
C	5.26491	0.473232	-1.345368
C	5.766054	-3.014871	-0.639319
C	4.01753	-4.285694	1.699753
C	1.504862	-2.460888	2.056958
C	1.942048	-2.072358	3.473622
C	3.226354	1.459389	2.274452
C	4.665783	0.954882	2.420351
C	4.241923	4.27679	1.35575
C	3.096062	4.730837	-1.598234
C	1.315546	2.236079	-2.253258
C	2.088414	2.382867	-3.566542
Ni	0.340114	-1.18499	-1.173254
Cl	1.246559	-1.178761	-3.279024
Si	-1.209767	-0.171647	0.02278
O	-1.43769	-0.81306	1.60791
C	-1.36303	0.158825	2.551221
C	-1.145931	1.443631	2.00372

C	-1.011635	2.551651	2.835903
C	-1.113526	2.368377	4.228238
C	-1.337457	1.099879	4.765897
C	-1.464107	-0.022493	3.926182
O	-1.087293	1.44128	0.651532
N	-2.78344	-0.220418	-0.788162
Si	-3.160025	-0.519178	-2.507206
C	-5.025067	-0.454171	-2.758623
C	-3.937811	0.060429	0.052642
C	-4.569379	-1.006263	0.757738
C	-5.708878	-0.733596	1.527863
C	-6.237278	0.550787	1.62126
C	-5.613733	1.590884	0.939435
C	-4.468157	1.380036	0.156335
C	-4.079587	-2.454402	0.697753
C	-3.801235	-3.041375	2.092554
C	-3.86287	2.595518	-0.552703
C	-4.757518	3.140584	-1.683243
C	-5.057296	-3.36577	-0.068326
C	-3.548505	3.739991	0.428003
C	4.561184	-1.35484	-2.956484
C	0.90146	-3.870212	2.008292
C	-2.652739	-2.18713	-3.204716
C	-2.350831	0.760385	-3.619883
C	2.778571	2.342796	3.4431
C	0.087284	3.145665	-2.165142
H	-1.255556	0.658683	-3.59585
H	-2.623824	1.792168	-3.354627
H	-2.669195	0.584712	-4.661279
H	-5.515291	0.4537	-2.388498
H	-5.52598	-1.308266	-2.280821
H	-5.215927	-0.526045	-3.842892
H	-2.982258	-3.03964	-2.594969
H	-1.57011	-2.266901	-3.374103
H	-3.142566	-2.275648	-4.190276
H	-2.912579	2.271831	-0.995013
H	-1.02079	3.23261	4.891047
H	-1.646982	-1.018782	4.334599
H	3.034884	1.823267	-3.543689
H	2.299722	3.428738	-3.833544
H	1.481135	1.944421	-4.371091
H	2.564858	0.588184	2.270748
H	0.978967	1.192615	-2.194493

H	0.344724	4.214591	-2.21707
H	-0.466136	2.960117	-1.233409
H	-0.580416	2.926953	-3.009212
H	-0.839982	3.540264	2.405325
H	-7.128547	0.740861	2.22529
H	0.713693	-1.773696	1.747582
H	-6.196171	-1.551197	2.065161
H	-6.027235	2.599691	1.015365
H	5.072007	-4.562352	1.567566
H	3.411692	-5.145192	1.3709
H	3.845455	-4.152358	2.776444
H	-5.740022	3.458799	-1.296746
H	-4.936279	2.402405	-2.475937
H	-4.2862	4.021543	-2.150848
H	6.202892	-0.018381	-1.042224
H	4.952932	1.142981	-0.534095
H	5.491824	1.094401	-2.225868
H	-4.723182	-3.145583	2.688566
H	-3.096956	-2.41106	2.65156
H	-3.359618	-4.047587	1.997122
H	5.643148	-3.567795	-1.583784
H	6.222453	-3.702349	0.085524
H	6.486634	-2.205407	-0.821276
H	3.254186	-0.013529	-1.975684
H	-2.972733	3.388239	1.292491
H	-4.468582	4.214742	0.806955
H	-2.96428	4.526516	-0.078814
H	-3.129937	-2.460102	0.150088
H	2.320169	-1.041929	3.512314
H	2.720131	-2.740507	3.874604
H	1.073834	-2.127148	4.146948
H	3.578935	5.538064	-1.032475
H	2.151034	5.129273	-1.991451
H	3.739464	4.498075	-2.461379
H	-1.421775	0.972747	5.848113
H	1.502771	-4.618363	2.543549
H	0.746516	-4.213197	0.976952
H	-0.086308	-3.83487	2.491632
H	3.876028	-2.198843	-3.104952
H	5.601187	-1.708602	-2.930168
H	4.452113	-0.705425	-3.8372
H	4.67589	5.045706	0.703197
H	5.077067	3.761919	1.85025

H	3.663116	4.7955	2.136708
H	-5.228112	-3.014251	-1.095792
H	-6.038236	-3.416645	0.433089
H	-4.659073	-4.3922	-0.128767
H	5.406088	1.76927	2.399851
H	4.921954	0.237054	1.629254
H	4.771169	0.440335	3.388109
H	1.763892	2.731336	3.276405
H	3.462345	3.179764	3.641677
H	2.743469	1.72687	4.354883
H	1.078659	0.053517	1.223753
C	-0.448162	-2.803818	-1.083783
O	-0.937728	-3.843844	-1.034684

Table S6. Cartesian geometry of **TS2** in Angstrom [Å].

Atomtype	X coordinates	Y coordinates	Z coordinates
N	2.522908	2.662684	0.137909
C	2.976209	1.475071	-0.383199
N	4.261674	1.682965	-0.76741
C	4.647968	2.977774	-0.430867
C	3.585693	3.542461	0.143232
B	2.260049	-0.044214	-0.498437
C	3.12365	-1.153947	0.189482
N	3.621308	-1.113675	1.487568
C	4.358801	-2.255585	1.768471
C	4.294548	-3.049436	0.667429
N	3.567422	-2.346226	-0.30386
C	3.317823	-0.001942	2.405169
C	4.580607	0.778383	2.811115
C	5.068569	-2.531113	3.045745
C	4.844793	-4.436669	0.458353
C	3.299072	-2.746395	-1.741099
C	2.382728	-3.972863	-1.772395
C	5.065767	0.729782	-1.536876
C	6.239701	0.140971	-0.758234
C	5.960333	3.634068	-0.743418
C	3.479159	4.971034	0.695773
C	1.086292	2.961923	0.446782
C	0.851671	3.039463	1.966711
Ni	0.084953	-0.328669	-1.236422
Cl	0.41895	-1.211843	-3.260958

Si	-1.989649	0.150962	-0.719392
O	-2.927268	0.949293	-1.957472
C	-3.342553	2.157373	-1.541201
C	-2.904591	2.481673	-0.267037
C	-3.197817	3.687084	0.390574
C	-3.983627	4.592664	-0.372297
C	-4.471965	4.298477	-1.642146
C	-4.097636	3.069715	-2.227398
O	-2.119944	1.543584	0.355472
N	-2.982953	-1.099187	0.04308
Si	-2.568775	-2.784185	-0.117491
C	-3.858793	-3.784373	0.806225
C	-4.208437	-0.6687	0.690499
C	-5.42056	-0.610537	0.003376
C	-6.560573	-0.175211	0.656605
C	-6.526496	0.112945	2.055223
C	-5.324244	0.08775	2.673798
C	-4.146767	-0.385356	2.094815
C	-5.569569	-0.952112	-1.474912
C	-6.309591	0.118256	-2.297429
C	-2.870305	-0.426065	2.887764
C	-2.975731	-1.580722	3.934007
C	-6.255906	-2.332715	-1.719548
C	-2.526308	0.88794	3.583649
C	0.340702	-0.045499	0.628183
O	0.249175	-0.025372	1.810362
C	-2.575339	-3.363428	-1.894407
C	-0.919912	-3.191558	0.681072
C	2.622386	-0.487848	3.699306
C	4.603198	-2.926041	-2.507767
C	5.481221	1.30423	-2.908177
C	0.618129	4.21245	-0.323711
H	4.433223	-0.063671	-1.742461
H	6.922773	0.873813	-0.541954
H	6.692682	-0.585824	-1.321433
H	5.901816	-0.271336	0.117024
H	2.678719	0.650204	1.919354
H	-4.388957	2.859482	-3.166184
H	-2.099978	-0.659507	2.237004
H	2.782471	-1.96291	-2.180464
H	-4.199639	5.489897	0.026285
H	-7.367058	0.327438	2.561254
H	-4.612935	-1.021311	-1.867675

H	4.407149	5.295166	0.983154
H	2.851954	4.984213	1.506216
H	3.109474	5.598225	-0.024747
H	5.135667	0.222427	3.469552
H	4.307323	1.658581	3.259361
H	5.144469	0.981228	1.981249
H	-5.071426	4.940648	-2.127263
H	6.333382	1.865752	-2.8119
H	4.728902	1.894255	-3.27977
H	5.657686	0.533516	-3.561494
H	5.93704	4.024563	-1.691247
H	6.719196	2.949716	-0.679967
H	6.130815	4.389701	-0.07123
H	0.53781	2.160365	0.08798
H	-5.275407	0.434475	3.615982
H	-3.680564	-1.343487	4.638523
H	-2.06778	-1.712205	4.392478
H	-3.245873	-2.45051	3.46429
H	-7.417198	-0.060661	0.144684
H	-0.187258	-2.586272	0.296936
H	-0.681535	-4.17052	0.490008
H	-0.980607	-3.048481	1.695219
H	1.007103	2.120704	2.390902
H	-0.112839	3.334976	2.144757
H	1.498005	3.716772	2.383954
H	-2.5493	1.654427	2.903989
H	-1.586885	0.823931	3.989687
H	-3.205212	1.075093	4.329737
H	-3.797636	-3.58461	1.810483
H	-3.694289	-4.784329	0.649238
H	-4.796829	-3.539441	0.475331
H	1.070772	4.24126	-1.243441
H	0.860701	5.059828	0.197155
H	-0.398439	4.172405	-0.447866
H	-7.322873	0.016586	-2.171344
H	-6.081704	0.006082	-3.291256
H	-6.025195	1.052996	-1.990196
H	2.082681	-4.147659	-2.735545
H	1.559994	-3.800035	-1.186406
H	2.883404	-4.794748	-1.418851
H	1.873758	-1.146839	3.465352
H	2.224111	0.31711	4.194444
H	3.303383	-0.9517	4.307226

H	5.107222	-3.748537	-2.16433
H	5.19099	-2.095383	-2.388498
H	4.39421	-3.050124	-3.504438
H	5.551164	-4.634548	1.17494
H	5.274652	-4.508835	-0.465818
H	4.087881	-5.124639	0.533154
H	4.408251	-2.507329	3.827684
H	5.798073	-1.828093	3.198751
H	5.501018	-3.458755	3.000702
H	-5.745598	-3.068548	-1.222126
H	-6.257705	-2.540758	-2.723892
H	-7.222238	-2.302864	-1.378235
H	-3.539955	-3.425106	-2.235017
H	-2.134375	-4.287468	-1.953838
H	-2.050146	-2.704266	-2.478289
H	2.294249	-0.071409	-1.821856
H	-2.811899	3.860903	1.394269

Table S7. Cartesian geometry of **2-CO** in Angstrom [Å].

Atomtype	X coordinates	Y coordinates	Z coordinates
C	-3.122836	3.723549	0.522192
C	-2.786628	2.53113	-0.124679
C	-3.23521	2.268012	-1.443917
C	-3.997072	3.212345	-2.136953
C	-4.329791	4.420673	-1.487177
C	-3.90362	4.672111	-0.174072
O	-2.00011	1.545665	0.400983
Si	-1.903463	0.192128	-0.728949
N	-2.882388	-1.06677	0.006514
C	-4.0769	-0.682638	0.724494
C	-5.331112	-0.649224	0.055628
C	-6.489226	-0.358419	0.800952
C	-6.424397	-0.087868	2.171908
C	-5.180196	-0.090505	2.813566
C	-3.995274	-0.376362	2.112354
C	-5.466909	-0.922527	-1.44188
C	-6.22072	-2.244522	-1.716276
C	-2.665062	-0.345265	2.862154
C	-2.450464	1.006629	3.579181
O	-2.846003	1.053281	-1.933817
Ni	0.113092	-0.355983	-1.348572

B	2.213181	0.028491	-0.470946
C	0.698371	-0.129319	0.302247
O	0.480894	-0.192602	1.502288
Cl	0.202821	-1.20981	-3.41245
Si	-2.522448	-2.806319	-0.105373
C	-2.57971	-3.404659	-1.904909
C	-0.794092	-3.179288	0.610265
C	-3.812381	-3.754915	0.925942
C	-2.54576	-1.502658	3.879674
C	-6.181713	0.243172	-2.16278
C	3.104642	-1.128586	0.196975
N	3.588318	-1.128552	1.480014
C	4.21407	-2.348988	1.756572
C	4.090867	-3.123638	0.627163
N	3.404743	-2.359204	-0.320474
C	3.39402	0.001419	2.433232
C	2.727498	-0.455011	3.741596
C	3.02277	-2.794005	-1.697495
C	4.248653	-2.860902	-2.623573
C	4.846444	-2.718582	3.06514
C	4.535214	-4.541609	0.429008
C	4.694268	0.790839	2.66059
C	2.206226	-4.096567	-1.693533
C	2.90378	1.491784	-0.366211
N	4.21585	1.722578	-0.695259
C	4.560431	3.044687	-0.397795
C	3.425795	3.645044	0.093918
N	2.41334	2.680171	0.084662
C	5.029577	0.74722	-1.475414
C	5.546117	1.367266	-2.78603
C	0.958157	2.921696	0.370049
C	0.422153	4.125908	-0.419981
C	3.266486	5.057241	0.572027
C	5.919335	3.650354	-0.586798
C	6.146575	0.098341	-0.642751
C	0.67297	2.996537	1.873209
H	4.313744	-0.031582	-1.752845
H	6.888274	0.838143	-0.306572
H	6.666994	-0.641482	-1.270794
H	5.737864	-0.419417	0.234109
H	2.676911	0.657591	1.939925
H	-4.325807	3.000397	-3.155284
H	-1.856486	-0.458343	2.134767

H	2.354508	-2.016284	-2.072713
H	-4.177264	5.60747	0.319177
H	-7.335165	0.132675	2.734419
H	-4.458616	-1.005512	-1.864826
H	4.252323	5.469921	0.83216
H	2.626862	5.113939	1.463154
H	2.823443	5.707697	-0.198931
H	5.47529	0.177807	3.134914
H	4.476155	1.638347	3.329262
H	5.081936	1.189149	1.71405
H	-4.930404	5.163983	-2.016114
H	6.402216	2.03643	-2.623588
H	4.744282	1.919928	-3.297659
H	5.877098	0.550218	-3.445399
H	6.089499	3.97984	-1.625182
H	6.723049	2.9484	-0.322932
H	6.019072	4.533961	0.060066
H	0.446892	2.041099	-0.028592
H	-5.120665	0.134527	3.881503
H	-3.368917	-1.467968	4.613644
H	-1.591089	-1.425295	4.429347
H	-2.573099	-2.482121	3.379169
H	-7.454537	-0.342525	0.289429
H	-0.01804	-2.73694	-0.034645
H	-0.620789	-4.269863	0.668576
H	-0.671963	-2.744373	1.614467
H	1.057673	2.106681	2.389101
H	-0.417472	3.001629	2.001692
H	1.09466	3.905149	2.332348
H	-2.576212	1.836704	2.87122
H	-1.429623	1.047088	3.995997
H	-3.164151	1.13917	4.41043
H	-3.78412	-3.474404	1.989742
H	-3.614228	-4.839299	0.852516
H	-4.839417	-3.572064	0.576315
H	0.754566	4.084676	-1.468842
H	0.720574	5.088065	0.01928
H	-0.675242	4.072804	-0.403722
H	-7.238737	0.310194	-1.853057
H	-6.157522	0.078694	-3.253138
H	-5.69454	1.201741	-1.940022
H	1.661564	-4.145843	-2.647619
H	1.462907	-4.086506	-0.884579

H	2.839881	-4.990937	-1.599568
H	1.867007	-1.099315	3.520424
H	2.354757	0.441148	4.261168
H	3.426925	-0.970115	4.414752
H	4.930074	-3.682634	-2.355759
H	4.815622	-1.91818	-2.60666
H	3.894486	-3.030491	-3.651505
H	5.232111	-4.825093	1.230306
H	5.047547	-4.688832	-0.532072
H	3.68366	-5.239833	0.46192
H	4.094804	-3.000059	3.820869
H	5.44435	-1.896448	3.483231
H	5.514069	-3.579864	2.921319
H	-5.683384	-3.111194	-1.304287
H	-6.335887	-2.396836	-2.803204
H	-7.227209	-2.217387	-1.265198
H	-3.579181	-3.251405	-2.344091
H	-2.343721	-4.48288	-1.968194
H	-1.847962	-2.859366	-2.523418
H	2.077388	-0.175365	-1.672852
H	-2.786644	3.903569	1.545283

Table S8. Cartesian geometry of CO in Angstrom [Å].

Atomtype	X coordinates	Y coordinates	Z coordinates
C	0	0	-0.655445
O	0	0	0.491584

Table S9. Cartesian geometry of **4-Cy** in Angstrom [Å].

Atomtype	X coordinates	Y coordinates	Z coordinates
C	-6.278145	-0.429996	-0.469489
C	-5.353773	0.690896	0.051818
C	-4.423607	0.162727	1.162959
C	-3.601375	-1.064181	0.702947
C	-4.522004	-2.169605	0.143289
C	-5.456284	-1.639042	-0.964123
N	-2.658071	-0.628527	-0.365801
C	-1.467016	-0.389892	-0.189112
B	-0.05205	-0.07501	-0.077923
C	1.02928	-1.183929	-0.334157
N	2.141267	-1.069625	-1.132053

C	2.862968	-2.2685	-1.124553
C	2.183587	-3.139431	-0.304405
N	1.068661	-2.451489	0.184305
C	2.324818	0.064836	-2.083719
C	3.729219	0.683825	-2.01791
C	0.046339	-2.953753	1.1424
C	-0.974445	-3.848708	0.422715
C	4.154521	-2.496363	-1.85045
C	2.525551	-4.559159	0.034615
C	0.667721	-3.612459	2.385743
C	1.90477	-0.365758	-3.499202
C	0.404278	1.379399	0.299933
N	0.028442	2.561777	-0.28115
C	0.692952	3.629928	0.327102
C	1.516314	3.09371	1.290817
N	1.316794	1.710087	1.270955
C	-0.949558	2.623022	-1.403768
C	-2.328873	3.065546	-0.891522
C	1.846773	0.710062	2.240491
C	1.632164	1.14663	3.700653
C	2.459912	3.818049	2.204657
C	0.50847	5.074866	-0.029362
C	-0.436358	3.466668	-2.583023
C	3.307478	0.343299	1.93694
H	-0.471045	-2.039249	1.467113
H	1.611194	0.822179	-1.744456
H	-1.026317	1.579952	-1.738875
H	0.940738	5.70594	0.758538
H	-0.555928	5.335245	-0.1195
H	0.997731	5.336582	-0.981368
H	1.221781	-0.175858	2.066212
H	2.566058	-1.144198	-3.905527
H	1.949875	0.505288	-4.171026
H	0.873056	-0.747767	-3.485522
H	-0.522875	4.545167	-2.396931
H	-1.048761	3.229972	-3.465857
H	0.613039	3.22742	-2.816063
H	4.977086	-1.909387	-1.410639
H	4.090083	-2.232371	-2.916007
H	4.431163	-3.556849	-1.784752
H	1.026038	-4.631413	2.190031
H	-0.1091	-3.676508	3.162441
H	1.500904	-3.010085	2.780136

H	-2.672592	2.396983	-0.090335
H	-3.05453	3.015545	-1.717337
H	-2.308223	4.099352	-0.514861
H	3.272726	-4.940707	-0.674129
H	1.642048	-5.210759	-0.026644
H	2.943779	-4.655865	1.049175
H	-1.420936	-3.311955	-0.426094
H	-1.777519	-4.11847	1.125974
H	-0.5122	-4.778403	0.057827
H	2.658828	4.82189	1.805889
H	3.424041	3.296445	2.290196
H	2.050402	3.933783	3.220959
H	3.404754	-0.054554	0.918204
H	3.63933	-0.430387	2.646709
H	3.973486	1.211758	2.048839
H	4.012245	0.914078	-0.980546
H	3.717068	1.625392	-2.588045
H	4.494727	0.03519	-2.4627
H	2.358346	1.905349	4.020843
H	1.765725	0.265049	4.345812
H	0.613838	1.53491	3.85059
H	-4.741005	1.072131	-0.781888
H	-5.948735	1.534022	0.438449
H	-3.019873	-1.451878	1.560693
H	-5.123326	-2.551364	0.986811
H	-3.910033	-3.006406	-0.229869
H	-5.029992	-0.157545	2.027904
H	-3.734877	0.948551	1.516227
H	-4.844109	-1.333597	-1.828866
H	-6.124882	-2.447185	-1.30103
H	-6.949019	-0.754069	0.346815
H	-6.917668	-0.04798	-1.281404

1 Kuhn, N., and Kratz, T., *Synthesis*, **1993**, 561.
2 Cui, H., and Cui, C., *Dalton Trans.*, **2011**, *40*, 11937.
3 Hadlington, T. J., Szilvasi, T., and Driess, M., *Angew. Chem. Int. Ed.* **2017**, *56*, 7470-7474.
4 Goodwin, C. A. P., Smith, A., Ortu, F., Vitorica-Yrezabal, I. J., and Mills, D. P., *Dalton Trans.*, **2016**, *45*, 6004-6014.
5 Sheldrick, G. M., *Acta Cryst.*, **2015**, *A71*, 3-8.
6 Sheldrick, G. M., *Acta Cryst.*, **2015**, *C71*, 3-8.
7 Spek, A. L., *Acta Cryst.*, **2015**, *C71*, 9–18.
8 Frisch, M. J., Pople, J. A., Binkley, J. S. J. Chem. Phys., 1984, 80, 3265-3269; A. D. Becke, J. Chem. Phys., 1997, 107, 8554-8560; S. Grimme, J. Comput. Chem., 2006, 27, 1787-1799; T. H. Dunning, J. Chem. Phys., 1989, 90, 1007-1023.
9 Xiong, Y., Szilvási, T., Yao, S., Tan, G., and Driess, M., *J. Am. Chem. Soc.*, **2014**, *136*, 11300; Hansen, K., Szilvási, T., Blom, B., and Driess, M., *J. Am. Chem. Soc.*, **2014**, *136*, 14207; Hansen, K., Szilvási, T., Blom, B., Irran, E., and Driess, M., *Chem. Eur. J.*, **2014**, *20*, 1947; Hansen, K., Szilvási, T., Blom, B., Inoue, S., Epping, J., and Driess, M., *J. Am. Chem. Soc.*, **2013**, *135*, 11795.

10 Gaussian 09, Revision D.01, Frisch, M. J., Trucks, G. W., Schlegel, H. B., Scuseria, G. E., Robb, M. A., Cheeseman, J. R., Scalmani, G., Barone, V., Mennucci, B., Petersson, G. A., Nakatsuji, H., Caricato, M., Li, X., Hratchian, H. P., Izmaylov, A. F., Bloino, J., Zheng, G., Sonnenberg, J. L., Hada, M., Ehara, M., Toyota, K., Fukuda, R., Hasegawa, J., Ishida, M., Nakajima, T., Honda, Y., Kitao, O., Nakai, H., Vreven, T., Montgomery, J. A., Jr., J. E. P., Ogliaro, F., Bearpark, M., Heyd, J. J., Brothers, E., Kudin, K. N., Staroverov, V. N., Kobayashi, R., Normand, J., Raghavachari, K., Rendell, A., Burant, J. C., Iyengar, S. S., Tomasi, J., Cossi, M., Rega, N., Millam, J. M., Klene, M., Knox, J. E., Cross, J. B., Bakken, V., Adamo, C., Jaramillo, J., Gomperts, R., Stratmann, R. E., Yazyev, O., Austin, A. J., Cammi, R., Pomelli, C., Ochterski, J. W., Martin, R. L., Morokuma, K., Zakrzewski, V. G., Voth, G. A., Salvador, P., Dannenberg, J. J., Dapprich, S., Daniels, A. D., Farkas, Ö., Foresman, J. B., Ortiz, J. V., Cioslowski, J., Fox, D. J., Gaussian, Inc., Wallingford CT, 2009.
